# Promoting CO_2_ reduction in the presence of oxygen with polymer-based gas diffusion electrodes

**DOI:** 10.1016/j.checat.2025.101353

**Published:** 2025-07-17

**Authors:** Sam Van Daele, Lieven Hintjens, Daniel Choukroun, Nick Daems, Jonas Hereijgers, Tom Breugelmans

**Affiliations:** 1 Research Group Applied Electrochemistry & Catalysis (ELCAT), University of Antwerp, Faculty of Applied Engineering, Universiteitsplein 1, 2610 Wilrijk, Antwerp, Belgium

**Keywords:** CO_2_ reduction, gas impurities, gas diffusion electrode, electrochemistry, CO, formate, CO_2_ conversion, electrode design

## Abstract

The electrochemical reduction of CO_2_ is a promising technology that holds the potential to convert waste CO_2_ into valuable products. High carbon capture and purification costs hamper economic feasibility and drive scientists to explore the viability of directly using flue gas exhaust streams. However, flue gas impurities, such as O_2_, pose a great challenge because O_2_ is preferentially reduced over CO_2_. Here, we show that careful design of the gas diffusion electrode (GDE) can significantly improve Faradaic efficiency. This work not only unravels how commonly used carbon-based GDEs facilitate O_2_ reduction but also succeeds in devising polymer-based alternatives that significantly improve the Faradaic efficiency (>40%) of CO_2_ reduction with 5% O_2_-containing feed streams while showing excellent stability for >2 days. These results demonstrate that it is feasible to engineer suitable GDEs for CO_2_ reduction with impure feed streams.

## Highlights

• Unravelling the role of the GDL material during CO_2_ reduction with O_2_ impurities
• Limiting the parasitic oxygen evolution reaction with PTFE gas diffusion electrodes
• 50 h stable electrolyzer operation with an impure CO_2_ feed stream

## The bigger picture

CO_2_ emissions drive climate change and require urgent abatement. Direct electrochemical conversion of greenhouse gas into value-added chemicals presents great prospects, yet the inherently high costs of CO_2_ capture and purification weigh on its profitability. One solution is to directly use flue gas exhaust streams that contain CO_2_ with unreacted O_2_, but the competition between the two for electrons hampers the efficiency of the process. Here, we develop a polymer-based electrode architecture that provides the right balance of in-plane conductivity and optimal CO_2_ conversion at the expense of O_2_ diffusion, thus suppressing the major parasitic reactions. As a case in point, our electrodes boost efficiency by more than 40% at a high O_2_ concentration of 5% and maintain stability for >2 days. This breakthrough is bound to enable the direct utilization of waste CO_2_ streams from a multitude of industrial point sources.

## Graphical abstract

**Figure:**
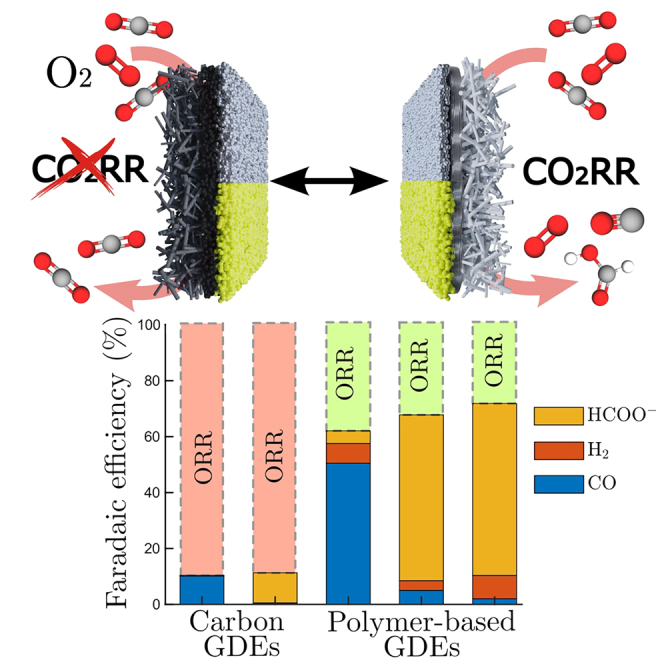

## Teaser

Polymer-based gas diffusion electrodes (GDEs) allow for more oxygen-tolerant CO_2_ electroreduction than conventional carbon-based GDEs. These electrodes enable impurity-resistant CO_2_ conversion to valuable products, reducing the need for extensive purification steps. This advancement boosts the economic viability of electrochemical CO_2_ reduction toward industrial applications.

## Introduction

In the face of global concerns regarding climate change, reducing CO_2_ emissions stands out as one of the important challenges of our time.^1^ The pressing need to mitigate CO_2_ emissions has led to an increasing interest in carbon capture and utilization (CCU) technologies, such as the electrochemical CO_2_ reduction reaction (CO_2_RR), which holds the potential to convert CO_2_ into useful chemicals and fuels.^2^ An additional benefit of this electrochemical process is its compatibility with an intermittent electricity supply from renewable energy sources (e.g., solar or wind power).^3^ Converting CO_2_ into CO and formate or formic acid is of special interest in this work because these reaction products benefit from high revenue per mole of electron transferred and can both be produced at a Faradaic efficiency (FE) exceeding 90%.^4^^,^^5^

**Figure 1:**
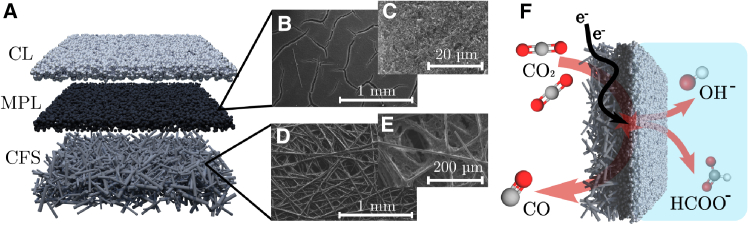
Illustration of a gas diffusion electrode (A) Representation of the carbon fiber support (CFS), microporous layer (MPL), and catalyst layer (CL). (B and C) Scanning electron microscopy (SEM) images of the MPL. (D and E) SEM images of the CFS. (F) Schematic of the gas diffusion electrode (GDE) during CO_2_ electrolysis.

One of the most important advances in CO_2_ reduction research was the transition from the conventional batch H-cell, where the system was limited by the sluggish mass transport of CO_2_ in aqueous electrolytes, to more advanced continuous-flow cell configurations (CO_2_ electrolyzers) that ensure shorter CO_2_ diffusional lengths.^6^^,^^7^ The key to this transition was the employment of gas diffusion electrodes (GDEs) that allow a supply of gaseous CO_2_ to the cell and thereby improve CO_2_ mass transport to facilitate electrolyzer operation at current densities well above 100 mA cm^*−*2^ to CO_2_RR products.^8^ Figure 1A illustrates the structure of a typical carbon-based GDE consisting of a carbon fiber support (CFS) topped with a microporous layer (MPL), whereupon catalytic particles are coated to form the catalyst layer (CL).^9^ The MPL (Figures 1B and 1C) consists of carbon that has undergone a polymeric treatment, usually with polytetrafluorethylene (PTFE), to increase the hydrophobic properties of this layer and prevent liquid from penetrating the GDE.^10^ This is an essential property because excessive electrolyte seepage through the GDE (i.e., flooding) limits the stability of electrolyzer operation.^11^ The CFS (Figures 1D and 1E) consists of a macroporous carbon fiber paper and governs the overall mechanical properties of the GDE. In this study, when referring to a carbon-based GDE, we utilized the Sigracet 39BB gas diffusion layer (GDL; CFS + MPL), which contains 5% (w/w) PTFE in the CFS and 20%–25% (w/w) PTFE in the MPL according to the manufacturer’s technical properties sheet.^12^ A comparative study between different PTFE GDEs was conducted elsewhere.^13^

During electrolyzer operation, gaseous CO_2_ from the feed stream diffuses through the CFS and MPL to reach the CL, where CO_2_ adsorbs to its active sites and the CO_2_RR can take place. Typical catalysts for the reaction toward CO (Equation 2) are Ag^11^ and Au,^14^ whereas formate is selectively produced (Equation 1) with mainly Bi^15^ and Sn^16^ catalysts. The CL exchanges gaseous species through the porous structure of the dry MPL and exchanges ionic species with the neighboring liquid phase (Figure 1F).^17^ When the CO_2_ supply to the CL’s active sites is insufficient or no CO_2_RR catalyst is present, H_2_ is generated via the hydrogen evolution reaction (HER) (Equation 3). The CO_2_RR and HER yield OH^*−*^ as byproducts, which can react with incoming CO_2_ to form bicarbonate or carbonate according to Equation 4 or 5, respectively.

(Equation 1) $${\text{CO}}_{2}+H_{2}O+2e^{-}\to \;\text{HCO}O^{-}+OH^{-}$$

(Equation 2) $${\text{CO}}_{2}+H_{2}O+2e^{-}\to \;\text{CO}+2OH^{-}$$

(Equation 3) $$2H_{2}O+2e^{-}\to H_{2}+2OH^{-}$$

(Equation 4) $$OH^{-}+{\text{CO}}_{2}\;⇋\;\text{HC}O_{3}^{-}$$

(Equation 5) $$\text{HC}O_{3}^{-}+OH^{-}\;⇋\;CO_{3}^{2-}+{\;H}_{2}O$$

Over the last few years, different pilot-scale CO_2_ electrolysis plants based on the utilization of purified CO_2_ have been installed all over the world.^18^ However, the costs associated with the CO_2_-capture and -purification processes limit the economic viability of the technology.^19^^,^^20^ Therefore, direct CO_2_ conversion from flue gas has emerged as an interesting avenue for potentially bypassing the carbon-capture procedure or significantly reducing the number of purification steps necessary. This approach presents its own set of challenges, specifically the compatibility of the CO_2_RR with flue gas impurities.

In flue gas point sources, CO_2_ is typically present in a diluted form (13%–14% CO_2_) with mainly >70% N_2_, 3%–4% O_2_, ∼200 ppm SO_x_, and ∼200 ppm NO_x_ impurities (Singh and Berchtold, 2018, Project Review Meeting for Crosscutting Research Portfolios). Previous work has shown that diluted CO_2_ streams lead to electrolyzer operation at a lower partial current density (CD) to C-products because there is limited CO_2_ near the electrode.^21^^,^^22^ The impacts of SO_x_^23^ and NO_x_^24^ during CO_2_ electrolysis in continuous-flow cells have also been studied.^25^ Because of their low abundance in flue gases, electrolyzer operation at 100 mA cm^*−*2^ for 20 h results in a stable operation without significant loss of FE or catalyst degradation.^26^ O_2_ in flue gas streams was initially hypothesized to be incompatible with the CO_2_RR given the preferential reduction of O_2_.^27^ Indeed, we recently observed a significant loss of FE when we investigated the impact of O_2_ during CO_2_ electrolysis.^26^ Preventing the oxygen reduction reaction (ORR) to sustain high FE for the CO_2_RR remains a top priority in advancing the field of direct CO_2_ electrolysis from flue gases, which is the aim of our present study.

In order to limit the ORR during the CO_2_RR with O_2_ impurities in the gas stream, this work delves into the role of the carbon-containing substrate material in facilitating the ORR during the CO_2_RR, which has never—to the best of our knowledge—been studied before. The upcoming use of metal-free carbon GDEs in the field of oxygen reduction motivated a thorough exploration of the GDL material.^28^^,^^29^ Here, we show that careful engineering of the GDE allows for a significant improvement in FE to CO_2_RR products in the presence of O_2_. Polymer-based GDEs effectively suppress the ORR on the GDE substrate itself and allow for a substantial increase in FE with O_2_-containing CO_2_ feed streams. In fact, any undesired electrochemical reaction that can take place outside the CL is prevented through the use of a non-conductive polymer-based GDL. This knowledge is critical for the future implementation of this technology and could allow industrial plants to bypass or limit the number of necessary purification steps.

## Results and discussion

### Role of the GDE substrate material

**Figure 2:**
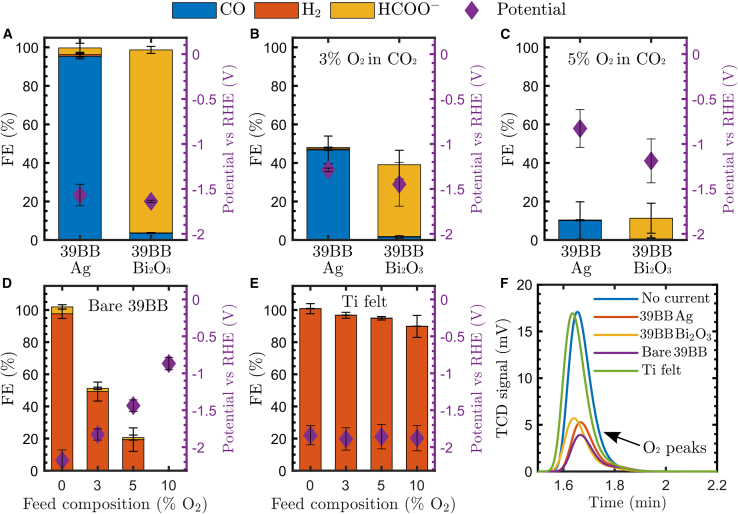
Impact of the GDE substrate material with O_2_-containing CO_2_ feed streams (A–C) Ag- and Bi_2_O_3_-coated carbon paper (Sigracet 39BB) at 100 mA cm^*−*2^ with 0% (A), 3% (B), and 5% (C) O_2_ in the CO_2_ feed stream. (D) Carbon paper without catalyst at 100 mA cm^*−*2^ with varying O_2_ concentrations in the CO_2_ feed. (E) PTFE-treated Ti felt without catalyst at 100 mA cm^*−*2^ with varying O_2_ concentrations in the CO_2_ feed. (F) GC slice at the retention time for O_2_ detection with and without application of 100 mA cm^*−*2^ with 3% O_2_ in the CO_2_ feed stream for different GDEs. Error bars represent the standard deviation from three measurements.

Initially, we examined the impact of various oxygen concentrations in the CO_2_ feed stream on the FE. This investigation was conducted at a CD of 100 mA cm^*−*2^ for different oxygen concentrations (0%, 3%, 5%, 10%, and 20% O_2_ in CO_2_) that were fed to the reactor during a single run, and the results are shown in Figures 2A–2C. With a pure CO_2_ feed (Figure 2A), the Ag-coated carbon paper produced CO at FE_CO_ = 95.3% ± 1.4%, and the Bi_2_O_3_-coated carbon paper produced HCOO^*−*^ at FE_HCOO^*−*^_ = 95.0% ± 1.8%, both comparable to the state-of-the-art efficiencies with GDEs in flow cells.^30^^,^^31^ The addition of 3% O_2_ caused a >50% loss of total FE (Figure 2B), consistent with previous work.^26^ The data of the complete run can be found in Figures S1 and S2. The FE decreased because the competitive ORR has a lower thermodynamic potential than the CO_2_RR.^32^ When the oxygen content increased even more to 5% (Figure 2C), the potential shifted to a less negative value because the ORR consumed almost all electrons (i.e., only ∼10% total FE was detected). We observed a potential difference (working electrode) between 39BB-Ag and 39BB-Bi_2_O_3_ because the former effectively catalyzes the ORR (i.e., it has a lower overpotential than Bi_2_O_3_ at the same CD), such that a lower overpotential is required for facilitating the ORR.^26^^,^^33^ In this and previous work^26^ with Ag and Bi_2_O_3_ catalysts, the total FEs for 39BB-Ag and 39BB-Bi_2_O_3_ were comparable, demonstrating that electron consumption by the ORR is similar for both target products. This indicates that O_2_ availability at active sites is the limiting factor for the ORR and is correlated with O_2_ feed composition.^26^ It is worth mentioning that for copper catalysts in an H-cell, including O_2_ in the CO_2_ feed can increase the surface coverage of adsorbed hydroxyl species, improving the production rates of hydrocarbons and oxygenates.^34^ In general, the formation of OH^*−*^ (from the CO_2_RR, HER, or ORR) contributes to the local alkalinity at the electrode, which can both consume CO_2_ to form HCO^*−*^ and even promote the generation of C_2+_ products with copper catalysts.^35^^,^^36^^,^^37^

We hypothesize that the carbon-based substrate material (Sigracet 39BB) also plays a crucial role in facilitating the ORR on GDEs for CO_2_ reduction. In the field of proton-exchange membrane fuel cells, metal-free carbon catalysts are indeed gaining interest for an efficient ORR.^28^^,^^29^ This suggests that carbon-based substrates might also facilitate the ORR and could therefore decrease the overall FE during the CO_2_RR. To this end, we used a bare 39BB carbon paper without a catalyst as the GDE at 100 mA cm^*−*2^ and altered the feed composition every 25 min (Figure 2D). With pure CO_2_, the near-unity FE (97.6% ± 3.0%) was attributed to the HER, as expected in the absence of a CO_2_RR catalyst. Interestingly, adding oxygen to the feed also lowered the overpotential and total FE, as observed in experiments with catalyst-coated 39BB carbon paper (Figures 2A–2C). To investigate the substrate effect further, we opted to test a Ti felt GDE (scanning electron microscopy [SEM] images in Figure S3). Because untreated Ti felt allows liquid to pass through, we treated the Ti felt with PTFE to provide a hydrophobic gas-liquid barrier. After the treatment, we achieved a static contact angle of 115° (Figure S4). The bare Ti felt electrode (without a catalyst) exclusively produced H_2_ under pure CO_2_ conditions. Interestingly, when we increased the O_2_ feed concentration, the FE remained high. For example, the FE_H2_ still reached a value of 89.8% ± 6.8% even with 10% O_2_ in CO_2_. This observation highlights the importance of the type of substrate material. Although the bare Ti felt indicates a difference in ORR activity, the substrate cannot be used for efficient CO_2_ electrolysis, as indicated by the additional Ag-coated Ti felt measurements in Figure S5. The FE_CO_ remained <40% as a result of the absence of a MPL^9^ and the activity of Ti toward the HER.^38^ For completeness, the data from the entire run are provided in Figures S6 and S7. The comparison between bare 39BB (Figure 2D) and Ti felt (Figure 2E) demonstrates that the GDE substrate material plays a crucial role in facilitating and, consequently, preventing the ORR when O_2_ is present as a contaminant in industrial CO_2_ streams. To validate these findings, we monitored the presence of O_2_ in the reactor outflow with a gas chromatograph (GC), and the results for 3% O_2_ in CO_2_ are presented in Figure 2F. This chromatogram slice shows that less O_2_ was present in the gas outflow when 100 mA cm^*−*2^ was applied for all carbon-based GDEs, demonstrating O_2_ consumption during electrolysis. On the contrary, the Ti felt did not show less O_2_ signal than the situation without current, indicating that the ORR did not occur noticeably with this substrate. These insights open up a new route for preventing the ORR during the CO_2_RR by carefully engineering the GDE and, more specifically, the type of substrate material.

### PTFE filter membrane GDE for CO production

The previous section showed that the GDE substrate material plays a crucial role in preventing the ORR when an O_2_ contaminant is present in the CO_2_ feed stream. In conventional GDEs, the carbon in both the CFS and MPL can aggravate the portion of unwanted ORR, reducing the FE to the desired reaction, although MPL has the greatest impact because of its high surface area. To completely exclude the ORR on the GDL, a non-conductive PTFE filter membrane is used to prevent any electrochemical reaction on this substrate. Our study used an Aspire laminated hydrophobic PTFE filter with 0.2 μm pores, a thickness of 152–254 μm, and a water entry pressure > 45 psi according to the manufacturer’s technical specification sheet.^39^

**Figure 3:**
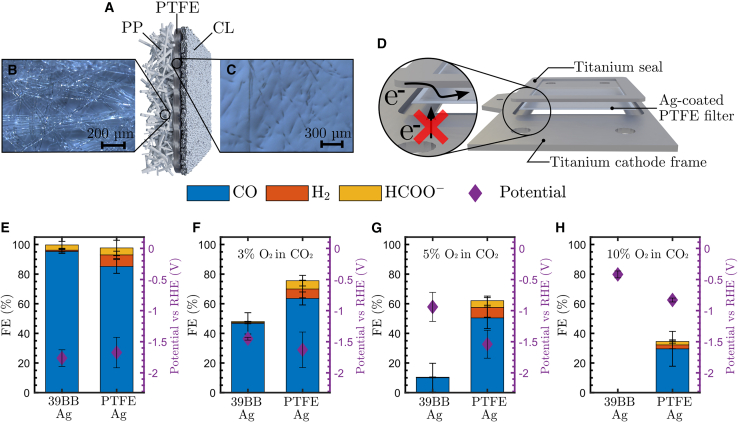
Ag-coated PTFE GDEs for CO_2_ electrolysis in the presence of O_2_ (A) Illustration of the PTFE GDE containing a polypropylene backer, a 0.2 μm porous PTFE filter, and a catalyst layer. (B) Microscopic image of the polypropylene backer. (C) Microscopic image of the PTFE filter layer. (D) Assembly of the PTFE GDE in the cathode frame to provide electrical contact between the catalyst layer and the current collector. (E–H) Ag-coated carbon and PTFE GDEs at 100 mA cm^*−*2^ with 0% (E), 3% (F), 5% (G), and 10% (H) O_2_ in the CO_2_ feed stream. Error bars represent the standard deviation from three measurements.

Figure 3A illustrates the structure of the PTFE GDE. The polypropylene (PP) fibers (Figure 3B) serve as a backing layer for improved rigidity and allow gas transport to the PTFE filter. Similar to carbon-based gas diffusion media, an Ag catalyst is spray coated onto the porous layer to catalyze the CO_2_RR. The 0.2-μm-wide pores of the PTFE filter (Figure 3C) allow gas diffusion to the CL, where the reaction takes place, while blocking the liquid electrolyte from entering the pores through its excellent hydrophobic properties.^40^ A handful of studies have successfully used these kinds of polymer-based gas diffusion media, when coated with copper as the catalyst, for the electroreduction of CO_2_ to multicarbon products.^35^^,^^41^^,^^42^^,^^43^^,^^44^^,^^45^^,^^46^^,^^47^^,^^48^^,^^49^ When tested for over 150 h of electrolysis under a pure CO_2_ feed, these polymer-based GDEs proved to be more stable than carbon-based GDEs.^35^ Moreover, PTFE GDEs have the additional advantages of maintaining short CO_2_ diffusion lengths through liquid and preventing flooding of the electrode, whereas carbon-based GDEs are classified as unsuitable for long-term use in a membrane electrode assembly because of flooding.^47^ Detailed SEM images of the PP and PTFE sides can be found in Figures S8 and S9.

**Figure 4:**
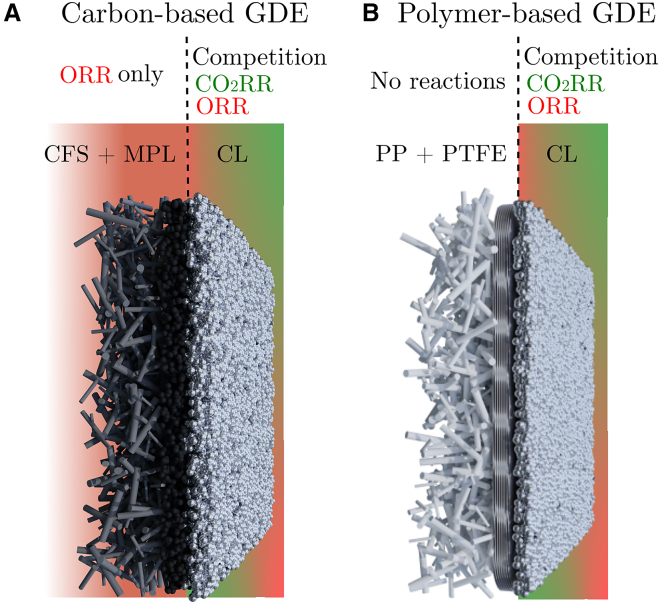
Schematical representation of ORR-active regions in both carbon- and polymer-based GDEs (Left) Carbon GDE. (Right) PTFE GDE.

For further CO_2_ electrolyzer operations, the reactor must be adjusted such that there is electrical contact between the cathode current collector and the CL, which is coated on the non-conductive PTFE substrate. In order to achieve this, we placed a titanium seal on top of the GDE while it was in contact with the cathode frame (Figure 3D) This titanium seal replaced the previously used Viton GDE gasket (part 6 in Figure S10). Results of the slightly modified reactor assembly are shown in Figures 3E–3H. Under a pure CO_2_ feed stream, the carbon-based Sigracet 39BB-Ag GDE performed slightly better (FE_CO_ = 95.3% ± 1.4%) than the PTFE-Ag GDE (FE_CO_ = 85.2% ± 4.6%) (Figure 3E). However, when only 3% O_2_ was present in the gas stream, the PTFE-Ag GDE outperformed the 39BB-Ag with an FE_total_ = 75.6% ± 8.2%. Compared with a >50% FE loss for 39BB-Ag, this demonstrates only a ∼25% FE loss due to the ORR (Figure 3F). Even at 5% O_2_ in the feed, the CO_2_RR remained the dominant reaction, and the ORR accounted for only ∼38% FE, whereas the ORR was responsible for ∼90% FE with 39BB-Ag. When the oxygen concentration increased even further to 10% O_2_ in CO_2_, thereby exceeding the typical concentration in flue gases, the 39BB-Ag failed to produce any CO_2_RR products (Figure 3H). The potential shifted to a less negative value of −1.04 ± 0.05 V (lower overpotential) as a result of the dominant ORR, in contrast to the −1.41 ± 0.03 V observed for the PTFE-Ag, where the system still achieved >30% FE to CO_2_RR products. The main reason why polymer-based GDEs perform better with O_2_ impurities than carbon-based GDEs is the avoidance of the ORR on the substrate and, consequently, the different amount of active sites for the ORR. The use of a polymer-based GDE prevents all ORRs on the substrate because of the non-conductive nature of PP and PTFE, which inherently avoid any electron transfer and thus prevent the ORR from occurring on the substrate. The only region with competition between the CO_2_RR and ORR in polymer-based GDEs is the CL (Figure 4). Furthermore, since the bare carbon-based GDL is capable of facilitating the ORR without contributing to the CO_2_RR (Figure 2D), a carbon-based GDE could contain active sites for the ORR within the carbon-based substrate itself. To investigate this, we carried out electrochemical impedance spectroscopy (EIS) on both the carbon- and polymer-based substrates to determine the double-layer capacitance (C_dl_) and estimate the electrochemically active surface area (EASA) of both configurations (Figure S10). The calculation indicates a greater EASA for the carbon-based GDE, supporting the proposed hypothesis. For completeness, all data for the PTFE-Ag GDE run can be found in Figure S11.

### PTFE-filter-membrane GDE for HCOO^−^ production

**Figure 5:**
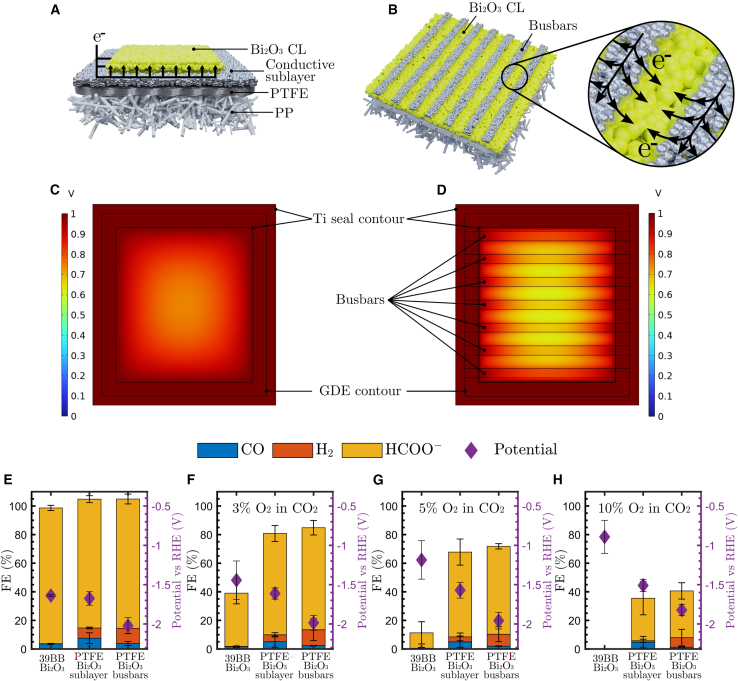
Illustrations and results for the HCOO^*−*^-producing PTFE GDEs (A) Schematic illustration of the PTFE-Bi_2_O_3_ GDE with a conductive sublayer. (B) Schematic illustration of the PTFE-Bi_2_O_3_ with busbars. (C) Potential distribution on the PTFE-Bi_2_O_3_ GDE with a conductive sublayer. (D) Potential distribution on the PTFE-Bi_2_O_3_ GDE with busbars. (E–H) FE for Bi_2_O_3_-coated carbon and PTFE GDEs at 100 mA cm^*−*2^ with (E) 0%, (F) 3%, (G) 5%, and (H) 10% O_2_ in the CO_2_ feed stream. Error bars represent the standard deviation from three measurements.

Using Ag as a catalyst for CO production on polymer-based GDEs has the benefit of being sufficiently conductive by itself, whereas Bi_2_O_3_ is reported to be a highly resistive semiconductor.^50^ As an initial test, we placed a Bi_2_O_3_-coated PTFE GDE in the reactor and set it for a 100 mA cm^*−*2^ run. Two independent measurements showed a FE_HCOO^*−*^_ of only 23.8% ± 11.5% accompanied by high potentials (−5.57 ± 0.60 V vs. reversible hydrogen electrode [RHE]) and unreacted Bi_2_O_3_ areas on the GDE after disassembly as a result of insufficient conductivity (Figure S12). We propose two different GDE configurations to resolve this issue. The first configuration employs a conductive sublayer (1 mg cm^*−*2^ Ag) between the PTFE substrate and the Bi_2_O_3_ CL (2 mg cm^*−*2^), as illustrated in Figure 5A. This conductive sublayer allows for an excellent distribution of electrons to the Bi_2_O_3_ CL but can cause undesired electrochemical side reactions (e.g., producing CO or facilitating the ORR). A second configuration is inspired by solar panels, which use conductive busbars to allow electrons to flow freely from each individual photovoltaic cell.^51^ In this GDE design, 7 × 2-mm-wide conductive lines of Ag are coated on top of the Bi_2_O_3_ CL to provide electron highways from the catholyte-facing side without fully covering the CL (Figure 5B). We modeled the potential distributions of these two GDE configurations in COMSOL Multiphysics (electric-current module) and compared them with the failing base case of a solely Bi_2_O_3_-coated PTFE GDE (more information is provided in the supplemental methods and Figures S13 and S14). The simulations indicated that a conductive sublayer (Figure 5C) or busbar design (Figure 5D) is a potentially valid solution to resolve the lack of conductivity. We compared the resistance of bare Bi_2_O_3_-coated PTFE with that of the proposed designs through EIS measurements (Figure S15) and thus verified the feasibility of both configurations. We aimed to use only conductive materials that also act as catalysts for the CO_2_RR because otherwise, the ORR-active region would increase without increasing the active sites for the CO_2_RR.

Figures 5E–5H compare the performance of the carbon-based 39BB-Bi_2_O_3_ GDE with that of the two PTFE GDE configurations mentioned above (pictures of these prepared GDEs can be found in Figure S16). For the pure CO_2_ feed, both the polymer- and carbon-based GDE substrates gave a high (>85%) FE to HCOO^*−*^, experimentally proving that both the sublayer and the busbar GDE designs are suitable configurations. Surprisingly, although both GDEs contained Ag, the formation of CO remained very limited (FE_CO_ < 8%). A recent report does indeed suggest that Ag-Bi_2_O_3_ electrocatalysts can reach >90% FE_HCOO^*−*^_ at current densities up to 250 mA cm^−2^.^52^ Even when we replaced the Ag sublayer with carbon black (Figure S17), the FEs remained similar to those of the original PTFE-Bi_2_O_3_ GDEs with a Ag sublayer, except that the carbon-black sublayer caused a higher overpotential because its conductivity is lower than that of Ag. Only the potential for the PTFE-Bi_2_O_3_ busbar design (−2.02 ± 0.10 V vs. RHE) differed substantially from the potentials of both 39BB-Bi_2_O_3_ (−1.64 ± 0.01 V vs. RHE) and PTFE-Bi_2_O_3_ with a sublayer (−1.68 ± 0.09 V vs. RHE). An explanation can be found in the conductivity of the different GDEs. Whereas 39BB and the PTFE-Bi_2_O_3_ with a sublayer have bigger surfaces of conductive materials to fully distribute electrons, the busbar design relies more on the conductivity of the Bi_2_O_3_ and suffers from higher electrical resistances, as seen in the simulation of this design (Figure 5D). Apart from the difference in potential, both PTFE-based GDEs achieved similar product selectivities across all measurements (Figures S18 and S19). When the feed contained a mixture of CO_2_ and 3% O_2_ (Figure 5F), the polymer-based GDEs clearly outperformed the carbon-based ones such that the FE_HCOO^*−*^_ remained above 70%, compared with 37.3% ± 7.5% for 39BB-Bi_2_O_3_. This trend continued at 5% O_2_, where the FE_HCOO^*−*^_ was only ∼11% for the 39BB substrate but a remarkable ∼60% for the PTFE GDE designs (Figure 5G). Even with 10% O_2_ in the CO_2_ feed stream, where all FEs for 39BB-Bi_2_O_3_ were attributed to the ORR, PTFE-Bi_2_O_3_ with a sublayer or busbars still reached ∼30% FE to the target product, HCOO^*−*^ (Figure 5H). These results demonstrate that despite the low conductivity of the Bi_2_O_3_ coating, it is possible to engineer a HCOO^*−*^-producing polymer-based GDE, enabling an efficient CO_2_RR in the presence of O_2_ contaminants.

### Influence of the balance gas

When we switched the balance gas from CO_2_ to N_2_ to examine the effect of different O_2_ concentrations in another medium (Figures S20–S26), all GDE types (39BB-Ag, 39BB-Bi_2_O_3_, bare 39BB, bare Ti, PTFE-Ag, PTFE-Bi_2_O_3_ with sublayer, and PTFE-Bi_2_O_3_ with busbars) showed FE values similar to those under their CO_2_ equivalent conditions. However, a different potential was naturally observed because the CO_2_RR cannot occur when the feed stream consists of N_2_, and therefore, the HER is the main reaction. The fact that the trends and total FEs were similar between CO_2_ and N_2_ allows us to conclude that the ORR in this work does not depend notably on the type of balance gas used.

### Stability assessment and industrially relevant conditions

**Figure 6:**
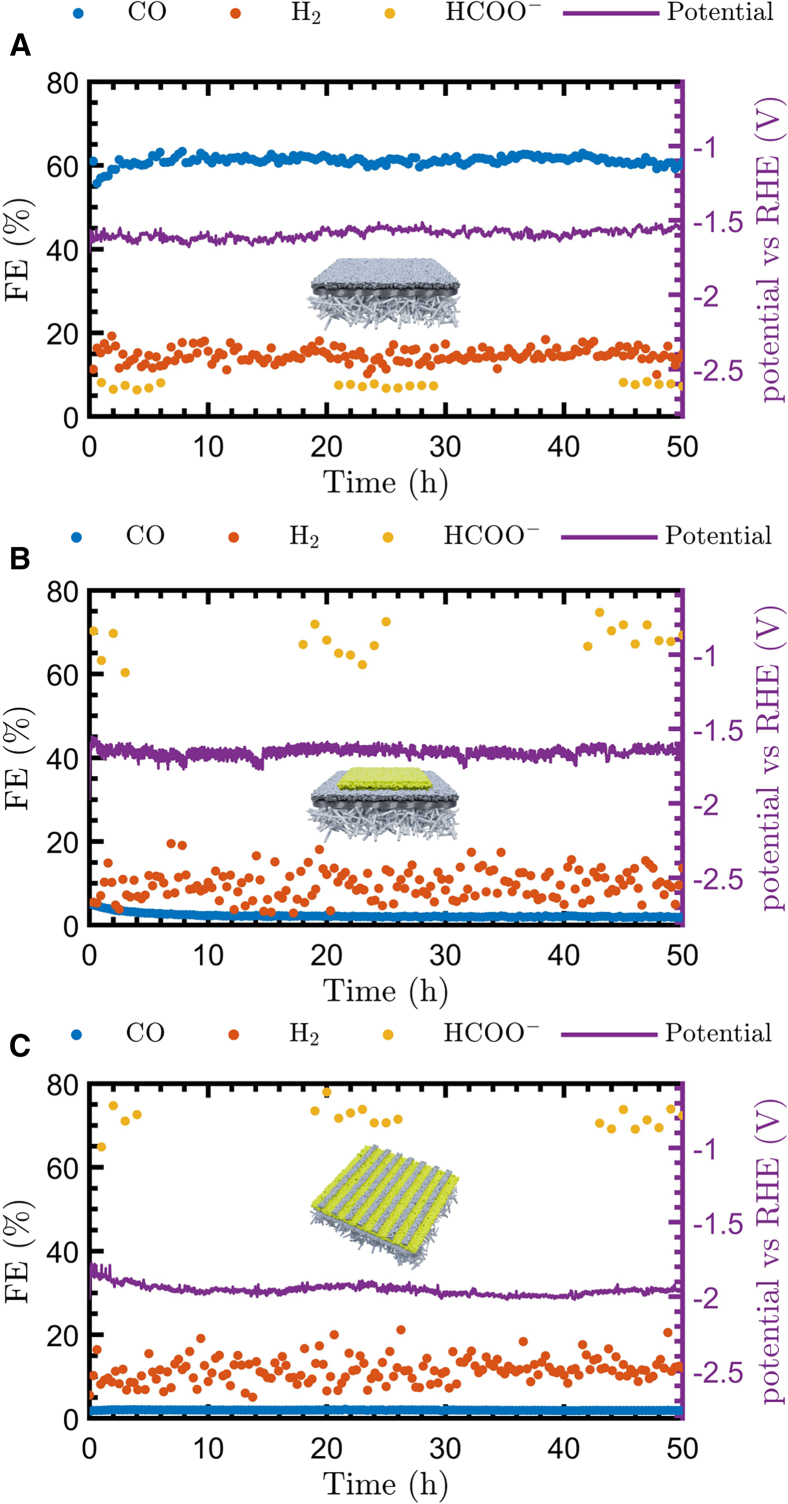
Stability measurements of 50 h with 3% O_2_ in the CO_2_ feed stream at 100 mA cm^*−*2^ for the polymer-based GDEs (A) Results of the PTFE-Ag GDE. (B) Results of the PTFE-Bi_2_O_3_ GDE with a conductive sublayer. (C) Results of the PTFE-Bi_2_O_3_ GDE with busbars.

The advantage of using polymer-based GDEs for CO_2_RR application from flue gas sources raises the question of whether these types of GDEs are suitable for long-term CO_2_ electrolyzer operation in the presence of O_2_ contaminants. To assess stability, we subjected each polymer-based GDE to a 50 h measurement at 100 mA cm^*−*2^ with 3% O_2_ in the CO_2_ feed stream. As stated in the methods section, we fixed the total gas flow rates at 100 mL min^*−*1^ and pumped electrolytes at 5 mL min^*−*1^ to the electrolyzer in single-pass mode to ensure the most stable reactor operating conditions. The results of the stability measurements are shown in Figure 6. For the PTFE-Ag GDE, Figure 6A reveals that over the course of 50 h, an average FE_CO_ of 61.13% was achieved at a potential of −1.59 V vs. RHE without any notable sort of degradation. Inductively coupled plasma mass spectrometry (ICP-MS) analysis of the catholyte outflow at the end of the measurement (Table S15) did not detect any Ag, indicating that the coating remained attached to the substrate. Secondly, the PTFE-Bi_2_O_3_ with a conductive sublayer (Figure 6B) showed a steady potential at an average value of −1.66 V vs. RHE but a FE_HCOO^*−*^_ > 60% for the entire measurement. Lastly, relative to the aforementioned designs, the PTFE-Bi_2_O_3_ busbar design (Figure 6C) showed an outstanding average FE_HCOO^*−*^_ of 71.77% despite the 3% O_2_ in the feed but at the cost of a higher potential (−1.96 V vs. RHE) due to the increased resistance from the design. It is worth mentioning that, after disassembly, the PP side facing the gas chamber of the PTFE-Bi_2_O_3_ busbar design exhibited specific rows with water droplets (Figure S27). These rows align with the busbars on the catholyte side, which indicates that electrolyte seepage through the GDE, known as perspiration,^53^ occurred for the majority through these busbars. This observation leads to the hypothesis that perspiration is now guided through the highly conductive busbars as a result of the electrowetting effect, creating specific zones for salt removal while leaving the catalytic Bi_2_O_3_ zones dry and open for efficient CO_2_ gas diffusion. Furthermore, ICP-MS results indicated extremely limited detachment of Bi_2_O_3_ particles in all measurements (<2 × 10^*−*2^ ppm). Consequently, all polymer-based GDEs were assessed as stable, confirming the excellent stability of previously reported PTFE GDEs with pure-CO_2_-fed electrolyzers.^35^^,^^47^

Previous work with solely carbon-based GDEs has shown that the FE losses by the ORR become lower when the electrolyzer operates at >100 mA/cm^2^ given that the O_2_ supply to the electrode becomes mass transport limited.^26^ Therefore, we conducted experiments up to 300 mA/cm^2^ with a 3% O_2_ feed stream, and the results for the three GDE configurations are shown in Figure S28. In line with previous work, the total FE increased at higher CD because the O_2_ supply was mass transport limited, giving room for the desired CO_2_RR until the parasitic H_2_ evolution reaction broke through as a result of the higher operating potential and lowered CO_2_ availability (more consumption) near the electrode surface. Figure S29 reveals the PTFE-Bi_2_O_3_ sublayer as the best configuration in terms of productivity because it reached a partial CD to C-products of 220 ± 25.0 mA/cm^2^ when a total CD of 300 mA/cm^2^ was applied. In terms of activity, the PTFE-Ag GDE held the lowest potentials across all the different polymer-based GDE configurations. Lastly, the polymer-based GDEs were tested with a simulated flue gas (15% CO_2_ + 4% O_2_ in N_2_), in agreement with other literature reports.^20^^,^^46^^,^^54^ Because of the low (15%) CO_2_ content, the GDEs were tested at current densities of 50–150 mA/cm^2^ given that a CO_2_ shortage at the electrode prevents efficient operation. In fact, Figure S30 shows that the combination of diluted CO_2_ with O_2_ poses great challenges to maintaining good product selectivity. Nevertheless, FE losses of the ORR remained similar to those of the concentrated CO_2_ feed stream with O_2_; therefore, the O_2_ tolerance of the polymer-based GDEs remained unaffected, yet future work should focus on enhancing CO_2_ mass transport to the active sites with simulated flue gas feed streams. From all polymer-based GDE configurations, the PTFE-Bi_2_O_3_ with a conductive sublayer performed best in terms of productivity and beat the activity of the PTFE-Ag for potentials lower than −1.82 V vs. RHE (Figure S31). However, the partial CD for the CO_2_RR products remained lower than 30 mA/cm^2^ and should be a priority for improvement in future work.

### Conclusions

Polymer-based GDEs offer a promising alternative to carbon-based GDEs, especially when O_2_ is present as a contaminant in CO_2_ feed streams. This study shows that the GDE substrate plays a major role in FE losses when O_2_-containing CO_2_ feed streams are supplied to the electrolyzer. Conventional carbon-based GDEs can facilitate the ORR even when no metal catalyst is coated on the substrate, resulting in a total product FE < 20% for feed streams containing 5% O_2_. The transition to a non-conductive polymer-based GDE improves the FE to CO_2_RR products (CO or HCOO^*−*^) by >40% (5% O_2_ in CO_2_). Importantly, all three polymer-based GDE configurations show a stable performance over the course of 50 h, thus confirming their potential usage in long-term electrolyzer operation. Furthermore, increasing the productivity for the CO_2_RR to CO or formate from a low concentrated CO_2_ feed containing O_2_ impurities poses a great challenge to the direct utilization of waste CO_2_ streams. The insights from this study can be used in the design of a CO_2_ electrolysis process that uses impure CO_2_ sources to create valuable chemicals. Omitting or reducing the necessary separation and purification steps is of tremendous interest because it would make CO_2_ electrolyzer technologies more economically viable.

## Methods

### Chemicals and products

We made the electrolytes by dissolving potassium bicarbonate (>99.5%, Chem-lab) or potassium hydroxide (>85%, Chem-lab) in ultrapure water (Milli-Q, Millipore; resistivity: 18.2 MΩ cm) to obtain the desired concentration of reagents (0.5 M KHCO_3_ and 1 M KOH). Perchloric acid (70%, Chem-lab) was diluted with ultrapure water to a 1.2 M solution and used during high-performance liquid chromatography (HPLC) sample preparation. We used sulfuric acid (>98%, Chem-lab) to prepare the 10 mM H_2_SO_4_ mobile phase for HPLC analysis and used nitric acid (70%, Chem-lab) to prepare samples for ICP-MS. Hydrogen peroxide (30%, VWR) was used during activation of the Nafion 117 cation-exchange membrane (Ion Power). We used commercial bismuth oxide nanoparticles (99.8%, 90–210 nm, Sigma-Aldrich), silver nanoparticles (99.5%, <100 nm, Sigma-Aldrich), or carbon black (Vulcan XC72, Nanografi) with isopropanol (99.8%, Chem-lab), ultrapure water, and a Nafion dispersion (D520, 5% w/w in water, and 1-propanol, Thermo Fisher Scientific) to prepare the catalyst inks. Transmission electron microscopy images and X-ray diffraction measurements of the Ag and Bi_2_O_3_ catalytic nanoparticles can be found in Figures S32 and S33. Ethanol (>99.9%, Chem-lab), acetone (>99.8%, Chem-lab), and a 60% (w/w) PTFE dispersion in water (Sigma-Aldrich) were used during the sintering procedure. Helium (99.999%, Air Liquide) was used as carrier gas for the GC, and argon (99.999%, Air Liquide) was used for spray coating. CO_2_ (99.998%, Air Liquide), an O_2_/CO_2_ mixture (19.96% O_2_ in CO_2_, Nippon gases), a simulated flue gas (15% CO_2_ + 4% O_2_ in N_2_, Air Liquide), and air (20% ± 1% O_2_ in N_2_, Air Liquide) were used during experiments.

### GDEs

We made the catalyst ink solutions by mixing 72 mg of nanoparticles (Ag or Bi_2_O_3_) with 200 mg (5% w/w) Nafion dispersion, isopropanol, and ultrapure water unless stated otherwise. The volumetric isopropanol/water ratios were 4:1 and 2:1 for Bi_2_O_3_ and Ag, respectively, for a total volume of 6 mL. Inks were sonicated for at least 30 min with a 6 mm titanium probe (NexT-gen Lab120 at 34 kHz with an 84 μm amplitude). Using a Fengda FE-186K airbrush with argon as the carrier gas, we spray coated the inks onto the GDLs, which were fixed on a hot plate (80°C). We used two main GDLs as the substrates for the catalyst inks: the carbon-based Sigracet GDL 39BB (purchased from Ion Power) and the polymer-based Aspire laminated hydrophobic PTFE filter with 0.2 μm pores (purchased from Sterlitech), each with a geometrical active surface area of 10 cm^2^. Their specific properties are listed in Tables S16 and S17, respectively. We weighed the GDEs before and after spray coating to ensure a catalyst loading of 2 mg cm^*−*2^. In the PTFE-Bi_2_O_3_ configuration with a conductive sublayer or busbar design, we spray coated the second CL by using a PMMA mask with a smaller rectangle than the original layer or a busbar-shaped pattern. For experiments mentioning Ti felt, we used a 50%–56% porosity Ti fiber felt (purchased from Fuel Cell Store) after pretreating it to increase its hydrophobic properties such that it was able to maintain a gas-liquid boundary during operation. The pretreatment was adapted from the methods of Omrani and Shabani^55^ and consisted of (1) cleaning the Ti felt with acetone, ethanol, and distilled water; (2) drying it at 120°C for 30 min; (3) dipping it in a 25% (w/w) PTFE emulsion; (4) drying it at 120°C for 60 min; and (5) sintering it at 340°C under argon atmosphere for 60 min.

### Flow-reactor operation

Similar to previous reports,^21^^,^^56^^,^^57^ this study used a modified ElectroCell Micro Flow Cell (Figure S34) with a Ag/AgCl reference electrode (Innovative Instruments). Viton gaskets and PMMA flow plates were fabricated in house with a CNC mill (Euromod MP45). The Nafion 117 cation-exchange membrane separated the catholyte and anolyte compartments. To enhance its ionic conductivity and remove organic contaminants, we pretreated the membrane by sequentially boiling it in the following solutions: 3% H_2_O_2_ (1 h), distilled water (1 h), 1 M H_2_SO_4_ (1 h), and distilled water again (1 h). After pretreatment, the membrane was stored at 4°C until further use.

In the experimental setup, a peristaltic pump (Shenchen LabS3, Drifton) supplied the cathode compartment with 5 mL min^*−*1^ 0.5 M KHCO_3_. On the anode side, the pump supplied a 1 M KOH solution with the same flow rate to facilitate the oxygen evolution reaction. Both electrolytes were pumped through the cell in single-pass mode, ensuring stable and reliable conditions during the experiments. The gas feed stream was controlled with Analyt-MTC mass flow controllers. We obtained different feed-gas compositions by mixing 20% O_2_ in CO_2_ with pure CO_2_ in different volumetric ratios. We conducted electrochemical measurements with a potentiostat (PGSTAT302N, Metrohm) and a 10 A booster (Metrohm) and non-iR corrected the reported working-electrode potentials. The potentials were converted to RHE through the equation $E_{\text{RHE}}=E_{\text{Ag}/\text{AgCl}}+E_{\text{Ag}/\text{AgCl}}^{0}+0.058\times \text{pH}$, where $E_{\text{Ag}/\text{AgCl}}^{0}$ = 0.197 V and pH = 8.36. Typically, we operated the reactor for 25 min at a given condition, took a liquid sample from the catholyte outflow, and then injected a gas sample into the in-line GC before switching to the next operating condition. We conducted EIS by using the potentiostat’s frequency response analyzer (FRA) module to obtain the C_dl_ and estimate the EASA as $\text{EASA}=\frac{\text{Cdl}}{C_{s}}$ by using a specific charge density of C_s_ = 40 μF/cm^2^.^58^

### Product analysis and imaging

We acidified 1 mL of liquid catholyte sample with 1 mL of 1.2 M HClO_4_. After acidification, we vortexed the samples and filtered them with a 0.2 μm filter to prepare them for analysis. The analysis was performed on an HPLC system (Alliance 2695) equipped with a Shodex RSpak KC811 column and a PDA detector (210 nm, Waters). We injected the acidified and filtered samples into the column and compared them with a 1,000 ppm standard to quantify the amount of HCOO^*−*^ produced during reactor operation. For accurate calculation of FE, we determined the real liquid flow rate by monitoring the weight of the catholyte reservoir. After the 50 h stability measurements, we diluted a catholyte sample in 1% nitric acid and measured it with ICP-MS (Agilent 7500 Series).

The gaseous reactor outlet was directly connected to a GC (Shimadzu) equipped with a Restek Shincarbon ST column (1 mm internal diameter, 2 m length, and mesh 100/120) for product analysis and helium as carrier gas. The product analysis began at a temperature of 40°C and continued for 3 min. After that, the temperature increased linearly at a rate of 40°C per minute until it reached 250°C. The thermal conductivity detector of the GC remained at a constant temperature of 280°C throughout the analysis. For precise product quantification, the outgoing gas flow rate was measured with a volumetric flow meter (Restek ProFlow 6000).

Microscopic GDE images were obtained with an M165 C light microscope (Leica Microsystems). SEM images were performed on the GDE samples with a Thermo Fischer Scientific Quanta FEI 250 microscope operated at an accelerating voltage of 20 kV.

## Resource availability

### Lead contact

Requests for further information and resources should be directed to and will be fulfilled by the lead contact, Tom Breugelmans (tom.breugelmans@uantwerpen.be).

### Materials availability

This study did not generate new unique materials.

### Data and code availability

• The (processed) data are freely available from the Zenodo repository of the University of Antwerp and Applied Electrochemistry and Catalysis (ELCAT) Research Group: https://zenodo.org/communities/uantwerp-elcat/.
• This paper does not report original code.
• Any additional information required to reanalyze the data reported in this paper is available from the lead contact upon request.

## Acknowledgments

This work was funded by the 10.13039/501100000780European Union for actions 101088063 - TRANSCEND and 101092257 - THREADING-CO2. The views and opinions expressed are, however, those of the authors only and do not necessarily reflect those of the European Union or the European Research Council Executive Agency (ERCEA). Neither the European Union nor the ERCEA can be held responsible for them. This project was co-funded by the Flanders Industry Innovation Moonshot program via grant CAPTIN II HBC.2021.0255. The authors thank Max Van Brusselen for performing the ICP-MS measurements, as well as Kavita Shivanagoud Patil and Brend De Coen for acquiring the SEM images.

## Author contributions

Conceptualization, S.V.D., L.H., and D.C.; investigation, S.V.D. and L.H.; visualization, S.V.D.; writing – original draft, S.V.D.; writing – review & editing, L.H., D.C., and T.B.; supervision, D.C., N.D., J.H., and T.B.; project administration, N.D., J.H., and T.B.; funding acquisition, N.D., J.H., and T.B.

## Declaration of interests

The authors declare no competing interests.

## Supplemental information

Document S1. Figures S1–S34, Tables S1–S17, and supplemental methods

Data S1. *Chem Catalysis* checklist

Document S2. Article plus supplemental information
