# Supplementary material for: Promoting CO2 reduction in the presence of oxygen with polymer-based gas diffusion electrodes
Source: Chem Catal. 2025 Jul 17;5(7):101353. doi: 10.1016/j.checat.2025.101353 (PMC12271024; doi:10.1016/j.checat.2025.101353)
Supplement: Document S1. Figures S1–S34, Tables S1–S17, and supplemental methods [file mmc1.pdf]

Chem Catalysis, Volume *5*

Supplemental information

Promoting CO_2 reduction in the presence of oxygen with polymer-based gas diffusion electrodes

Sam Van Daele, Lieven Hintjens, Daniel Choukroun, Nick Daems, Jonas Hereijgers, and Tom Breugelmans

Supplemental Methods

The potential distribution in the GDE was modelled in COMSOL Multiphysics (Electric Currents module) with the following simplifications:

- PP and PTFE are considered perfect insulators and therefore not included in the model.
- Catalyst layers are modelled as a bulk material.
- Joule heating and general heat transfer are considered negligible.

Since modelling of the exact morphology and conductivity of spray coated ionomer-containing catalyst ink layers is extremely complex, these materials are assigned a lower conductivity than their bulk metallic value in order to approach the characteristics of the catalyst layer.

The base case consists of a 5 µm thick Bi_2O_3 CL ($\sigma = 10^3$ S m^{-1}) in contact with a 1 mm Ti frame ($\sigma = 7{\cdot}10^5$ S m^{-1}). The electrolyte is represented by a 1 cm thick rectangle ($\sigma = 10^{-1}$ S m^{-1} for a 0.5 M salt solution) that is in contact with the CL. A potential of 1 V is applied on the Ti frame as illustrated in Fig. S.13.

Other configurations are modelled in exactly the same way as the base case, but only the CL thickness and materials are changed. This includes a PTFE GDE with a 5 µm thick Ag CL ($\sigma = 10^3$ S m^{-1}) (Fig. S.14 A), a 2 µm thick Ag sublayer with a 5 µm Bi_2O_3 CL (Fig. S.14 B) and a 5 µm Bi_2O_3 CL with 7x2 cm thick Ag busbars included.

Supplemental Figures

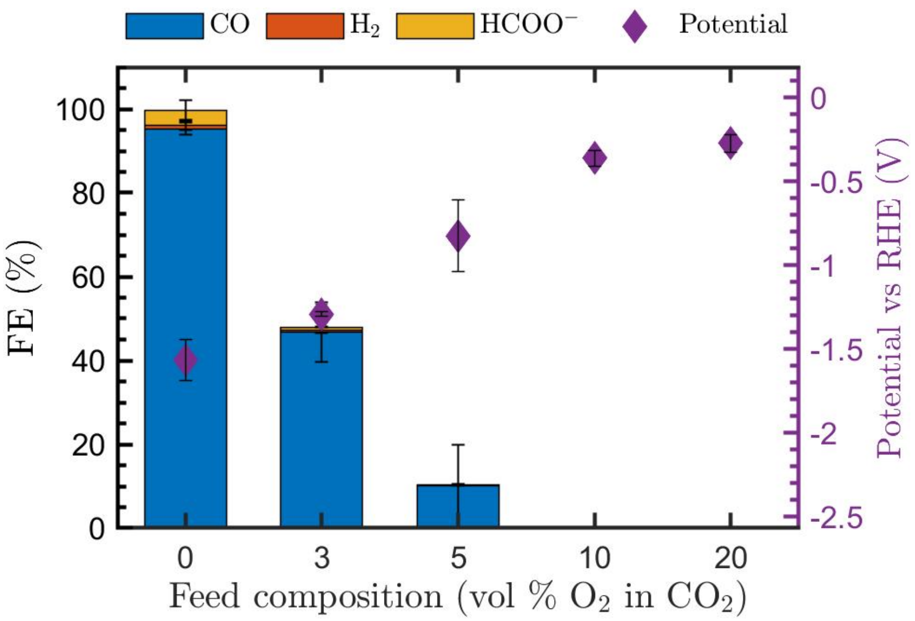

Figure S.1: All results for the carbon-based 39BB GDE with Ag nanoparticles at 100 mA cm^{-2}, related to Fig. 2.

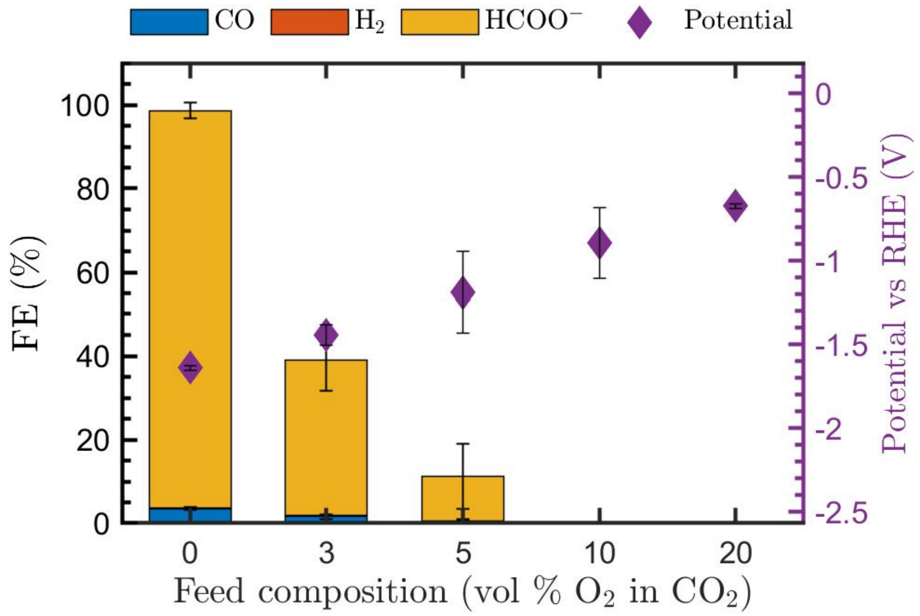

Figure S.2: All results for the carbon-based 39BB GDE with Bi_2O_3 nanoparticles at 100 mA cm^{-2}, related to Fig. 2.

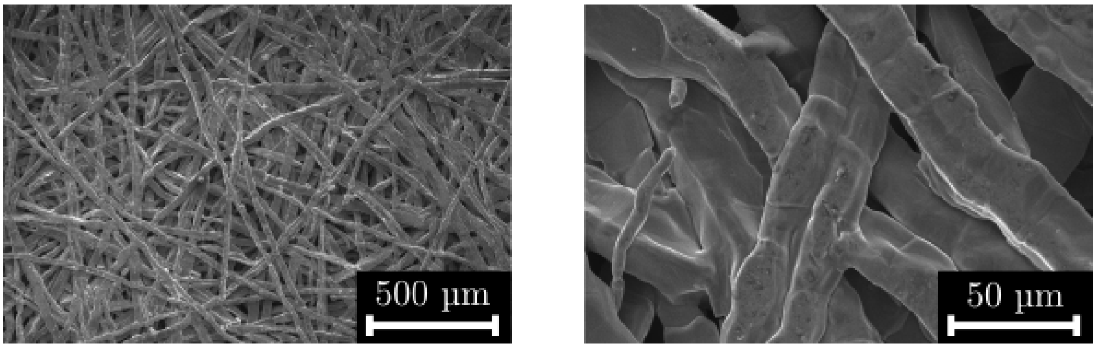

Figure S.3: SEM images of bare Ti felt at different magnifications.

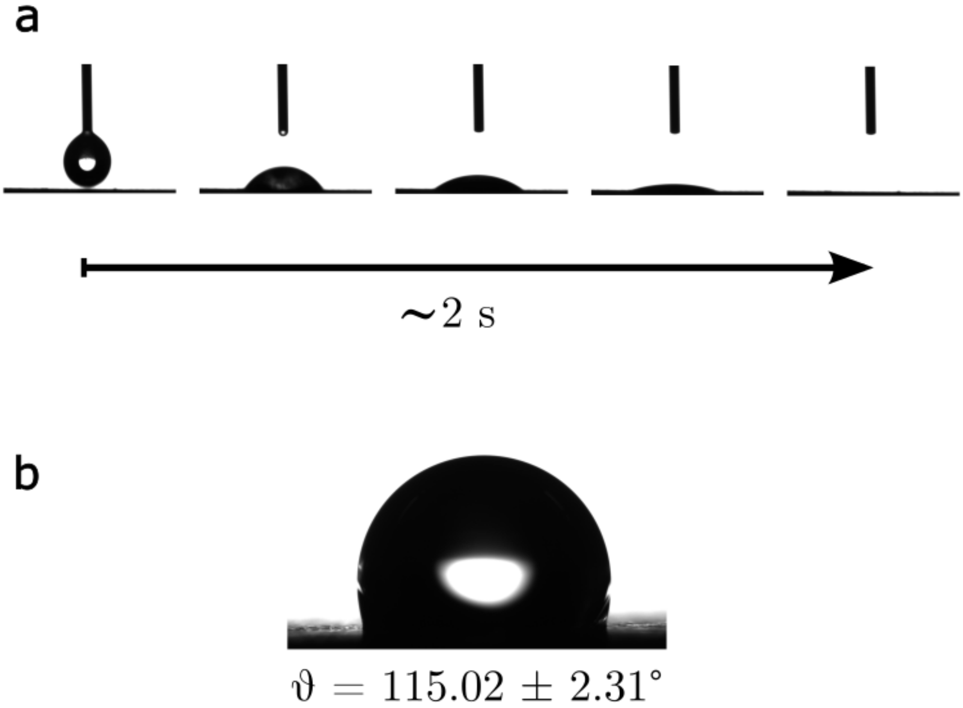

Figure S.4: Contact angle measurements on bare Ti felt. **a** Measurement of untreated Ti felt. The insufficient hydrophobicity will not provide a gas-liquid barrier in the reactor. **b** Contact angle of Ti felt after a sintering treatment with PTFE.

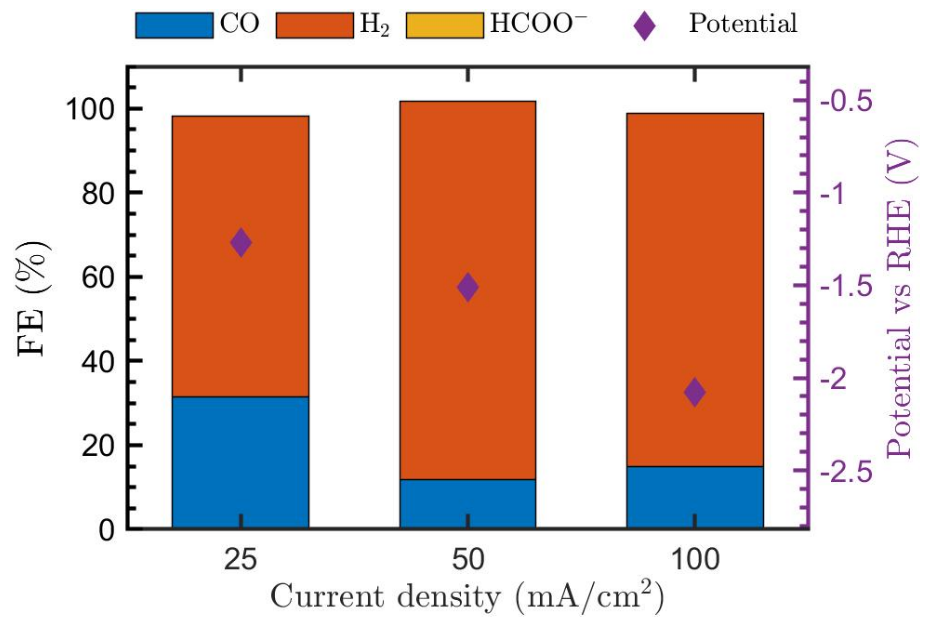

Figure S.5: Faradaic efficiency for a Ag-coated Ti felt. The feed stream consisted of 100 mL/min pure CO_2.

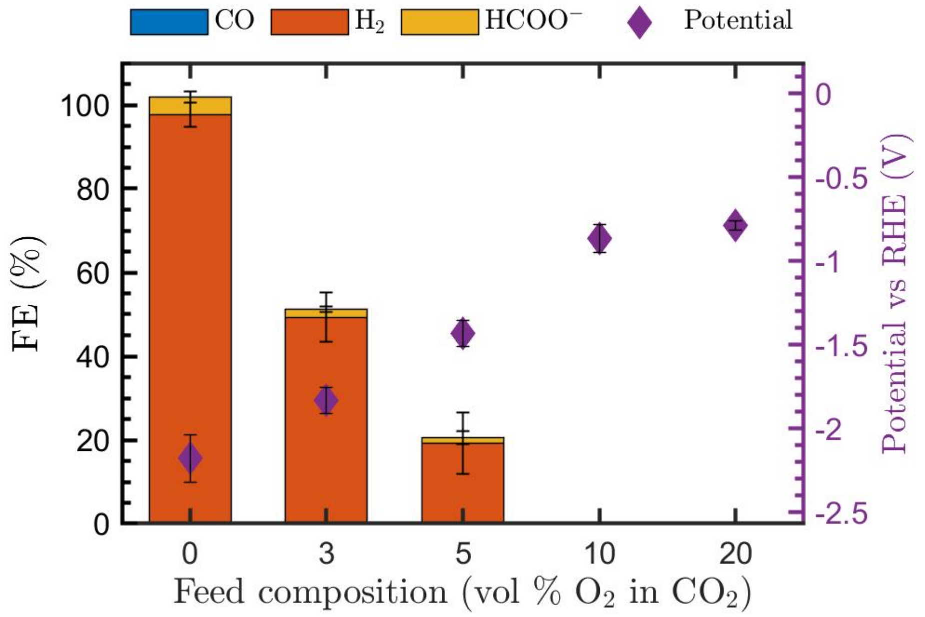

Figure S.6: All results for the bare 39BB carbon-based GDL at 100 mA cm^{-2}, related to Fig. 2.

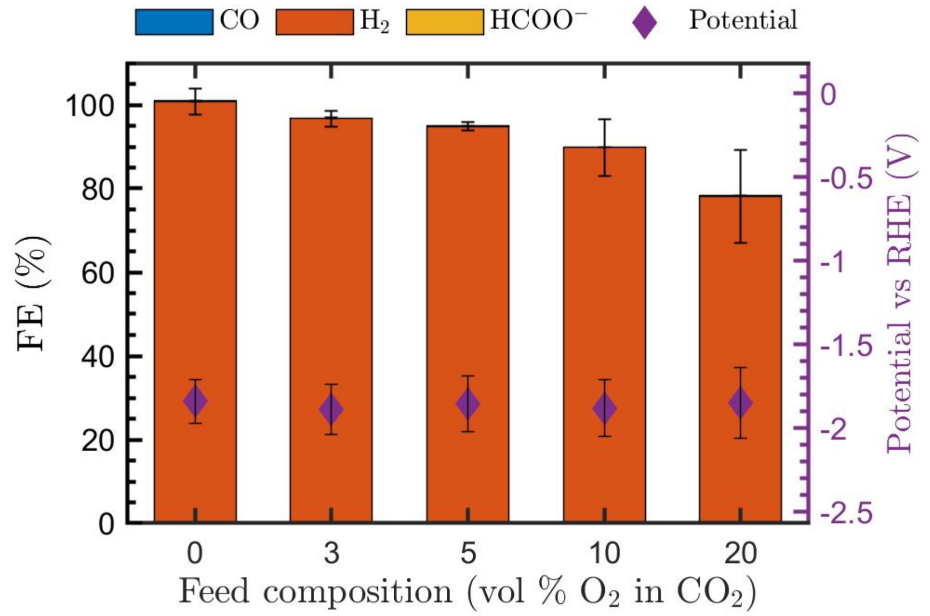

Figure S.7: All results for the bare Ti felt at 100 mA cm^{-2}, related to Fig. 2.

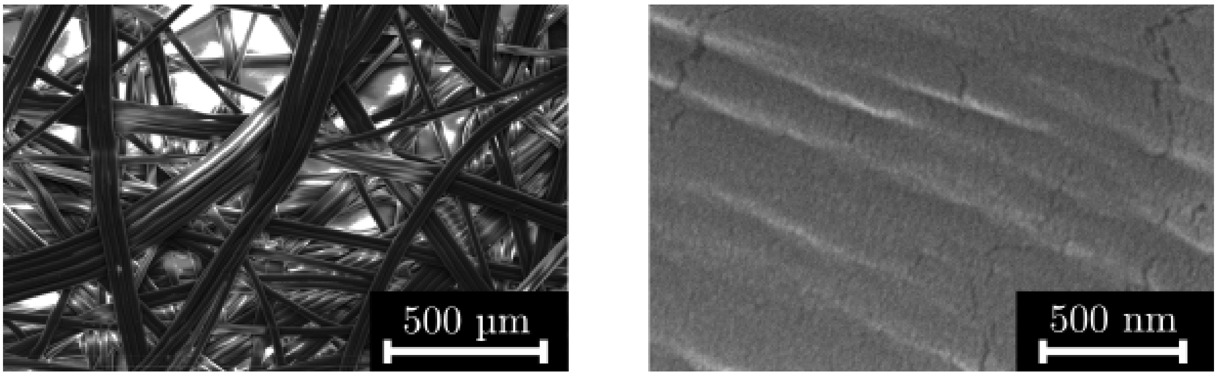

Figure S.8: SEM images of the PP backer at different magnitudes. A thin layer of 25 nm Ag is sputtered on the sample to avoid charging during imaging.

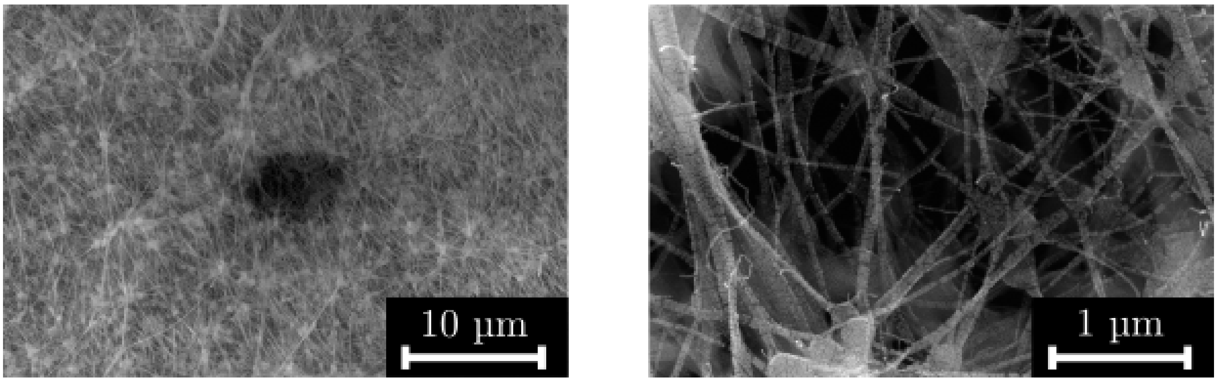

Figure S.9: SEM images of the PTFE side at different magnitudes. A thin layer of 25 nm Ag is sputtered on the sample to avoid charging during imaging.

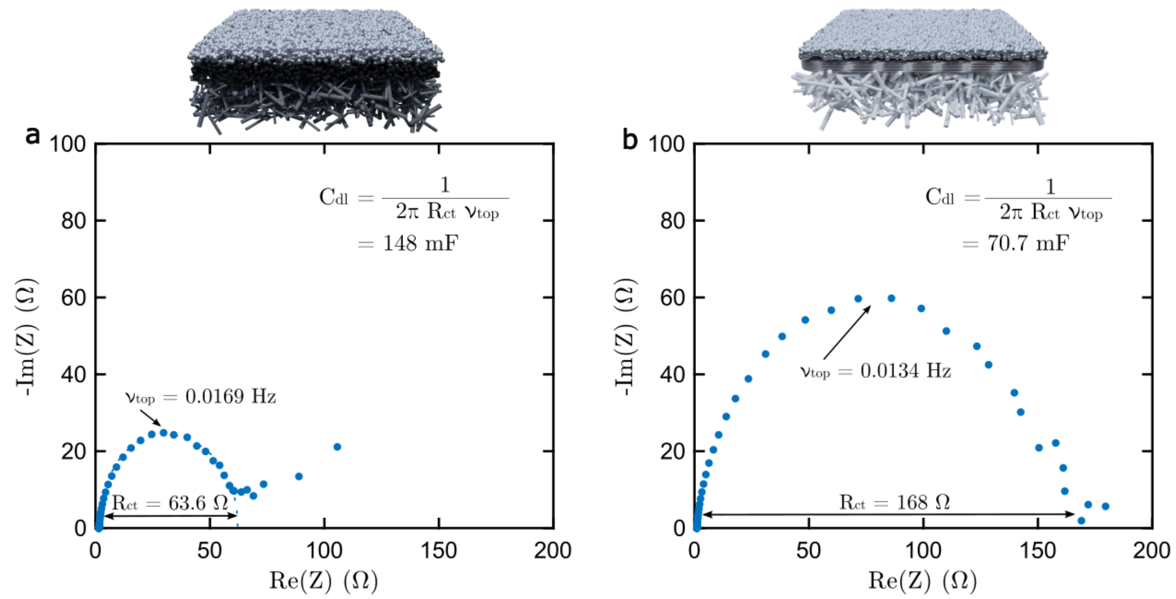

Figure S.10: Electrochemical impedance spectroscopy on the 39BB-Ag and PTFE-Ag GDE to estimate EASA $= \frac{C_{dl}}{C_s}$ with $C_s = 40\ \mu F/cm^2$. **a** 39BB-Ag with an EASA of $3.70 \cdot 10^3\ cm^2$. **b** PTFE-Ag with an EASA of $1.77 \cdot 10^3\ cm^2$.

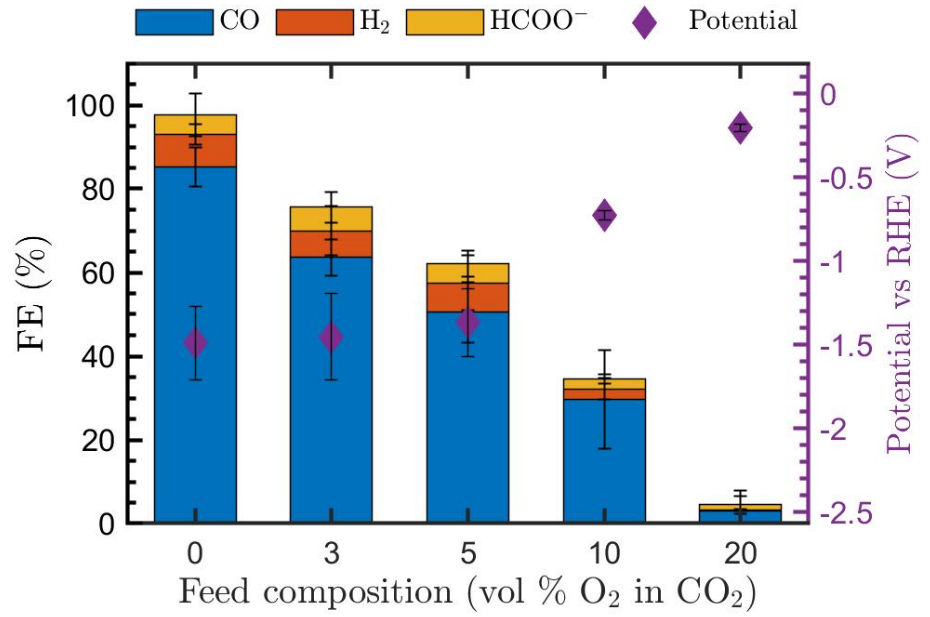

Figure S.11: All results for the Ag-coated PTFE substrate at 100 mA cm^{-2}, related to Fig. 3.

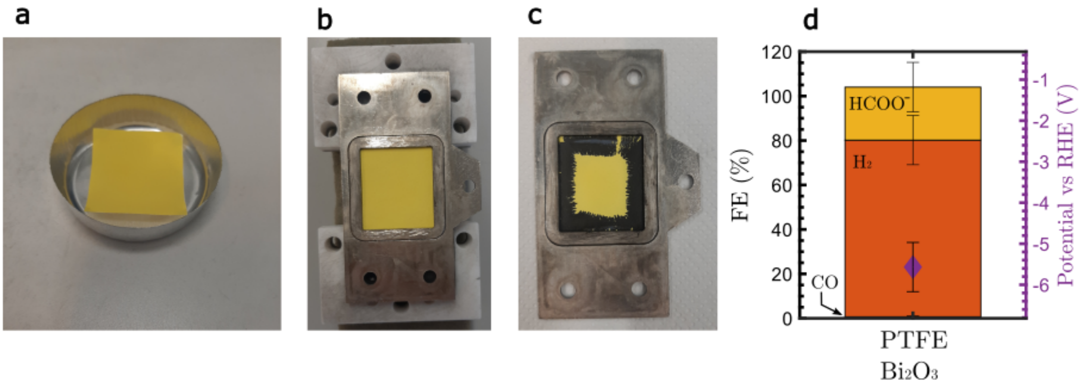

Figure S.12: Pictures and reactor operation with a Bi_2O_3-coated PTFE GDE. **a** Picture of the prepared GDE. **b** Picture of the Cathode frame with the prepared GDE. **c** Unreacted (yellow) and reacted (dark blue/black) areas after reactor operation. **d** Results derived from two independent reactor runs.

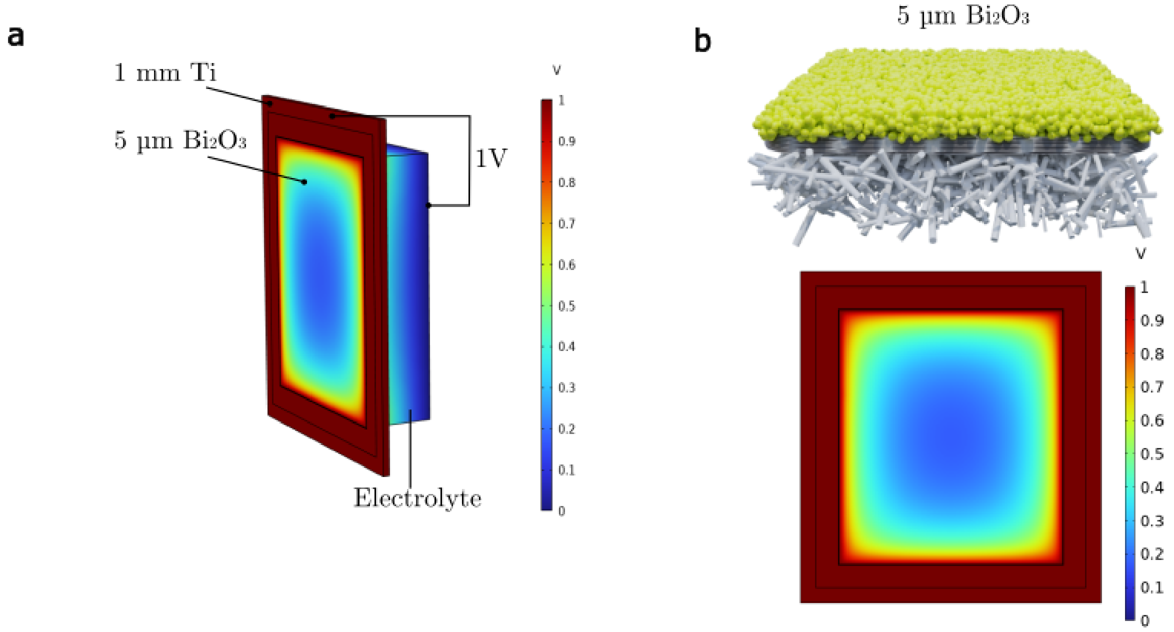

Figure S.13: Potential distribution in the CL of the PTFE-Bi_2O_3 base case. **a** Schematical representation of the model. **b** Potential distribution in the CL plane on the intersection between electrolyte and CL.

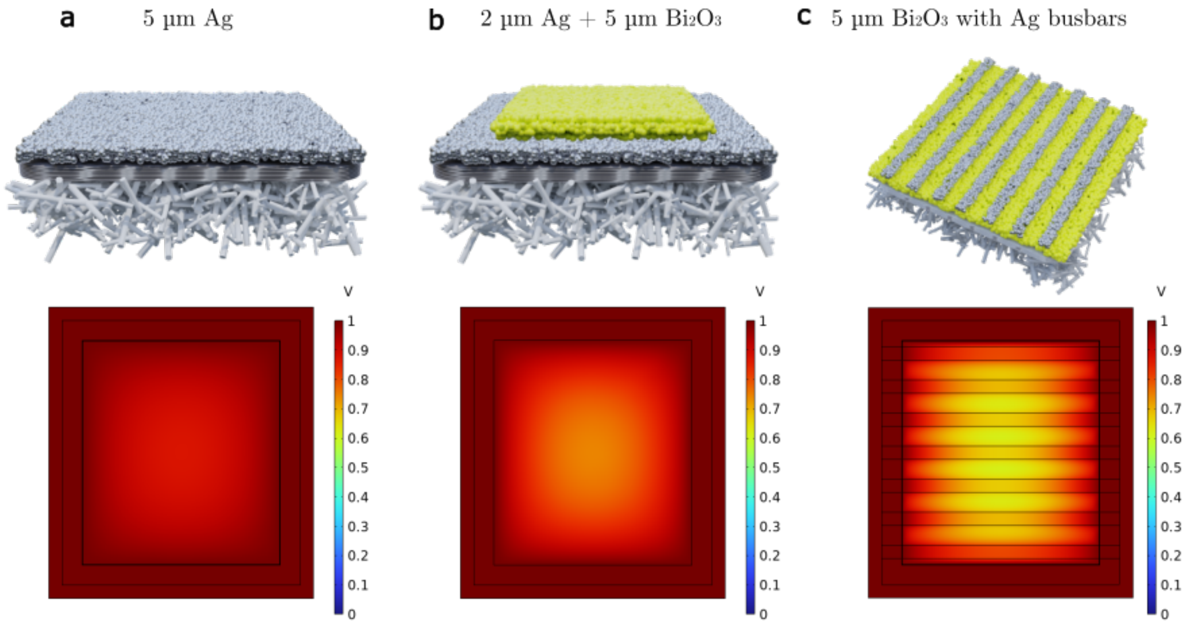

Figure S.14: Potential distribution in the CL for different PTFE GDE configurations. **a** PTFE-Ag. **b** PTFE Bi_2O_3 with a conductive Ag sublayer. **c** PTFE Bi_2O_3 with Ag busbars.

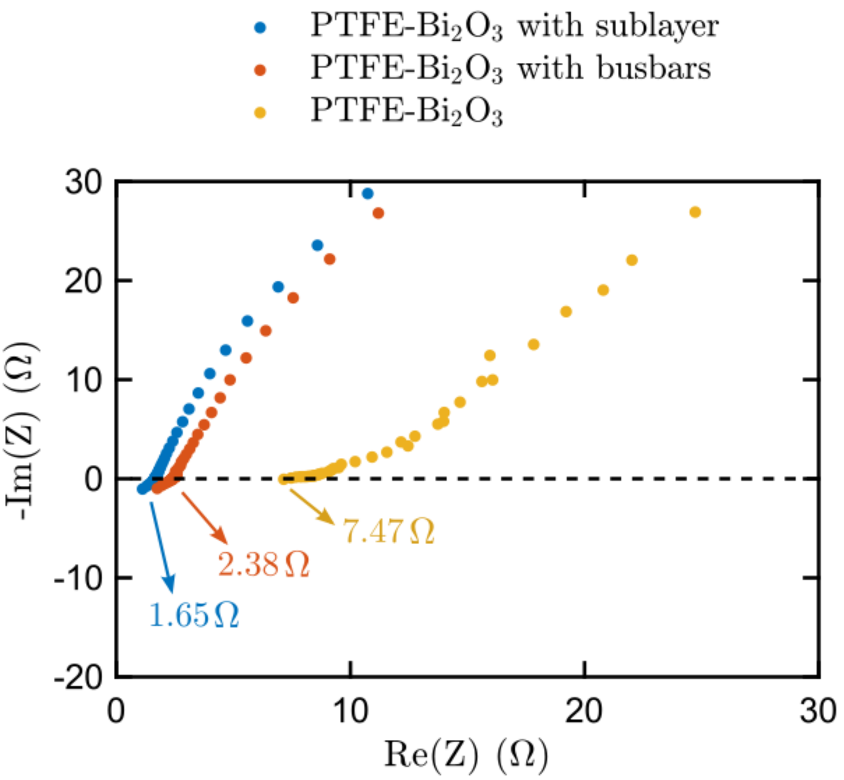

Figure S.15: Comparison of resistance between the two proposed polymer-based GDE designs and the solely Bi_2O_3-coated PTFE GDE through electrochemical impedance spectroscopy.

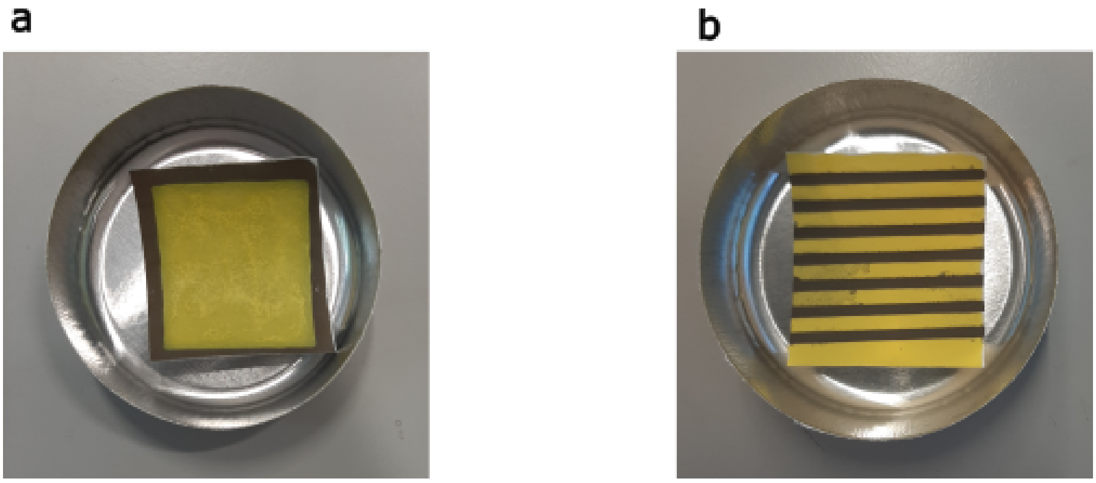

Figure S.16: Pictures of the PTFE-Bi_2O_3 configurations. **a** PTFE-Bi_2O_3 with a conductive Ag sublayer. **b** PTFE-Bi_2O_3 with 7x2 mm Ag busbars.

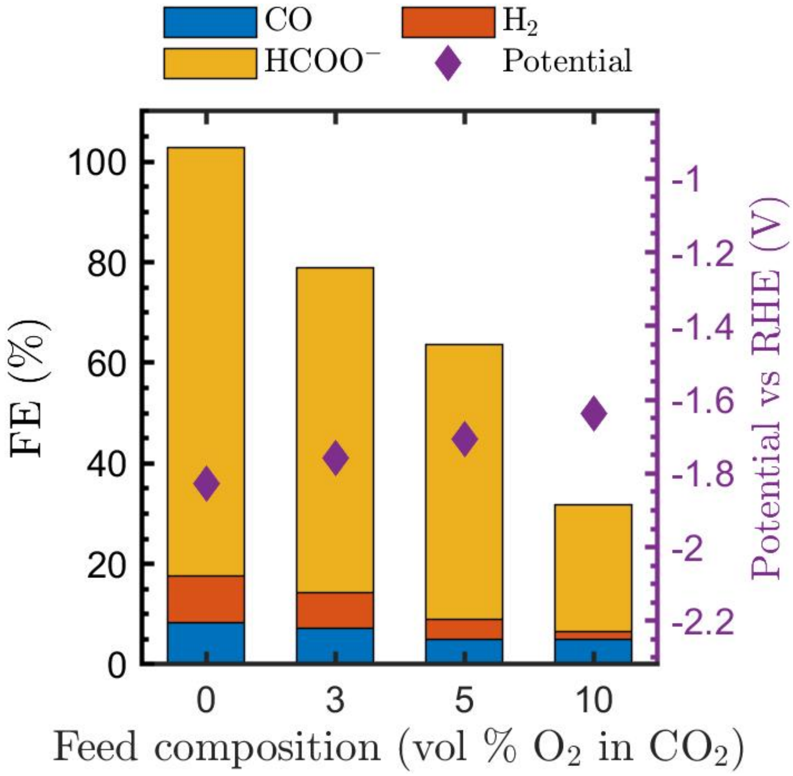

Figure S.17: Experimental results for a PTFE GDE with 1 mg/cm^2 carbon black sublayer with a 2 mg/cm^2 Bi_2O_3 top layer for different oxygen concentrations at 100 mA/cm^2 and 100 mL/min.

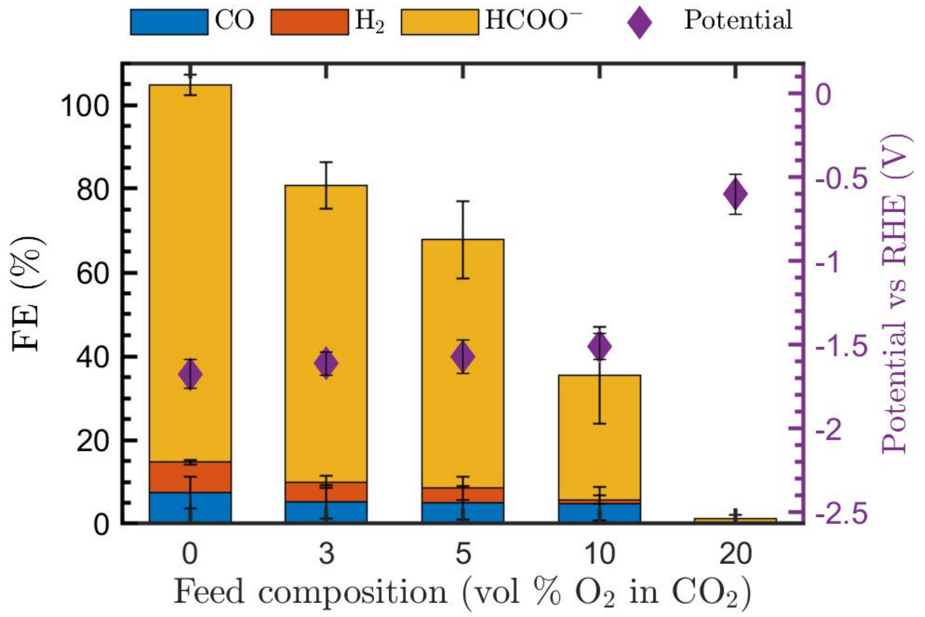

Figure S.18: All results for the PTFE-Bi_2O_3 GDE with a conductive sublayer at 100 mA cm^{-2}, related to Fig. 5.

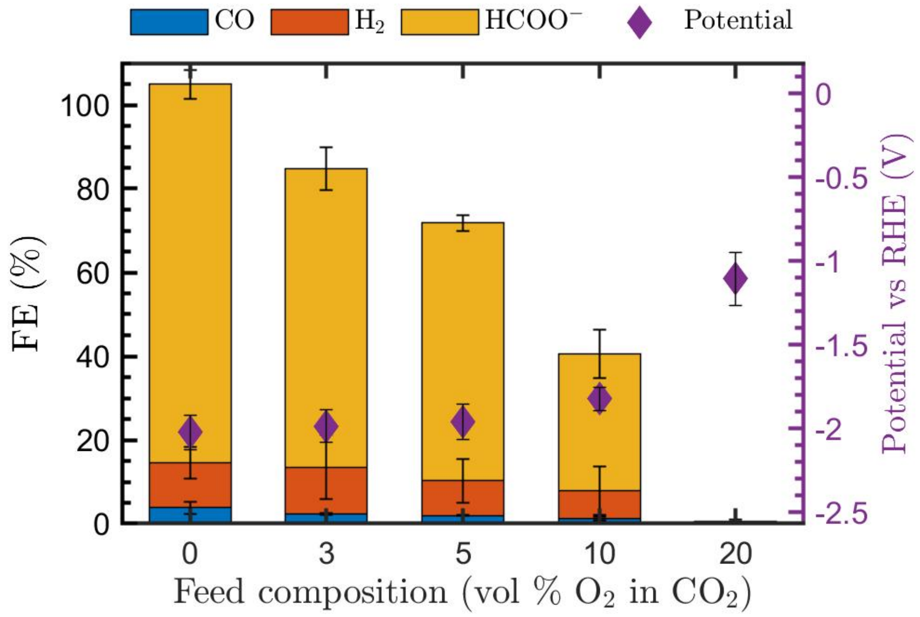

Figure S.19: All results for the PTFE-Bi_2O_3 GDE with busbars at 100 mA cm^{-2}, related to Fig. 5.

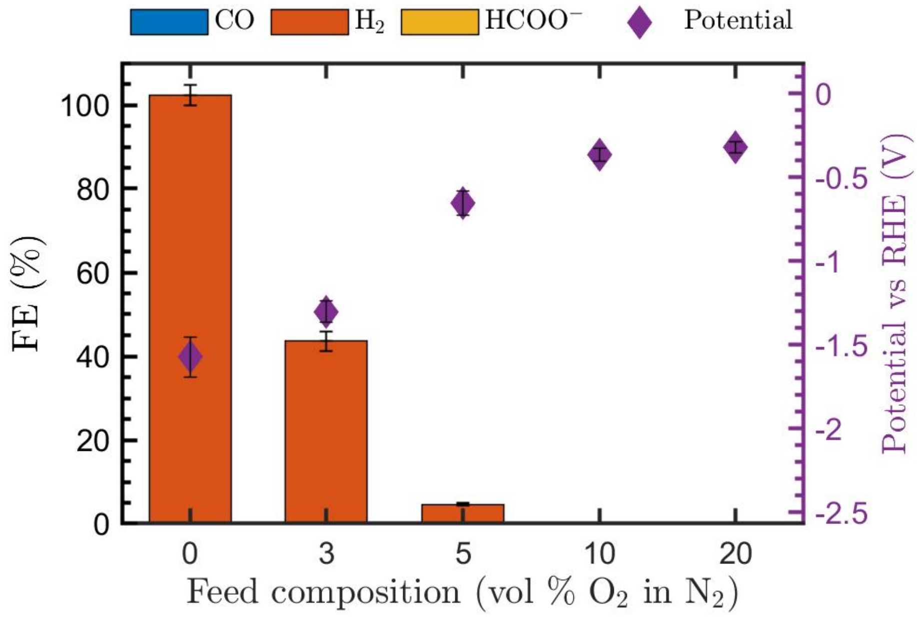

Figure S.20: Results for the 39BB-Ag GDE at 100 mA cm^{-2} with N_2 as balance gas.

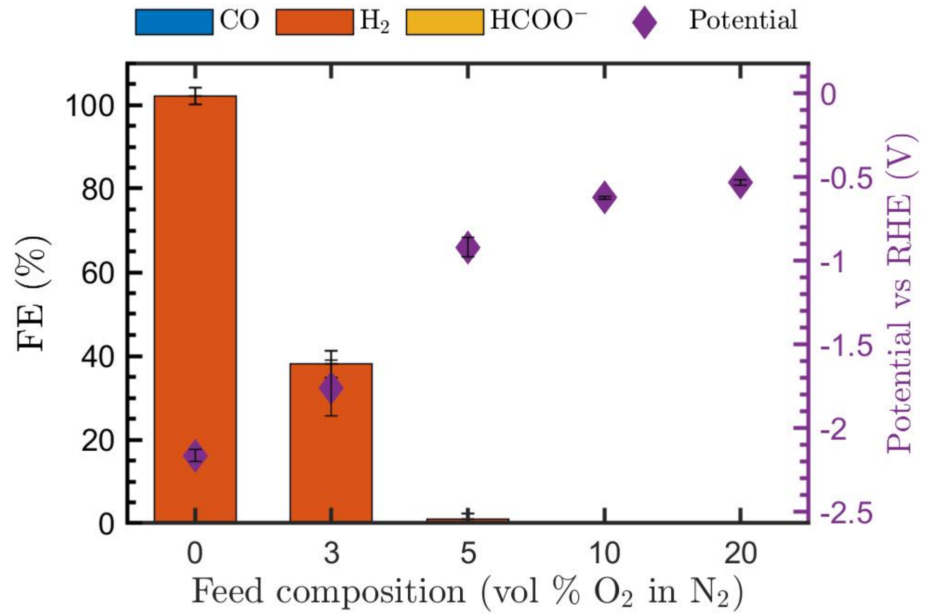

Figure S.21: Results for the 39BB-Bi_2O_3 GDE at 100 mA cm^{-2} with N_2 as balance gas.

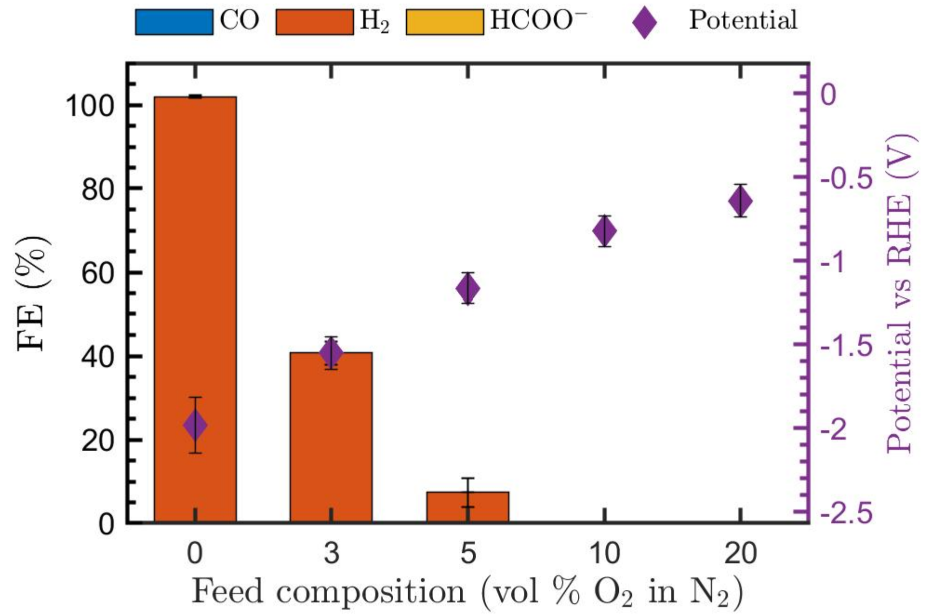

Figure S.22: Results for the bare 39BB GDL at 100 mA cm^{-2} with N_2 as balance gas.

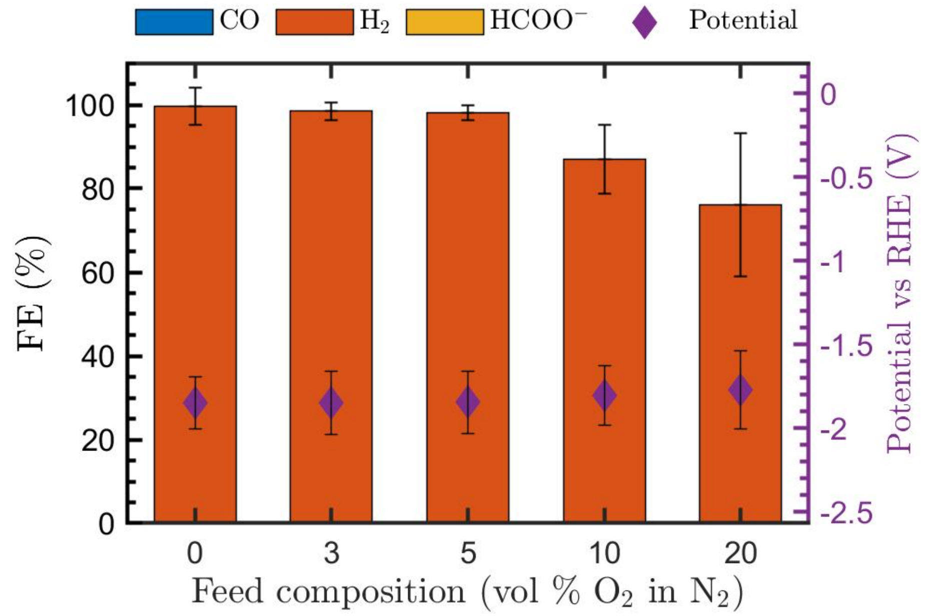

Figure S.23: Results for the bare Ti felt at 100 mA cm^{-2} with N_2 as balance gas.

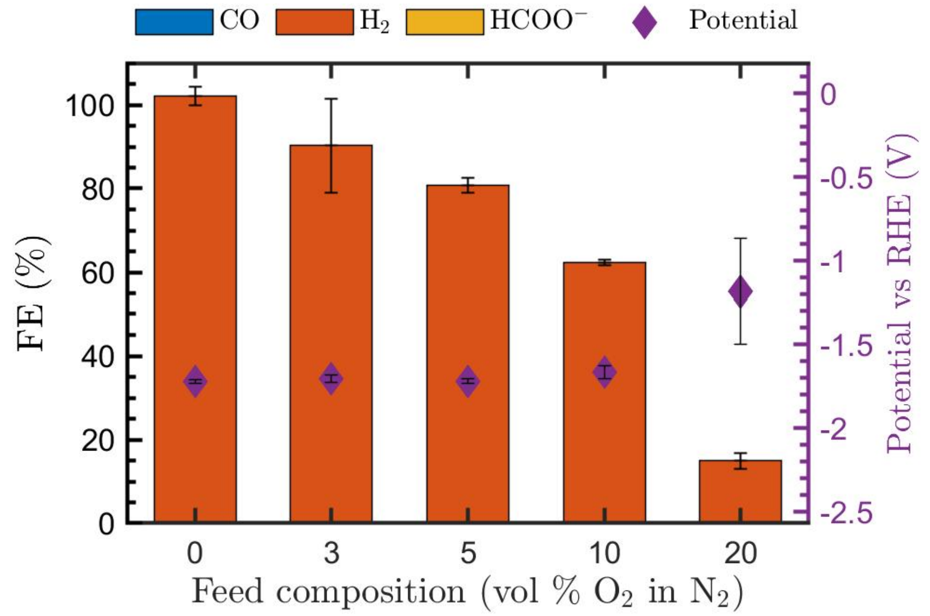

Figure S.24: Results for the PTFE-Ag GDE at 100 mA cm^{-2} with N_2 as balance gas.

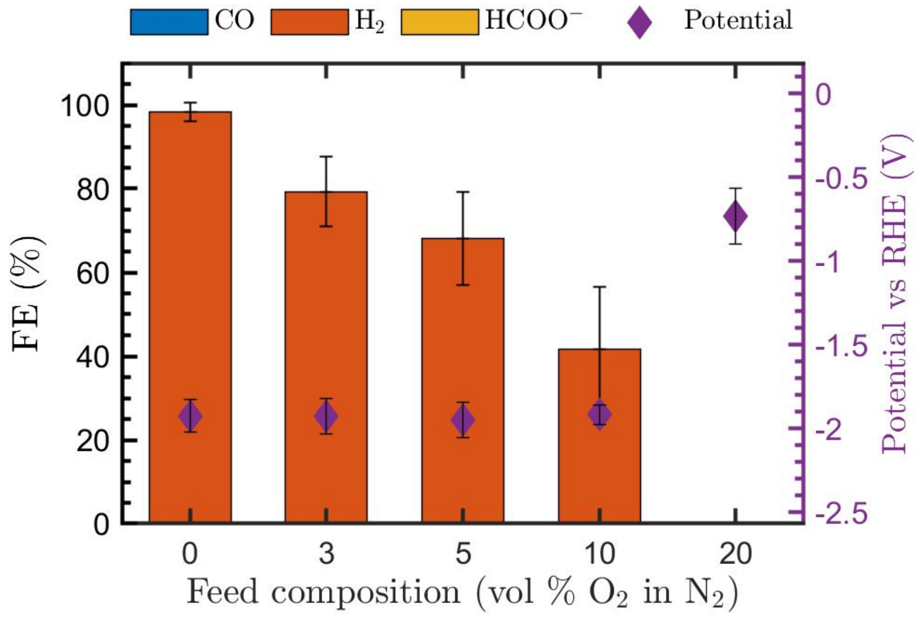

Figure S.25: Results for the PTFE-Bi_2O_3 GDE with a conductive sublayer at 100 mA cm^{-2} with N_2 as balance gas.

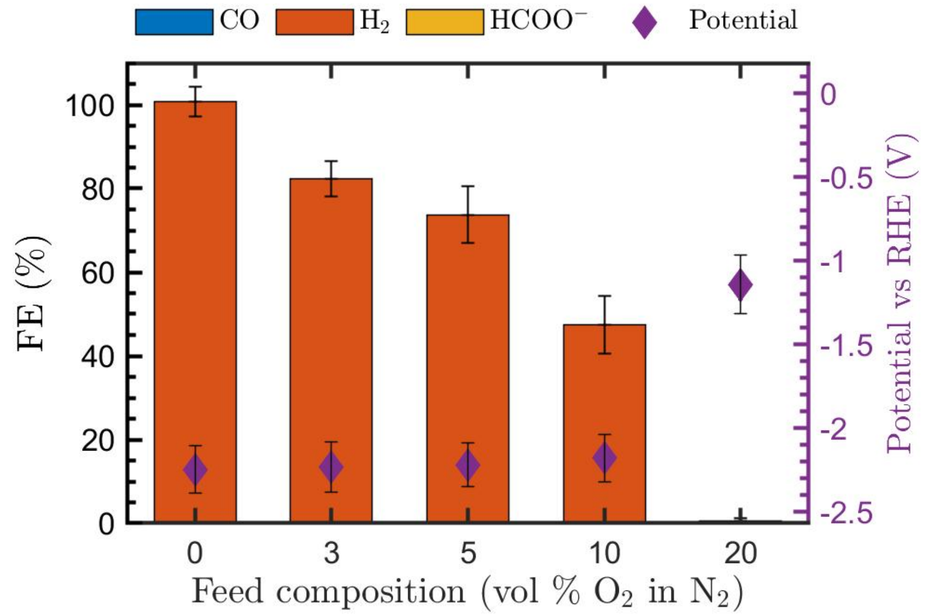

Figure S.26: Results for the PTFE-Bi_2O_3 GDE with busbars at 100 mA cm^{-2} with N_2 as balance gas.

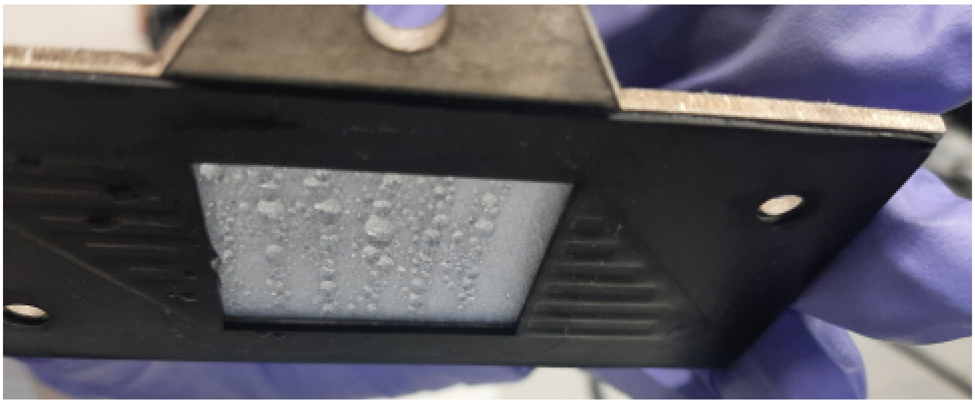

Figure S.27: Picture of the PP side (facing gas chamber) after electrolysis at 100 mA cm^{-2}. The droplets are aligned with the busbars, indicating that perspiration occured mostly in line with the busbars due to electrowetting in these highly conductive zones.

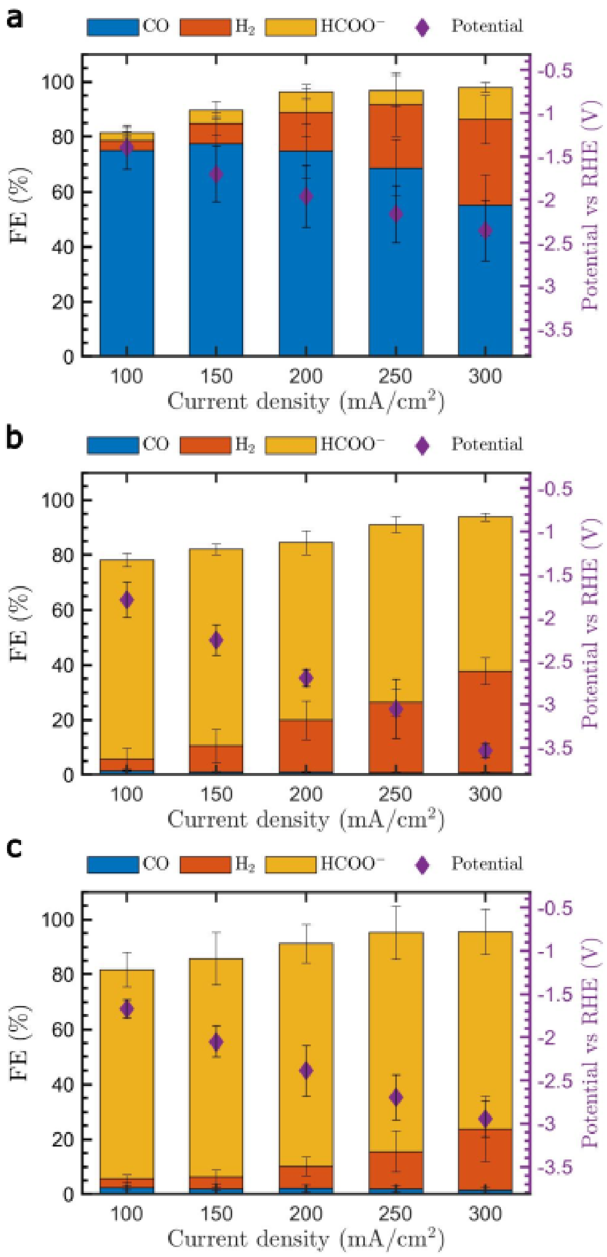

Figure S.28: Faradaic efficiency and potential for a 100 mL/min 3% O_2 in CO_2 feed stream at current densities up to 300 mA/cm^2 for **a** PTFE-Ag, **b** PTFE-Bi_2O_3 sublayer, **c** PTFE-Bi_2O_3 busbars.

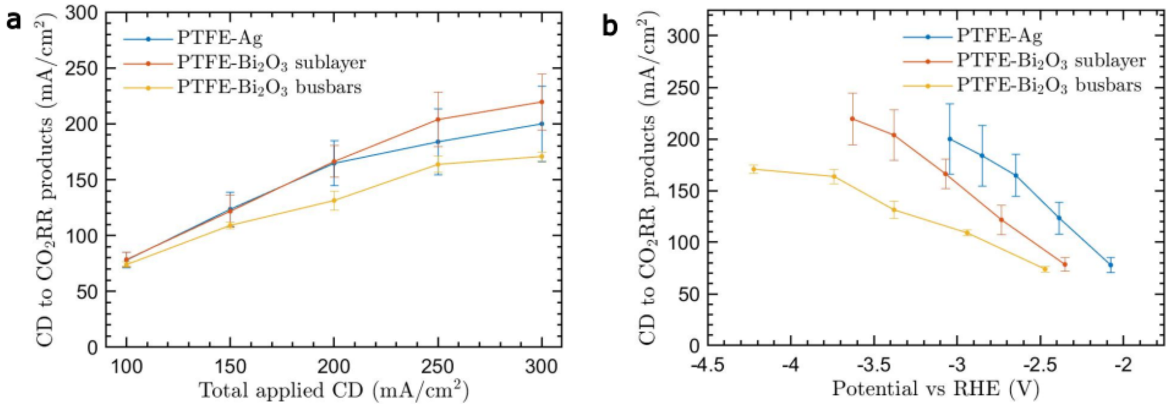

Figure S.29: Partial current density to CO_2RR products for three GDE systems plotted against **a** total applied current density and **b** potential vs RHE. The feed stream consisted of 3% O_2 in CO_2.

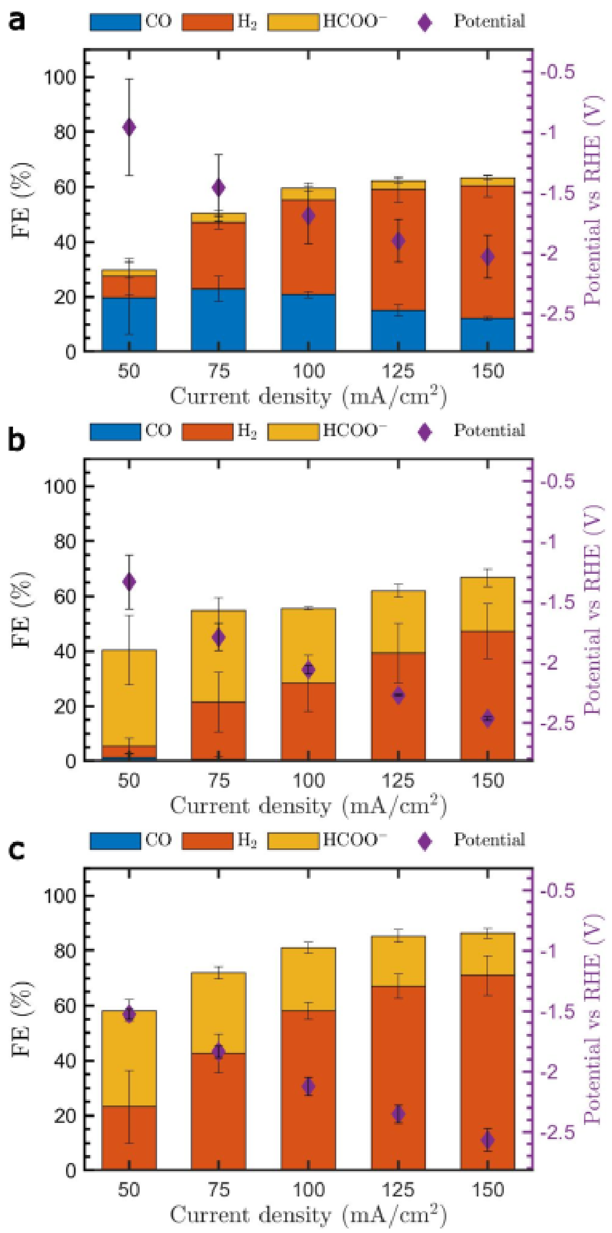

Figure S.30: Faradaic efficiency and potential for a 100 mL/min simulated flue gas (15% CO_2 + 4% O_2 in N_2) as feed stream at current densities up to 150 mA/cm^2 for **a** PTFE-Ag, **b** PTFE-Bi_2O_3 sublayer, **c** PTFE-Bi_2O_3 busbars.

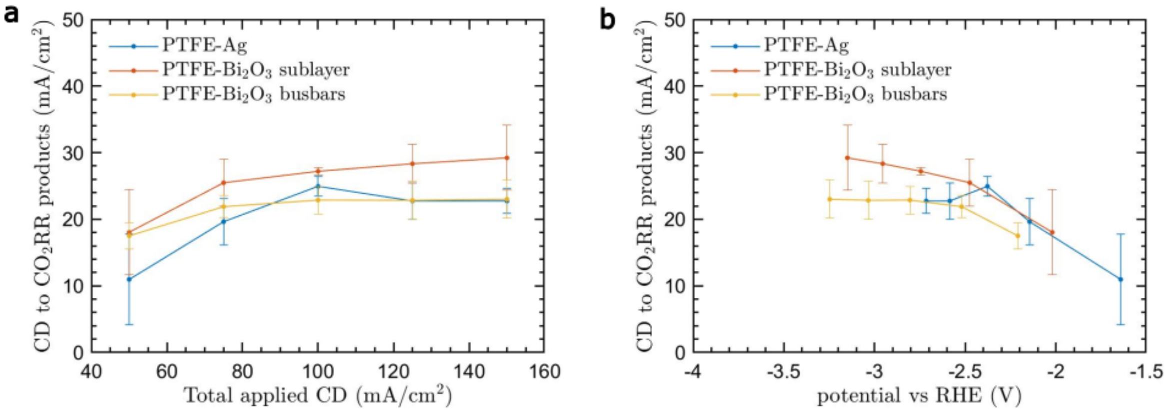

Figure S.31: Partial current density to CO_2RR products for three GDE systems plotted against **a** total applied current density and **b** potential vs RHE. The feed stream consisted of 15% CO_2 + 4% O_2 in N_2.

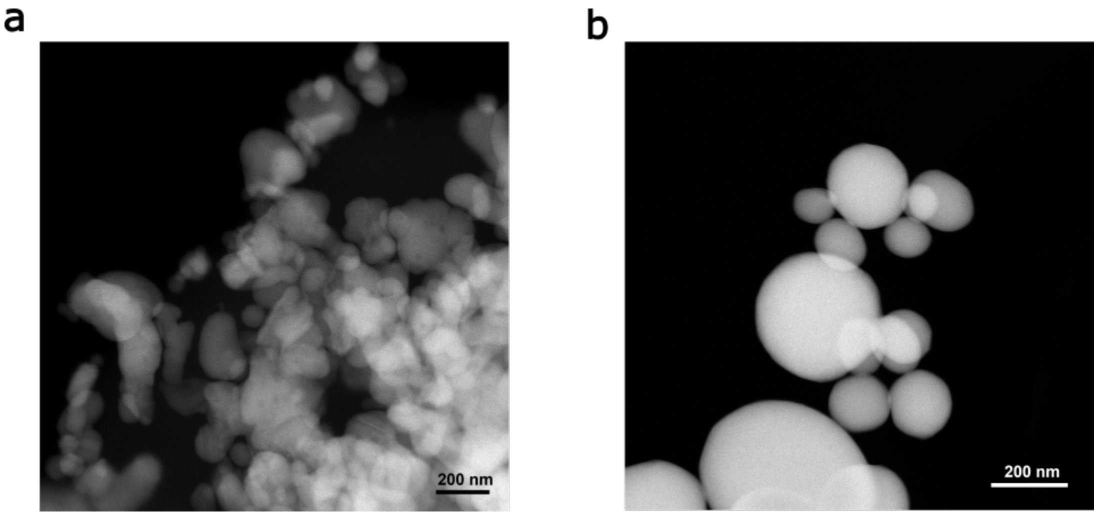

Figure S.32: Transmission electron microscopy images of the catalytic nanoparticles that were used in this work. **a** Ag nanoparticles. **b** Bi_2O_3 nanoparticles.

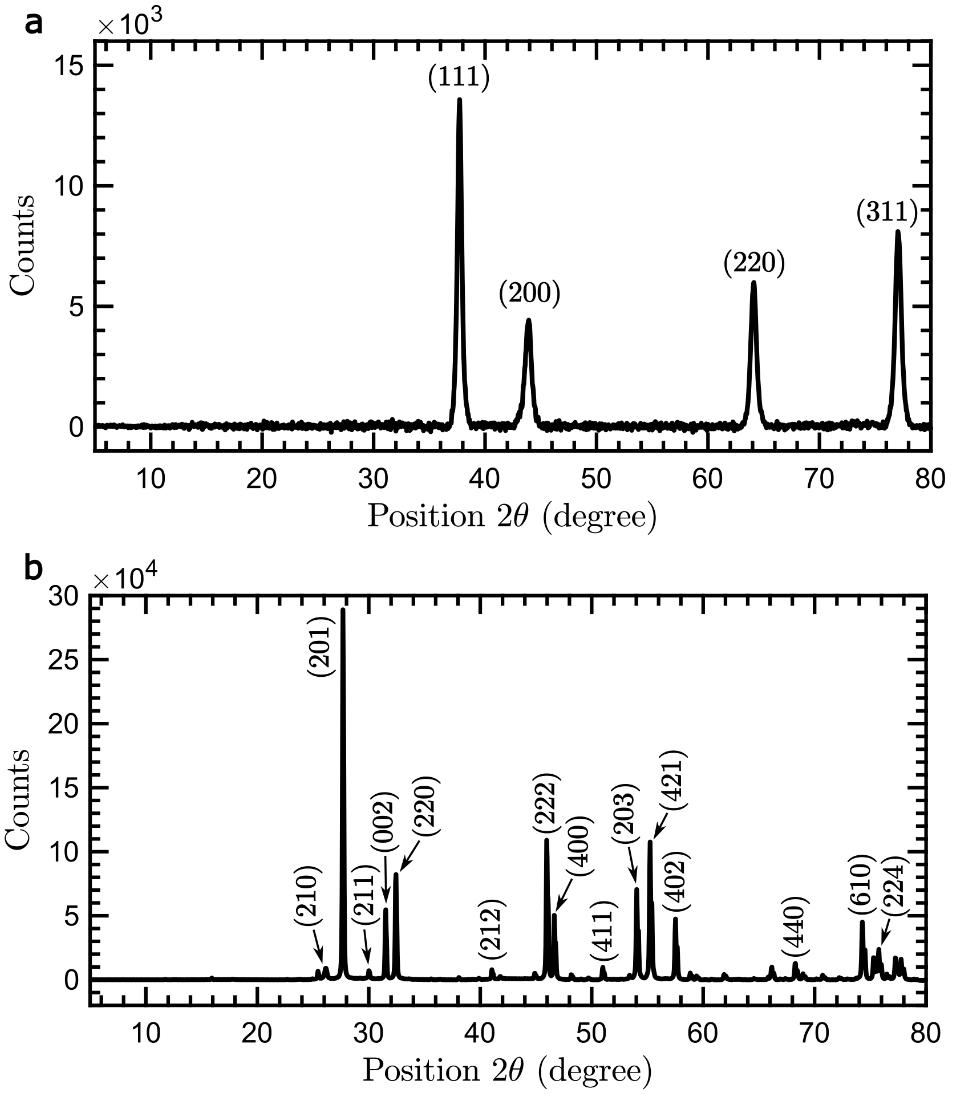

Figure S.33: X-ray diffraction results of the catalytic nanoparticles that were used in this work. **a** Ag nanoparticles, corresponding to JCPDS card number 04-0783. **b** Bi_2O_3 nanoparticles, corresponding to JCPDS card number 27-0050.

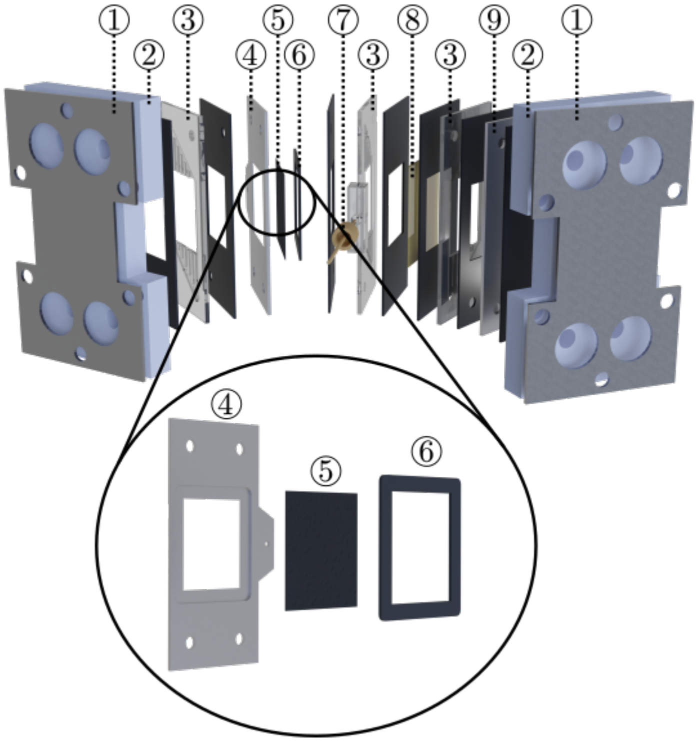

Figure S.34: The electrochemical flow reactor that was used during this study. 1) metal backplate, 2) PTFE backplate 3) PMMA gas- and liquid flow plates, 4) Titanium cathode frame, 5) gas diffusion electrode, 6) Viton gasket for GDE, 7) Ag/AgCl reference electrode, 8) Nafion 117 cation exchange membrane, 9) Platinized titanium electrode. All parts are separated by rubber gaskets. During operation with polymer-based GDEs, the Viton gasket (part 6) is changed to a titanium seal.

Supplemental Tables

Table S.1: Experimental data for the carbon-based 39BB GDE with Ag nanoparticles, related to Fig. 2.

% O_2 in CO_2	FE_{H_2} (%)	σ	FE_{CO} (%)	σ	FE_{HCOO^-} (%)	σ	V vs Ag/AgCl	σ	V vs RHE	FE_{tot} (%)	σ
0	0.87	1.23	95.33	1.35	3.49	2.41	-2.25	0.12	-1.57	99.69	3.03
3	0.56	0.80	46.76	7.19	0.68	0.11	-1.97	0.02	-1.29	48.00	7.24
5	0.16	0.23	10.10	9.71	0.12	0.08	-1.51	0.21	-0.83	10.39	9.71
10	0.00	0.00	0.01	0.01	0.00	0.01	-1.04	0.05	-0.36	0.01	0.01
20	0.00	0.00	0.01	0.02	0.00	0.00	-0.96	0.05	-0.27	0.01	0.02

Table S.2: Experimental data for the carbon-based 39BB GDE with Bi_2O_3 nanoparticles, related to Fig. 2.

% O_2 in CO_2	FE_{H_2} (%)	σ	FE_{CO} (%)	σ	FE_{HCOO^-} (%)	σ	V vs Ag/AgCl	σ	V vs RHE	FE_{tot} (%)	σ
0	0.14	0.20	3.55	0.23	94.98	1.82	-2.32	0.01	-1.64	98.67	1.85
3	0.25	0.01	1.56	0.58	37.26	7.50	-2.13	0.06	-1.44	39.07	7.52
5	0.00	0.00	0.53	0.56	10.76	7.79	-1.87	0.24	-1.19	11.29	7.81
10	0.00	0.00	0.00	0.00	0.00	0.00	-1.57	0.21	-0.89	0.00	0.00
20	0.00	0.00	0.00	0.00	0.00	0.00	-1.35	0.01	-0.67	0.00	0.00

Table S.3: Experimental data for the bare 39BB carbon-based GDL, related to Fig. 2.

% O_2 in CO_2	FE_{H_2} (%)	σ	FE_{CO} (%)	σ	FE_{HCOO^-} (%)	σ	V vs Ag/AgCl	σ	V vs RHE	FE_{tot} (%)	σ
0	97.55	2.95	0.12	0.12	4.23	1.39	-2.86	0.14	-2.18	101.89	3.26
3	49.18	5.90	0.07	0.07	2.02	0.73	-2.51	0.08	-1.83	51.27	5.95
5	19.29	7.32	0.03	0.04	1.29	1.57	-2.12	0.08	-1.43	20.61	7.49
10	0.00	0.00	0.00	0.00	0.00	0.00	-1.55	0.08	-0.86	0.00	0.00
20	0.00	0.00	0.00	0.00	0.00	0.00	-1.47	0.03	-0.79	0.00	0.00

Table S.4: Experimental data for the bare Ti felt after hydrophobic treatment, related to Fig. 2.

% O_2 in CO_2	FE_{H_2} (%)	σ	FE_{CO} (%)	σ	FE_{HCOO^-} (%)	σ	V vs Ag/AgCl	σ	V vs RHE	FE_{tot} (%)	σ
0	100.82	3.14	0.00	0.00	0.10	0.10	-2.52	0.13	-1.84	100.92	3.14
3	96.73	1.84	0.00	0.00	0.09	0.08	-2.57	0.15	-1.89	96.82	1.85
5	94.88	1.09	0.00	0.00	0.14	0.02	-2.54	0.16	-1.85	95.02	1.09
10	89.81	6.84	0.00	0.00	0.09	0.07	-2.56	0.17	-1.88	89.90	6.85
20	78.12	11.07	0.00	0.00	0.13	0.01	-2.53	0.21	-1.85	78.24	11.07

Table S.5: Experimental data for the Ag-coated PTFE substrate, related to Fig. 3.

% O_2 in CO_2	FE_{H_2} (%)	σ	FE_{CO} (%)	σ	FE_{HCOO^-} (%)	σ	V vs Ag/AgCl	σ	V vs RHE	FE_{tot} (%)	σ
0	7.81	2.50	85.17	4.63	4.70	5.20	-2.17	0.22	-1.49	97.67	7.40
3	6.38	5.94	63.57	4.38	5.62	3.57	-2.13	0.26	-1.45	75.57	8.20
5	7.07	6.61	50.47	7.26	4.55	3.07	-2.05	0.20	-1.37	62.09	10.28
10	2.62	2.62	29.59	11.76	2.31	1.11	-1.41	0.03	-0.73	34.52	12.10
20	0.18	0.25	3.01	4.91	1.26	2.18	-0.89	0.02	-0.21	4.45	5.38

Table S.6: Experimental data for the PTFE-Bi_2O_3 GDE with a conductive sublayer, related to Fig. 5.

% O_2 in CO_2	FE_{H_2} (%)	σ	FE_{CO} (%)	σ	FE_{HCOO^-} (%)	σ	V vs Ag/AgCl	σ	V vs RHE	FE_{tot} (%)	σ
0	7.14	0.62	7.55	3.78	90.12	2.43	-2.36	0.09	0.77	104.81	4.54
3	4.81	1.46	5.13	4.00	70.90	5.59	-2.29	0.07	0.75	80.84	7.03
5	3.47	2.71	5.00	4.05	59.33	9.13	-2.25	0.10	0.78	67.79	10.35
10	0.95	1.08	4.81	4.02	29.75	11.60	-2.19	0.08	0.76	35.51	12.32
20	0.00	0.00	0.00	0.00	1.12	1.00	-1.28	0.12	0.80	1.12	1.00

Table S.7: Experimental data for the PTFE-Bi_2O_3 GDE with conductive busbars, related to Fig. 5.

% O_2 in CO_2	FE_{H_2} (%)	σ	FE_{CO} (%)	σ	FE_{HCOO^-} (%)	σ	V vs Ag/AgCl	σ	V vs RHE	FE_{tot} (%)	σ
0	10.72	3.73	3.81	1.41	90.38	3.45	-2.70	0.10	-2.02	104.91	5.27
3	11.15	7.65	2.34	0.24	71.35	5.09	-2.67	0.09	-1.98	84.84	9.20
5	8.33	5.22	2.00	0.22	61.51	1.88	-2.64	0.11	-1.96	71.84	5.55
10	6.69	5.82	1.27	0.41	32.65	5.72	-2.51	0.07	-1.82	40.61	8.17
20	0.00	0.00	0.13	0.18	0.32	0.46	-1.79	0.16	-1.11	0.45	0.49

Table S.8: Experimental data for 39BB-Ag GDE with N_2 as balance gas.

% O_2 in N_2	FE_{H_2} (%)	σ	FE_{CO} (%)	σ	FE_{HCOO^-} (%)	σ	V vs Ag/AgCl	σ	V vs RHE	FE_{tot} (%)	σ
0	102.37	2.48	0.00	0.00	0.00	0.00	-2.26	0.12	-1.57	102.37	2.48
3	43.59	2.38	0.00	0.00	0.00	0.00	-1.99	0.06	-1.30	43.59	2.38
5	4.67	0.41	0.00	0.00	0.00	0.00	-1.34	0.07	-0.65	4.67	0.41
10	0.00	0.00	0.00	0.00	0.00	0.00	-1.05	0.04	-0.37	0.00	0.00
20	0.00	0.00	0.00	0.00	0.00	0.00	-1.00	0.03	-0.32	0.00	0.00

Table S.9: Experimental data for 39BB-Bi_2O_3 GDE with N_2 as balance gas.

% O_2 in N_2	FE_{H_2} (%)	σ	FE_{CO} (%)	σ	FE_{HCOO^-} (%)	σ	V vs Ag/AgCl	σ	V vs RHE	FE_{tot} (%)	σ
0	102.14	1.97	0.00	0.00	0.00	0.00	-2.85	0.04	-2.16	102.14	1.97
3	38.00	3.19	0.00	0.00	0.00	0.00	-2.44	0.17	-1.76	38.00	3.19
5	1.01	1.42	0.00	0.00	0.00	0.00	-1.60	0.06	-0.92	1.01	1.42
10	0.00	0.00	0.00	0.00	0.00	0.00	-1.31	0.01	-0.62	0.00	0.00
20	0.00	0.00	0.00	0.00	0.00	0.00	-1.21	0.02	-0.53	0.00	0.00

Table S.10: Experimental data for a bare 39BB GDL with N_2 as balance gas.

% O_2 in N_2	FE_{H_2} (%)	σ	FE_{CO} (%)	σ	FE_{HCOO^-} (%)	σ	V vs Ag/AgCl	σ	V vs RHE	FE_{tot} (%)	σ
0	101.94	0.34	0.00	0.00	0.00	0.00	-2.66	0.17	-1.98	101.94	0.34
3	40.70	2.73	0.00	0.00	0.00	0.00	-2.23	0.10	-1.55	40.70	2.73
5	7.34	3.41	0.00	0.00	0.00	0.00	-1.85	0.09	-1.16	7.34	3.41
10	0.00	0.00	0.00	0.00	0.00	0.00	-1.51	0.09	-0.82	0.00	0.00
20	0.00	0.00	0.00	0.00	0.00	0.00	-1.32	0.10	-0.64	0.00	0.00

Table S.11: Experimental data for the bare Ti felt with N_2 as balance gas.

% O_2 in N_2	FE_{H_2} (%)	σ	FE_{CO} (%)	σ	FE_{HCOO^-} (%)	σ	V vs Ag/AgCl	σ	V vs RHE	FE_{tot} (%)	σ
0	99.63	4.40	0.00	0.00	0.00	0.00	-2.53	0.16	-1.85	99.63	4.40
3	98.49	2.12	0.00	0.00	0.00	0.00	-2.53	0.19	-1.85	98.49	2.12
5	98.13	1.69	0.00	0.00	0.00	0.00	-2.53	0.19	-1.85	98.13	1.69
10	87.07	8.23	0.00	0.00	0.00	0.00	-2.49	0.18	-1.80	87.07	8.23
20	76.22	17.11	0.00	0.00	0.00	0.00	-2.46	0.23	-1.77	76.22	17.11

Table S.12: Experimental data for PTFE-Ag GDE with N_2 as balance gas.

% O_2 in N_2	FE_{H_2} (%)	σ	FE_{CO} (%)	σ	FE_{HCOO^-} (%)	σ	V vs Ag/AgCl	σ	V vs RHE	FE_{tot} (%)	σ
0	102.08	2.21	0.00	0.00	0.00	0.00	-2.40	0.01	-1.72	102.08	2.21
3	90.32	11.21	0.00	0.00	0.00	0.00	-2.39	0.02	-1.70	90.32	11.21
5	80.71	1.75	0.00	0.00	0.00	0.00	-2.40	0.01	-1.72	80.71	1.75
10	62.25	0.65	0.00	0.00	0.00	0.00	-2.35	0.04	-1.67	62.25	0.65
20	14.89	1.84	0.00	0.00	0.00	0.00	-1.86	0.32	-1.18	14.89	1.84

Table S.13: Experimental data for PTFE-Bi_2O_3 GDE with a conductive sublayer and N_2 as balance gas.

% O_2 in N_2	FE_{H_2} (%)	σ	FE_{CO} (%)	σ	FE_{HCOO^-} (%)	σ	V vs Ag/AgCl	σ	V vs RHE	FE_{tot} (%)	σ
0	98.38	2.22	0.00	0.00	0.00	0.00	-2.61	0.10	-1.93	98.38	2.22
3	79.29	8.38	0.00	0.00	0.00	0.00	-2.61	0.11	-1.93	79.29	8.38
5	68.15	11.07	0.00	0.00	0.00	0.00	-2.63	0.11	-1.95	68.15	11.07
10	41.64	14.94	0.00	0.00	0.00	0.00	-2.60	0.06	-1.92	41.64	14.94
20	0.00	0.00	0.00	0.00	0.00	0.00	-1.41	0.17	-0.73	0.00	0.00

Table S.14: Experimental data for PTFE-Bi_2O_3 GDE with busbars and N_2 as balance gas.

% O_2 in CO_2	FE_{H_2} (%)	σ	FE_{CO} (%)	σ	FE_{HCOO^-} (%)	σ	V vs Ag/AgCl	σ	V vs RHE	FE_{tot} (%)	σ
0	100.80	3.47	0.00	0.00	0.00	0.00	-2.93	0.14	-2.25	100.80	3.47
3	82.29	4.26	0.00	0.00	0.00	0.00	-2.92	0.15	-2.23	82.29	4.26
5	73.75	6.83	0.00	0.00	0.00	0.00	-2.90	0.13	-2.22	73.75	6.83
10	47.47	6.85	0.00	0.00	0.00	0.00	-2.86	0.14	-2.18	47.47	6.85
20	0.50	0.70	0.00	0.00	0.00	0.00	-1.82	0.17	-1.14	0.50	0.70

Table S.15: ICP-MS measurements on the catholyte outflow after 50 h stability measurements.

	Ag (ppm)	Bi (ppm)
Blank 0.5 M $KHCO_3$ catholyte	Not detected.	$6.27 \cdot 10^{-5}$
PTFE-Ag	Not detected.	$2.32 \cdot 10^{-3}$
PTFE-Bi_2O_3 with Ag sublayer	Not detected.	$1.02 \cdot 10^{-2}$
PTFE-Bi_2O_3 with Ag busbars	Not detected.	$8.16 \cdot 10^{-4}$

Table S.16: In-plane (IP) and through-plane (TP) properties of the Sigracet 39BB gas diffusion layer according to the manufacturer SGL carbon.

Property	Value	Unit
Thickness	315	µm
TP gas permeability	1.5	Gurley sec
TP gas permeability at 1 Mpa	0.12	10^{-12} m^2
IP gas permeability	8.4	10^{-12} m^2
TP area specific resistance (1 MPa)	< 13	mΩ cm^2
IP electric resistance	0.56	Ω mm
TP thermal conductivity	0.2	$Wm^{-1}K^{-1}$

Table S.17: Properties of the Aspire laminated PTFE filter membrane with 0.2 µm pores according to the manufacturer Sterlitech.

Property	Value	Unit
Thickness	152-254	µm
Clean air flow at 70 mbar	0.20-0.51	L/min·cm
Clean water flow at 0.7 bar	0	mL/min·cm
Water entry pressure	> 45	psi
