# Supplementary material for: Promoting CO2 reduction in the presence of oxygen with polymer-based gas diffusion electrodes
Source: Chem Catal. 2025 Jul 17;5(7):101353. doi: 10.1016/j.checat.2025.101353 (PMC12271024; doi:10.1016/j.checat.2025.101353)
Supplement: Document S2. Article plus supplemental information [file mmc3.pdf]

Chem Catalysis

Article

Promoting CO_2 reduction in the presence of oxygen with polymer-based gas diffusion electrodes

Graphical abstract

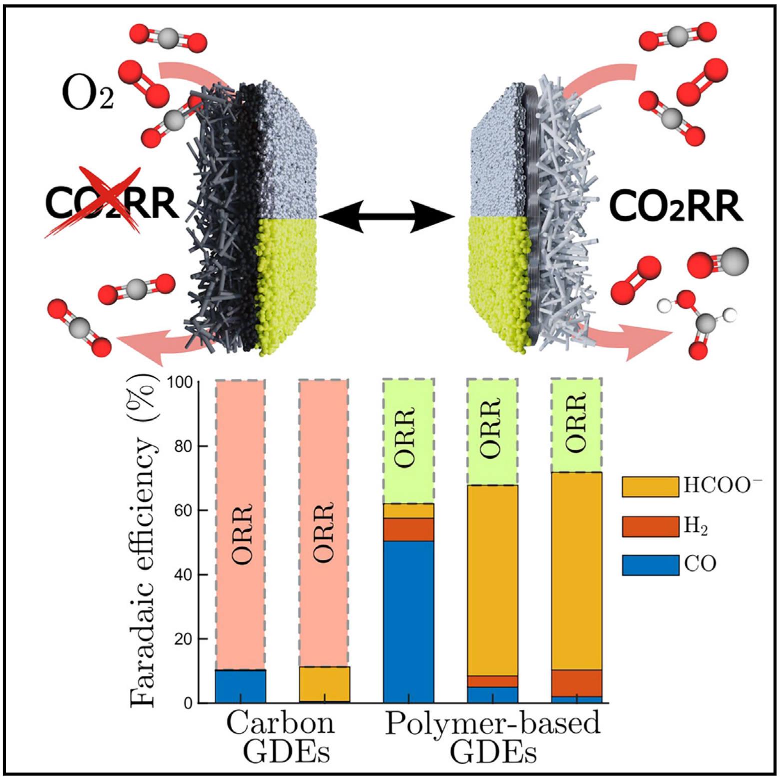

Authors

Sam Van Daele, Lieven Hintjens, Daniel Choukroun, Nick Daems, Jonas Hereijgers, Tom Breugelmans

Correspondence

tom.breugelmans@uantwerpen.be

In brief

Polymer-based gas diffusion electrodes (GDEs) allow for more oxygen-tolerant CO_2 electroreduction than conventional carbon-based GDEs. These electrodes enable impurity-resistant CO_2 conversion to valuable products, reducing the need for extensive purification steps. This advancement boosts the economic viability of electrochemical CO_2 reduction toward industrial applications.

Highlights

- Unravelling the role of the GDL material during CO_2 reduction with O_2 impurities
- Limiting the parasitic oxygen evolution reaction with PTFE gas diffusion electrodes
- 50 h stable electrolyzer operation with an impure CO_2 feed stream

Van Daele et al., 2025, Chem Catalysis *5*, 101353
July 17, 2025 © 2025 The Authors. Published by Elsevier Inc.
https://doi.org/10.1016/j.checat.2025.101353

Chem Catalysis

CellPress
OPEN ACCESS

Article

Promoting CO_2 reduction in the presence of oxygen with polymer-based gas diffusion electrodes

Sam Van Daele,[1] Lieven Hintjens,[1] Daniel Choukroun,[1] Nick Daems,[1] Jonas Hereijgers,[1] and Tom Breugelmans[1,2,*]
[1]Research Group Applied Electrochemistry & Catalysis (ELCAT), University of Antwerp, Faculty of Applied Engineering, Universiteitsplein 1, 2610 Wilrijk, Antwerp, Belgium
[2]Lead contact
*Correspondence: tom.breugelmans@uantwerpen.be
https://doi.org/10.1016/j.checat.2025.101353

THE BIGGER PICTURE CO_2 emissions drive climate change and require urgent abatement. Direct electrochemical conversion of greenhouse gas into value-added chemicals presents great prospects, yet the inherently high costs of CO_2 capture and purification weigh on its profitability. One solution is to directly use flue gas exhaust streams that contain CO_2 with unreacted O_2, but the competition between the two for electrons hampers the efficiency of the process. Here, we develop a polymer-based electrode architecture that provides the right balance of in-plane conductivity and optimal CO_2 conversion at the expense of O_2 diffusion, thus suppressing the major parasitic reactions. As a case in point, our electrodes boost efficiency by more than 40% at a high O_2 concentration of 5% and maintain stability for >2 days. This breakthrough is bound to enable the direct utilization of waste CO_2 streams from a multitude of industrial point sources.

SUMMARY

The electrochemical reduction of CO_2 is a promising technology that holds the potential to convert waste CO_2 into valuable products. High carbon capture and purification costs hamper economic feasibility and drive scientists to explore the viability of directly using flue gas exhaust streams. However, flue gas impurities, such as O_2, pose a great challenge because O_2 is preferentially reduced over CO_2. Here, we show that careful design of the gas diffusion electrode (GDE) can significantly improve Faradaic efficiency. This work not only unravels how commonly used carbon-based GDEs facilitate O_2 reduction but also succeeds in devising polymer-based alternatives that significantly improve the Faradaic efficiency (>40%) of CO_2 reduction with 5% O_2-containing feed streams while showing excellent stability for >2 days. These results demonstrate that it is feasible to engineer suitable GDEs for CO_2 reduction with impure feed streams.

INTRODUCTION

In the face of global concerns regarding climate change, reducing CO_2 emissions stands out as one of the important challenges of our time.[1] The pressing need to mitigate CO_2 emissions has led to an increasing interest in carbon capture and utilization (CCU) technologies, such as the electrochemical CO_2 reduction reaction (CO_2RR), which holds the potential to convert CO_2 into useful chemicals and fuels.[2] An additional benefit of this electrochemical process is its compatibility with an intermittent electricity supply from renewable energy sources (e.g., solar or wind power).[3] Converting CO_2 into CO and formate or formic acid is of special interest in this work because these reaction products benefit from high revenue per mole of electron transferred and can both be produced at a Faradaic efficiency (FE) exceeding 90%.[4,5]

One of the most important advances in CO_2 reduction research was the transition from the conventional batch H-cell, where the system was limited by the sluggish mass transport of CO_2 in aqueous electrolytes, to more advanced continuous-flow cell configurations (CO_2 electrolyzers) that ensure shorter CO_2 diffusional lengths.[6,7] The key to this transition was the employment of gas diffusion electrodes (GDEs) that allow a supply of gaseous CO_2 to the cell and thereby improve CO_2 mass transport to facilitate electrolyzer operation at current densities well above 100 mA cm^{-2} to CO_2RR products.[8] Figure 1A illustrates the structure of a typical carbon-based GDE consisting of a carbon fiber support (CFS) topped with a microporous layer (MPL), whereupon catalytic particles are coated to form the catalyst layer (CL).[9] The MPL (Figures 1B and 1C) consists of carbon that has undergone a polymeric treatment, usually with polytetrafluorethylene (PTFE), to increase the hydrophobic properties of this layer and prevent liquid from penetrating the GDE.[10] This is an essential property because excessive electrolyte seepage through the GDE (i.e., flooding) limits the stability of electrolyzer operation.[11] The CFS (Figures 1D and 1E) consists

This is an open access article under the CC BY license (http://creativecommons.org/licenses/by/4.0/).

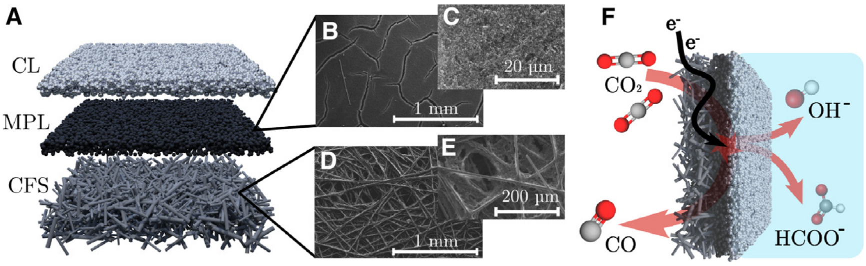

Figure 1. Illustration of a gas diffusion electrode

(A) Representation of the carbon fiber support (CFS), microporous layer (MPL), and catalyst layer (CL).

(B and C) Scanning electron microscopy (SEM) images of the MPL.

(D and E) SEM images of the CFS.

(F) Schematic of the gas diffusion electrode (GDE) during CO_2 electrolysis.

of a macroporous carbon fiber paper and governs the overall mechanical properties of the GDE. In this study, when referring to a carbon-based GDE, we utilized the Sigracet 39BB gas diffusion layer (GDL; CFS + MPL), which contains 5% (w/w) PTFE in the CFS and 20%–25% (w/w) PTFE in the MPL according to the manufacturer's technical properties sheet.[12] A comparative study between different PTFE GDEs was conducted elsewhere.[13]

During electrolyzer operation, gaseous CO_2 from the feed stream diffuses through the CFS and MPL to reach the CL, where CO_2 adsorbs to its active sites and the CO_2RR can take place. Typical catalysts for the reaction toward CO (Equation 2) are Ag[11] and Au,[14] whereas formate is selectively produced (Equation 1) with mainly Bi[15] and Sn[16] catalysts. The CL exchanges gaseous species through the porous structure of the dry MPL and exchanges ionic species with the neighboring liquid phase (Figure 1F).[17] When the CO_2 supply to the CL's active sites is insufficient or no CO_2RR catalyst is present, H_2 is generated via the hydrogen evolution reaction (HER) (Equation 3). The CO_2RR and HER yield OH^- as byproducts, which can react with incoming CO_2 to form bicarbonate or carbonate according to Equation 4 or 5, respectively.

$$CO_2 + H_2O + 2e^- \rightarrow HCOO^- + OH^- \quad \text{(Equation 1)}$$

$$CO_2 + H_2O + 2e^- \rightarrow CO + 2OH^- \quad \text{(Equation 2)}$$

$$2H_2O + 2e^- \rightarrow H_2 + 2OH^- \quad \text{(Equation 3)}$$

$$OH^- + CO_2 \leftrightharpoons HCO_3^- \quad \text{(Equation 4)}$$

$$HCO_3^- + OH^- \leftrightharpoons CO_3^{2-} + H_2O \quad \text{(Equation 5)}$$

Over the last few years, different pilot-scale CO_2 electrolysis plants based on the utilization of purified CO_2 have been installed all over the world.[18] However, the costs associated with the CO_2-capture and -purification processes limit the economic viability of the technology.[19,20] Therefore, direct CO_2 conversion from flue gas has emerged as an interesting avenue for potentially bypassing the carbon-capture procedure or significantly reducing the number of purification steps necessary. This approach presents its own set of challenges, specifically the compatibility of the CO_2RR with flue gas impurities.

In flue gas point sources, CO_2 is typically present in a diluted form (13%–14% CO_2) with mainly >70% N_2, 3%–4% O_2, ~200 ppm SO_x, and ~200 ppm NO_x impurities (Singh and Berchtold, 2018, Project Review Meeting for Crosscutting Research Portfolios). Previous work has shown that diluted CO_2 streams lead to electrolyzer operation at a lower partial current density (CD) to C-products because there is limited CO_2 near the electrode.[21,22] The impacts of SO_x[23] and NO_x[24] during CO_2 electrolysis in continuous-flow cells have also been studied.[25] Because of their low abundance in flue gases, electrolyzer operation at 100 mA cm^{-2} for 20 h results in a stable operation without significant loss of FE or catalyst degradation.[26] O_2 in flue gas streams was initially hypothesized to be incompatible with the CO_2RR given the preferential reduction of O_2.[27] Indeed, we recently observed a significant loss of FE when we investigated the impact of O_2 during CO_2 electrolysis.[26] Preventing the oxygen reduction reaction (ORR) to sustain high FE for the CO_2RR remains a top priority in advancing the field of direct CO_2 electrolysis from flue gases, which is the aim of our present study.

In order to limit the ORR during the CO_2RR with O_2 impurities in the gas stream, this work delves into the role of the carbon-containing substrate material in facilitating the ORR during the CO_2RR, which has never—to the best of our knowledge—been studied before. The upcoming use of metal-free carbon GDEs in the field of oxygen reduction motivated a thorough exploration of the GDL material.[28,29] Here, we show that careful engineering of the GDE allows for a significant improvement in FE to CO_2RR products in the presence of O_2. Polymer-based GDEs effectively suppress the ORR on the GDE substrate itself and allow for a substantial increase in FE with O_2-containing CO_2 feed streams. In fact, any undesired electrochemical reaction that can take place outside the CL is prevented through the use of a non-conductive polymer-based GDL. This knowledge is critical for the future implementation of this technology and could allow industrial plants to bypass or limit the number of necessary purification steps.

RESULTS AND DISCUSSION

Role of the GDE substrate material

Initially, we examined the impact of various oxygen concentrations in the CO_2 feed stream on the FE. This investigation was conducted at a CD of 100 mA cm^{-2} for different oxygen concentrations (0%, 3%, 5%, 10%, and 20% O_2 in CO_2) that were fed to the reactor during a single run, and the results are shown in Figures 2A–2C. With a pure CO_2 feed (Figure 2A), the Ag-coated carbon paper produced CO at FE_{CO} = 95.3% ± 1.4%, and the

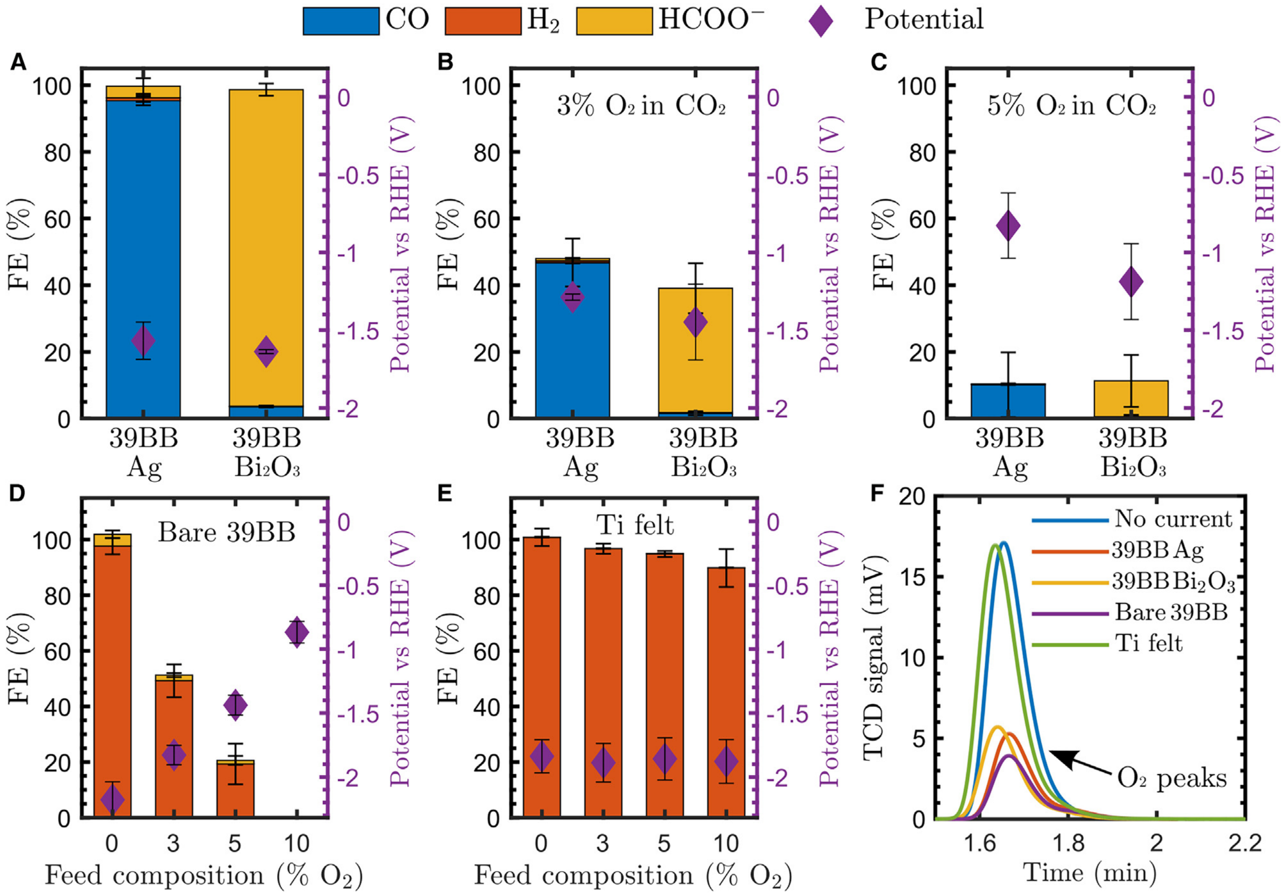

Figure 2. Impact of the GDE substrate material with O_2-containing CO_2 feed streams

(A–C) Ag- and Bi_2O_3-coated carbon paper (Sigracet 39BB) at 100 mA cm^{-2} with 0% (A), 3% (B), and 5% (C) O_2 in the CO_2 feed stream.

(D) Carbon paper without catalyst at 100 mA cm^{-2} with varying O_2 concentrations in the CO_2 feed.

(E) PTFE-treated Ti felt without catalyst at 100 mA cm^{-2} with varying O_2 concentrations in the CO_2 feed.

(F) GC slice at the retention time for O_2 detection with and without application of 100 mA cm^{-2} with 3% O_2 in the CO_2 feed stream for different GDEs.

Error bars represent the standard deviation from three measurements.

Bi_2O_3-coated carbon paper produced $HCOO^-$ at FE_{HCOO^-} = 95.0% ± 1.8%, both comparable to the state-of-the-art efficiencies with GDEs in flow cells.[30,31] The addition of 3% O_2 caused a >50% loss of total FE (Figure 2B), consistent with previous work.[26] The data of the complete run can be found in Figures S1 and S2. The FE decreased because the competitive ORR has a lower thermodynamic potential than the CO_2RR.[32] When the oxygen content increased even more to 5% (Figure 2C), the potential shifted to a less negative value because the ORR consumed almost all electrons (i.e., only ∼10% total FE was detected). We observed a potential difference (working electrode) between 39BB-Ag and 39BB-Bi_2O_3 because the former effectively catalyzes the ORR (i.e., it has a lower overpotential than Bi_2O_3 at the same CD), such that a lower overpotential is required for facilitating the ORR.[26,33] In this and previous work[26] with Ag and Bi_2O_3 catalysts, the total FEs for 39BB-Ag and 39BB-Bi_2O_3 were comparable, demonstrating that electron consumption by the ORR is similar for both target products. This indicates that O_2 availability at active sites is the limiting factor for the ORR and is correlated with O_2 feed composition.[26] It is worth mentioning that for copper catalysts in an H-cell, including O_2 in the CO_2 feed can increase the surface coverage of adsorbed hydroxyl species, improving the production rates of hydrocarbons and oxygenates.[34] In general, the formation of OH^- (from the CO_2RR, HER, or ORR) contributes to the local alkalinity at the electrode, which can both consume CO_2 to form HCO^- and even promote the generation of C_{2+} products with copper catalysts.[35–37]

We hypothesize that the carbon-based substrate material (Sigracet 39BB) also plays a crucial role in facilitating the ORR on GDEs for CO_2 reduction. In the field of proton-exchange membrane fuel cells, metal-free carbon catalysts are indeed gaining interest for an efficient ORR.[28,29] This suggests that carbon-based substrates might also facilitate the ORR and could therefore decrease the overall FE during the CO_2RR. To this end, we used a bare 39BB carbon paper without a catalyst as the GDE at 100 mA cm^{-2} and altered the feed composition every 25 min (Figure 2D). With pure CO_2, the near-unity FE (97.6% ± 3.0%)

was attributed to the HER, as expected in the absence of a CO_2RR catalyst. Interestingly, adding oxygen to the feed also lowered the overpotential and total FE, as observed in experiments with catalyst-coated 39BB carbon paper (Figures 2A–2C). To investigate the substrate effect further, we opted to test a Ti felt GDE (scanning electron microscopy [SEM] images in Figure S3). Because untreated Ti felt allows liquid to pass through, we treated the Ti felt with PTFE to provide a hydrophobic gas-liquid barrier. After the treatment, we achieved a static contact angle of 115° (Figure S4). The bare Ti felt electrode (without a catalyst) exclusively produced H_2 under pure CO_2 conditions. Interestingly, when we increased the O_2 feed concentration, the FE remained high. For example, the FE_{H2} still reached a value of 89.8% ± 6.8% even with 10% O_2 in CO_2. This observation highlights the importance of the type of substrate material. Although the bare Ti felt indicates a difference in ORR activity, the substrate cannot be used for efficient CO_2 electrolysis, as indicated by the additional Ag-coated Ti felt measurements in Figure S5. The FE_{CO} remained <40% as a result of the absence of a MPL[9] and the activity of Ti toward the HER.[38] For completeness, the data from the entire run are provided in Figures S6 and S7. The comparison between bare 39BB (Figure 2D) and Ti felt (Figure 2E) demonstrates that the GDE substrate material plays a crucial role in facilitating and, consequently, preventing the ORR when O_2 is present as a contaminant in industrial CO_2 streams. To validate these findings, we monitored the presence of O_2 in the reactor outflow with a gas chromatograph (GC), and the results for 3% O_2 in CO_2 are presented in Figure 2F. This chromatogram slice shows that less O_2 was present in the gas outflow when 100 mA cm^{-2} was applied for all carbon-based GDEs, demonstrating O_2 consumption during electrolysis. On the contrary, the Ti felt did not show less O_2 signal than the situation without current, indicating that the ORR did not occur noticeably with this substrate. These insights open up a new route for preventing the ORR during the CO_2RR by carefully engineering the GDE and, more specifically, the type of substrate material.

PTFE filter membrane GDE for CO production

The previous section showed that the GDE substrate material plays a crucial role in preventing the ORR when an O_2 contaminant is present in the CO_2 feed stream. In conventional GDEs, the carbon in both the CFS and MPL can aggravate the portion of unwanted ORR, reducing the FE to the desired reaction, although MPL has the greatest impact because of its high surface area. To completely exclude the ORR on the GDL, a non-conductive PTFE filter membrane is used to prevent any electrochemical reaction on this substrate. Our study used an Aspire laminated hydrophobic PTFE filter with 0.2 μm pores, a thickness of 152–254 μm, and a water entry pressure > 45 psi according to the manufacturer's technical specification sheet.[39]

Figure 3A illustrates the structure of the PTFE GDE. The polypropylene (PP) fibers (Figure 3B) serve as a backing layer for improved rigidity and allow gas transport to the PTFE filter. Similar to carbon-based gas diffusion media, an Ag catalyst is spray coated onto the porous layer to catalyze the CO_2RR. The 0.2-μm-wide pores of the PTFE filter (Figure 3C) allow gas diffusion to the CL, where the reaction takes place, while blocking the liquid electrolyte from entering the pores through its excellent hydrophobic properties.[40] A handful of studies have successfully used these kinds of polymer-based gas diffusion media, when coated with copper as the catalyst, for the electroreduction of CO_2 to multicarbon products.[35,41–49] When tested for over 150 h of electrolysis under a pure CO_2 feed, these polymer-based GDEs proved to be more stable than carbon-based GDEs.[35] Moreover, PTFE GDEs have the additional advantages of maintaining short CO_2 diffusion lengths through liquid and preventing flooding of the electrode, whereas carbon-based GDEs are classified as unsuitable for long-term use in a membrane electrode assembly because of flooding.[47] Detailed SEM images of the PP and PTFE sides can be found in Figures S8 and S9.

For further CO_2 electrolyzer operations, the reactor must be adjusted such that there is electrical contact between the cathode current collector and the CL, which is coated on the non-conductive PTFE substrate. In order to achieve this, we placed a titanium seal on top of the GDE while it was in contact with the cathode frame (Figure 3D) This titanium seal replaced the previously used Viton GDE gasket (part 6 in Figure S10). Results of the slightly modified reactor assembly are shown in Figures 3E–3H. Under a pure CO_2 feed stream, the carbon-based Sigracet 39BB-Ag GDE performed slightly better (FE_{CO} = 95.3% ± 1.4%) than the PTFE-Ag GDE (FE_{CO} = 85.2% ± 4.6%) (Figure 3E). However, when only 3% O_2 was present in the gas stream, the PTFE-Ag GDE outperformed the 39BB-Ag with an FE_{total} = 75.6% ± 8.2%. Compared with a >50% FE loss for 39BB-Ag, this demonstrates only a ∼25% FE loss due to the ORR (Figure 3F). Even at 5% O_2 in the feed, the CO_2RR remained the dominant reaction, and the ORR accounted for only ∼38% FE, whereas the ORR was responsible for ∼90% FE with 39BB-Ag. When the oxygen concentration increased even further to 10% O_2 in CO_2, thereby exceeding the typical concentration in flue gases, the 39BB-Ag failed to produce any CO_2RR products (Figure 3H). The potential shifted to a less negative value of −1.04 ± 0.05 V (lower overpotential) as a result of the dominant ORR, in contrast to the −1.41 ± 0.03 V observed for the PTFE-Ag, where the system still achieved >30% FE to CO_2RR products. The main reason why polymer-based GDEs perform better with O_2 impurities than carbon-based GDEs is the avoidance of the ORR on the substrate and, consequently, the different amount of active sites for the ORR. The use of a polymer-based GDE prevents all ORRs on the substrate because of the non-conductive nature of PP and PTFE, which inherently avoid any electron transfer and thus prevent the ORR from occurring on the substrate. The only region with competition between the CO_2RR and ORR in polymer-based GDEs is the CL (Figure 4). Furthermore, since the bare carbon-based GDL is capable of facilitating the ORR without contributing to the CO_2RR (Figure 2D), a carbon-based GDE could contain active sites for the ORR within the carbon-based substrate itself. To investigate this, we carried out electrochemical impedance spectroscopy (EIS) on both the carbon- and polymer-based substrates to determine the double-layer capacitance (C_{dl}) and estimate the electrochemically active surface area (EASA) of both configurations (Figure S10). The calculation indicates a greater EASA for the carbon-based GDE, supporting

CellPress
OPEN ACCESS

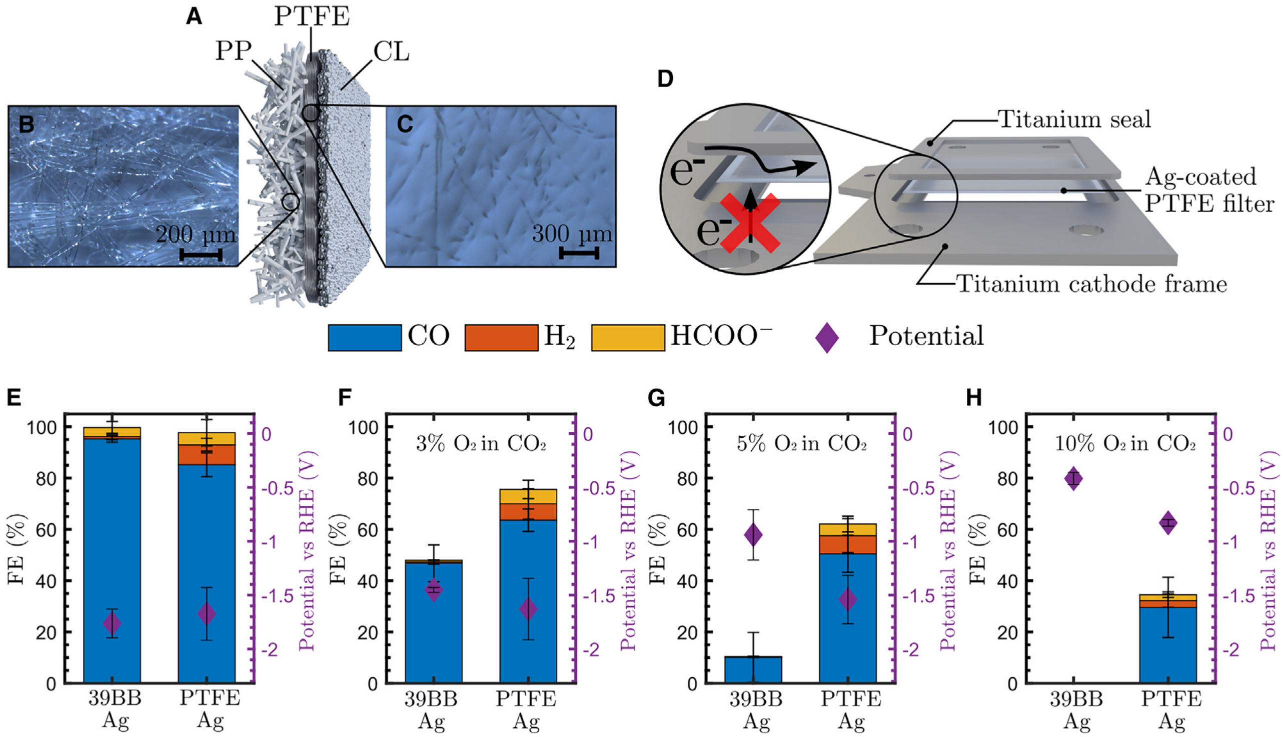

Figure 3. Ag-coated PTFE GDEs for CO_2 electrolysis in the presence of O_2

(A) Illustration of the PTFE GDE containing a polypropylene backer, a 0.2 μm porous PTFE filter, and a catalyst layer.

(B) Microscopic image of the polypropylene backer.

(C) Microscopic image of the PTFE filter layer.

(D) Assembly of the PTFE GDE in the cathode frame to provide electrical contact between the catalyst layer and the current collector.

(E–H) Ag-coated carbon and PTFE GDEs at 100 mA cm^{-2} with 0% (E), 3% (F), 5% (G), and 10% (H) O_2 in the CO_2 feed stream.

Error bars represent the standard deviation from three measurements.

the proposed hypothesis. For completeness, all data for the PTFE-Ag GDE run can be found in Figure S11.

PTFE-filter-membrane GDE for $HCOO^-$ production

Using Ag as a catalyst for CO production on polymer-based GDEs has the benefit of being sufficiently conductive by itself, whereas Bi_2O_3 is reported to be a highly resistive semiconductor.[50] As an initial test, we placed a Bi_2O_3-coated PTFE GDE in the reactor and set it for a 100 mA cm^{-2} run. Two independent measurements showed a FE_{HCOO^-} of only 23.8% ± 11.5% accompanied by high potentials (−5.57 ± 0.60 V vs. reversible hydrogen electrode [RHE]) and unreacted Bi_2O_3 areas on the GDE after disassembly as a result of insufficient conductivity (Figure S12). We propose two different GDE configurations to resolve this issue. The first configuration employs a conductive sublayer (1 mg cm^{-2} Ag) between the PTFE substrate and the Bi_2O_3 CL (2 mg cm^{-2}), as illustrated in Figure 5A. This conductive sublayer allows for an excellent distribution of electrons to the Bi_2O_3 CL but can cause undesired electrochemical side reactions (e.g., producing CO or facilitating the ORR). A second configuration is inspired by solar panels, which use conductive busbars to allow electrons to flow freely from each individual photovoltaic cell.[51] In this GDE design, 7 × 2-mm-wide conductive lines of Ag are coated on top of the Bi_2O_3 CL to provide electron highways from the catholyte-facing side without fully covering the CL (Figure 5B). We modeled the potential distributions of these two GDE configurations in COMSOL Multiphysics (electric-current module) and compared them with the failing base case of a solely Bi_2O_3-coated PTFE GDE (more information is provided in the supplemental methods and Figures S13 and S14). The simulations indicated that a conductive sublayer (Figure 5C) or busbar design (Figure 5D) is a potentially valid solution to resolve the lack of conductivity. We compared the resistance of bare Bi_2O_3-coated PTFE with that of the proposed designs through EIS measurements (Figure S15) and thus verified the feasibility of both configurations. We aimed to use only conductive materials that also act as catalysts for the CO_2RR because otherwise, the ORR-active region would increase without increasing the active sites for the CO_2RR.

Figures 5E–5H compare the performance of the carbon-based 39BB-Bi_2O_3 GDE with that of the two PTFE GDE configurations mentioned above (pictures of these prepared GDEs can be found in Figure S16). For the pure CO_2 feed, both the polymer- and carbon-based GDE substrates gave a high (>85%) FE to $HCOO^-$, experimentally proving that both the sublayer and the busbar GDE designs are suitable configurations. Surprisingly, although both GDEs contained Ag, the formation of CO remained very limited (FE_{CO} < 8%). A recent report does indeed suggest

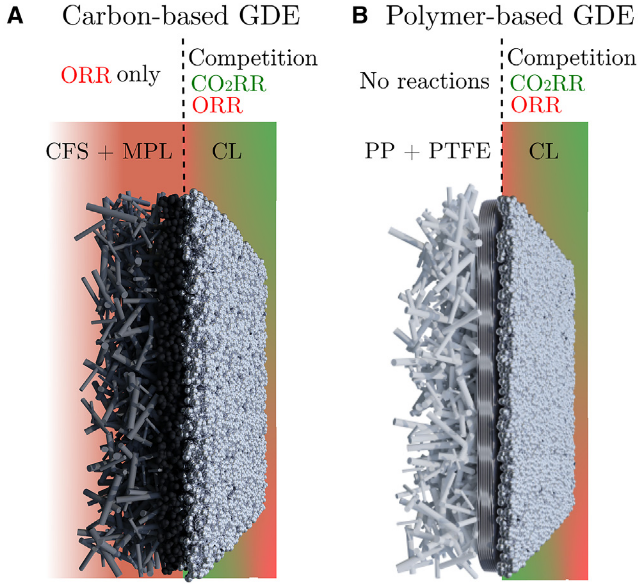

Figure 4. Schematical representation of ORR-active regions in both carbon- and polymer-based GDEs
(Left) Carbon GDE.
(Right) PTFE GDE.

that Ag-Bi_2O_3 electrocatalysts can reach >90% FE_{HCOO^-} at current densities up to 250 mA cm^{-2}.[52] Even when we replaced the Ag sublayer with carbon black (Figure S17), the FEs remained similar to those of the original PTFE-Bi_2O_3 GDEs with a Ag sublayer, except that the carbon-black sublayer caused a higher overpotential because its conductivity is lower than that of Ag. Only the potential for the PTFE-Bi_2O_3 busbar design (−2.02 ± 0.10 V vs. RHE) differed substantially from the potentials of both 39BB-Bi_2O_3 (−1.64 ± 0.01 V vs. RHE) and PTFE-Bi_2O_3 with a sublayer (−1.68 ± 0.09 V vs. RHE). An explanation can be found in the conductivity of the different GDEs. Whereas 39BB and the PTFE-Bi_2O_3 with a sublayer have bigger surfaces of conductive materials to fully distribute electrons, the busbar design relies more on the conductivity of the Bi_2O_3 and suffers from higher electrical resistances, as seen in the simulation of this design (Figure 5D). Apart from the difference in potential, both PTFE-based GDEs achieved similar product selectivities across all measurements (Figures S18 and S19). When the feed contained a mixture of CO_2 and 3% O_2 (Figure 5F), the polymer-based GDEs clearly outperformed the carbon-based ones such that the FE_{HCOO^-} remained above 70%, compared with 37.3% ± 7.5% for 39BB-Bi_2O_3. This trend continued at 5% O_2, where the FE_{HCOO^-} was only ~11% for the 39BB substrate but a remarkable ~60% for the PTFE GDE designs (Figure 5G). Even with 10% O_2 in the CO_2 feed stream, where all FEs for 39BB-Bi_2O_3 were attributed to the ORR, PTFE-Bi_2O_3 with a sublayer or busbars still reached ~30% FE to the target product, $HCOO^-$ (Figure 5H). These results demonstrate that despite the low conductivity of the Bi_2O_3 coating, it is possible to engineer a $HCOO^-$-producing polymer-based GDE, enabling an efficient CO_2RR in the presence of O_2 contaminants.

Influence of the balance gas

When we switched the balance gas from CO_2 to N_2 to examine the effect of different O_2 concentrations in another medium (Figures S20–S26), all GDE types (39BB-Ag, 39BB-Bi_2O_3, bare 39BB, bare Ti, PTFE-Ag, PTFE-Bi_2O_3 with sublayer, and PTFE-Bi_2O_3 with busbars) showed FE values similar to those under their CO_2 equivalent conditions. However, a different potential was naturally observed because the CO_2RR cannot occur when the feed stream consists of N_2, and therefore, the HER is the main reaction. The fact that the trends and total FEs were similar between CO_2 and N_2 allows us to conclude that the ORR in this work does not depend notably on the type of balance gas used.

Stability assessment and industrially relevant conditions

The advantage of using polymer-based GDEs for CO_2RR application from flue gas sources raises the question of whether these types of GDEs are suitable for long-term CO_2 electrolyzer operation in the presence of O_2 contaminants. To assess stability, we subjected each polymer-based GDE to a 50 h measurement at 100 mA cm^{-2} with 3% O_2 in the CO_2 feed stream. As stated in the methods section, we fixed the total gas flow rates at 100 mL min^{-1} and pumped electrolytes at 5 mL min^{-1} to the electrolyzer in single-pass mode to ensure the most stable reactor operating conditions. The results of the stability measurements are shown in Figure 6. For the PTFE-Ag GDE, Figure 6A reveals that over the course of 50 h, an average FE_{CO} of 61.13% was achieved at a potential of −1.59 V vs. RHE without any notable sort of degradation. Inductively coupled plasma mass spectrometry (ICP-MS) analysis of the catholyte outflow at the end of the measurement (Table S15) did not detect any Ag, indicating that the coating remained attached to the substrate. Secondly, the PTFE-Bi_2O_3 with a conductive sublayer (Figure 6B) showed a steady potential at an average value of −1.66 V vs. RHE but a FE_{HCOO^-} > 60% for the entire measurement. Lastly, relative to the aforementioned designs, the PTFE-Bi_2O_3 busbar design (Figure 6C) showed an outstanding average FE_{HCOO^-} of 71.77% despite the 3% O_2 in the feed but at the cost of a higher potential (−1.96 V vs. RHE) due to the increased resistance from the design. It is worth mentioning that, after disassembly, the PP side facing the gas chamber of the PTFE-Bi_2O_3 busbar design exhibited specific rows with water droplets (Figure S27). These rows align with the busbars on the catholyte side, which indicates that electrolyte seepage through the GDE, known as perspiration,[53] occurred for the majority through these busbars. This observation leads to the hypothesis that perspiration is now guided through the highly conductive busbars as a result of the electrowetting effect, creating specific zones for salt removal while leaving the catalytic Bi_2O_3 zones dry and open for efficient CO_2 gas diffusion. Furthermore, ICP-MS results indicated extremely limited detachment of Bi_2O_3 particles in all measurements (<2 × 10^{-2} ppm). Consequently, all polymer-based GDEs were assessed as stable, confirming the excellent stability of previously reported PTFE GDEs with pure-CO_2-fed electrolyzers.[35,47]

Previous work with solely carbon-based GDEs has shown that the FE losses by the ORR become lower when the electrolyzer

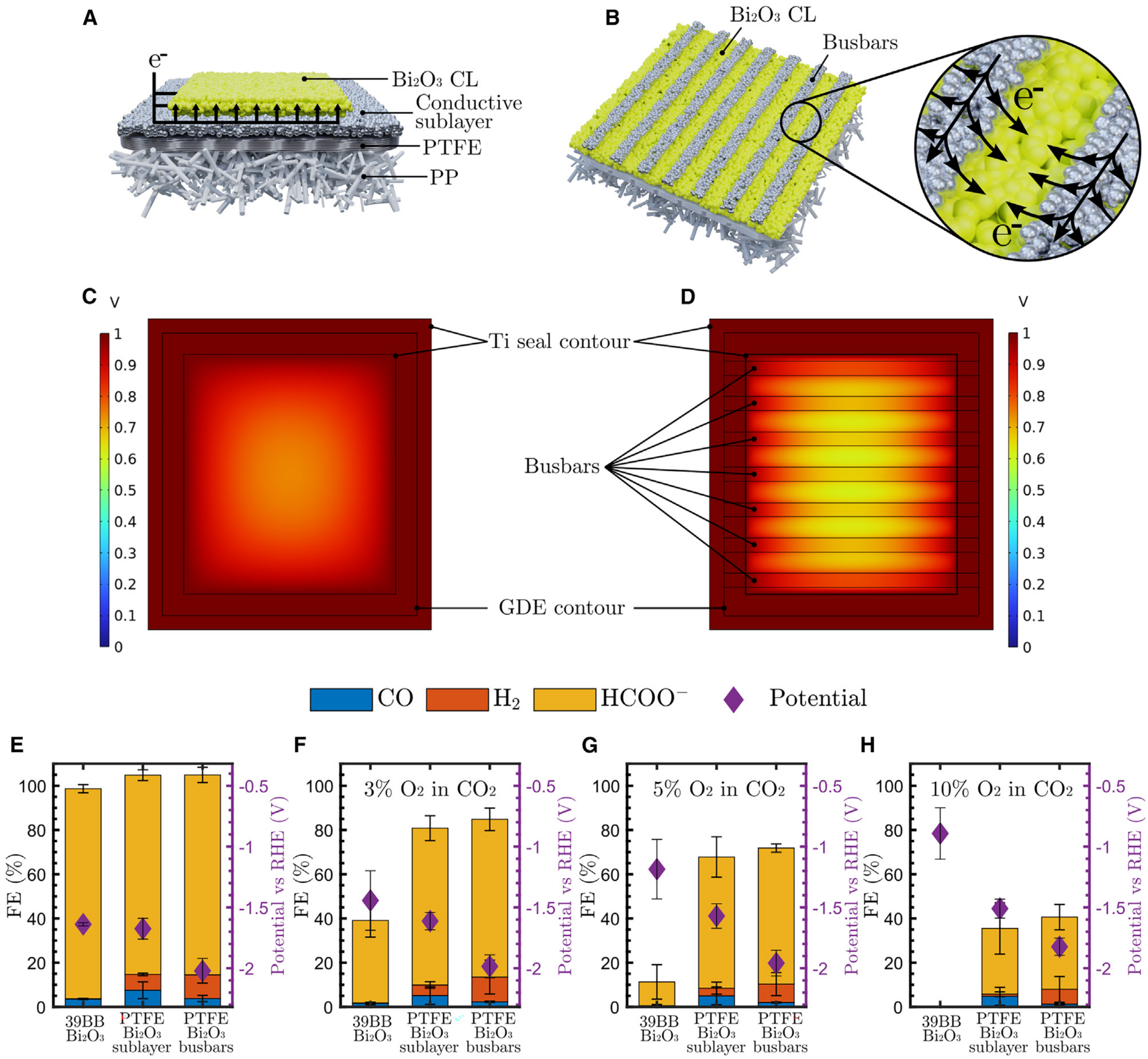

Figure 5. Illustrations and results for the $HCOO^-$-producing PTFE GDEs

(A) Schematic illustration of the PTFE-Bi_2O_3 GDE with a conductive sublayer.

(B) Schematic illustration of the PTFE-Bi_2O_3 with busbars.

(C) Potential distribution on the PTFE-Bi_2O_3 GDE with a conductive sublayer.

(D) Potential distribution on the PTFE-Bi_2O_3 GDE with busbars.

(E–H) FE for Bi_2O_3-coated carbon and PTFE GDEs at 100 mA cm^{-2} with (E) 0%, (F) 3%, (G) 5%, and (H) 10% O_2 in the CO_2 feed stream. Error bars represent the standard deviation from three measurements.

operates at >100 mA/cm^2 given that the O_2 supply to the electrode becomes mass transport limited.[26] Therefore, we conducted experiments up to 300 mA/cm^2 with a 3% O_2 feed stream, and the results for the three GDE configurations are shown in Figure S28. In line with previous work, the total FE increased at higher CD because the O_2 supply was mass transport limited, giving room for the desired CO_2RR until the parasitic H_2 evolution reaction broke through as a result of the higher operating potential and lowered CO_2 availability (more consumption) near the electrode surface. Figure S29 reveals the PTFE-Bi_2O_3 sublayer as the best configuration in terms of productivity because it reached a partial CD to C-products of 220 ± 25.0 mA/cm^2 when a total CD of 300 mA/cm^2 was applied. In terms of activity, the PTFE-Ag GDE held the lowest potentials across all the different polymer-based GDE configurations. Lastly, the polymer-based GDEs were tested with a simulated flue gas (15% CO_2 + 4% O_2 in N_2), in agreement with other literature reports.[20,46,54] Because of the low (15%) CO_2 content, the GDEs

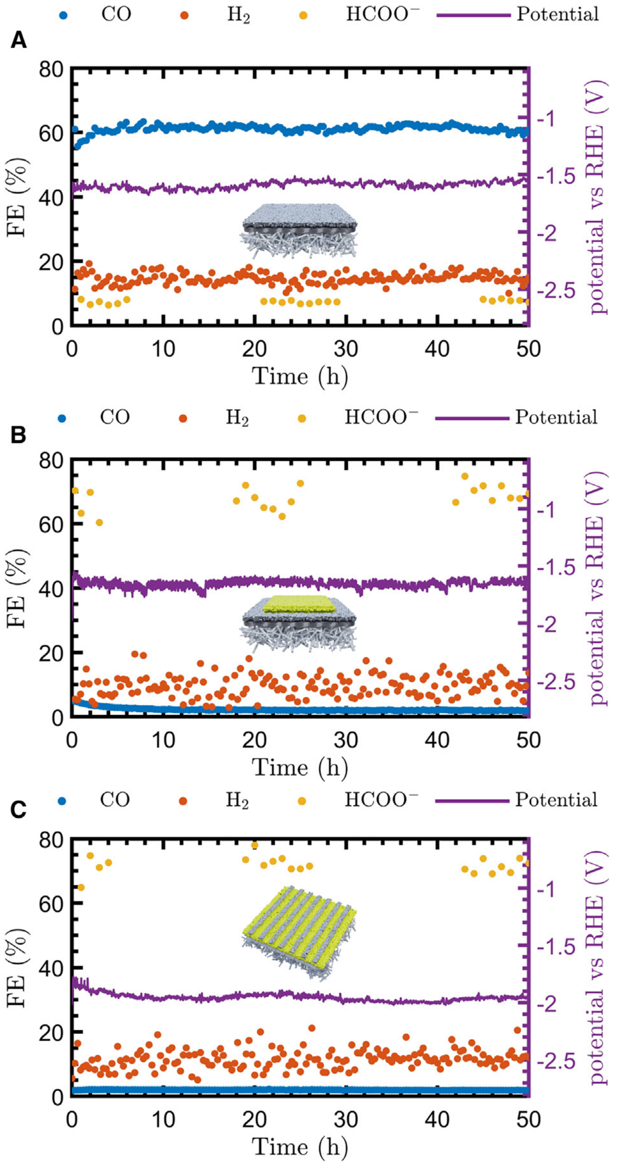

Figure 6. Stability measurements of 50 h with 3% O_2 in the CO_2 feed stream at 100 mA cm^{-2} for the polymer-based GDEs

(A) Results of the PTFE-Ag GDE.

(B) Results of the PTFE-Bi_2O_3 GDE with a conductive sublayer.

(C) Results of the PTFE-Bi_2O_3 GDE with busbars.

were tested at current densities of 50–150 mA/cm^2 given that a CO_2 shortage at the electrode prevents efficient operation. In fact, Figure S30 shows that the combination of diluted CO_2 with O_2 poses great challenges to maintaining good product selectivity. Nevertheless, FE losses of the ORR remained similar to those of the concentrated CO_2 feed stream with O_2; therefore, the O_2 tolerance of the polymer-based GDEs remained unaffected, yet future work should focus on enhancing CO_2 mass transport to the active sites with simulated flue gas feed streams. From all polymer-based GDE configurations, the PTFE-Bi_2O_3 with a conductive sublayer performed best in terms of productivity and beat the activity of the PTFE-Ag for potentials lower than −1.82 V vs. RHE (Figure S31). However, the partial CD for the CO_2RR products remained lower than 30 mA/cm^2 and should be a priority for improvement in future work.

Conclusions

Polymer-based GDEs offer a promising alternative to carbon-based GDEs, especially when O_2 is present as a contaminant in CO_2 feed streams. This study shows that the GDE substrate plays a major role in FE losses when O_2-containing CO_2 feed streams are supplied to the electrolyzer. Conventional carbon-based GDEs can facilitate the ORR even when no metal catalyst is coated on the substrate, resulting in a total product FE < 20% for feed streams containing 5% O_2. The transition to a non-conductive polymer-based GDE improves the FE to CO_2RR products (CO or $HCOO^-$) by >40% (5% O_2 in CO_2). Importantly, all three polymer-based GDE configurations show a stable performance over the course of 50 h, thus confirming their potential usage in long-term electrolyzer operation. Furthermore, increasing the productivity for the CO_2RR to CO or formate from a low concentrated CO_2 feed containing O_2 impurities poses a great challenge to the direct utilization of waste CO_2 streams. The insights from this study can be used in the design of a CO_2 electrolysis process that uses impure CO_2 sources to create valuable chemicals. Omitting or reducing the necessary separation and purification steps is of tremendous interest because it would make CO_2 electrolyzer technologies more economically viable.

METHODS

Chemicals and products

We made the electrolytes by dissolving potassium bicarbonate (>99.5%, Chem-lab) or potassium hydroxide (>85%, Chem-lab) in ultrapure water (Milli-Q, Millipore; resistivity: 18.2 MΩ cm) to obtain the desired concentration of reagents (0.5 M $KHCO_3$ and 1 M KOH). Perchloric acid (70%, Chem-lab) was diluted with ultrapure water to a 1.2 M solution and used during high-performance liquid chromatography (HPLC) sample preparation. We used sulfuric acid (>98%, Chem-lab) to prepare the 10 mM H_2SO_4 mobile phase for HPLC analysis and used nitric acid (70%, Chem-lab) to prepare samples for ICP-MS. Hydrogen peroxide (30%, VWR) was used during activation of the Nafion 117 cation-exchange membrane (Ion Power). We used commercial bismuth oxide nanoparticles (99.8%, 90–210 nm, Sigma-Aldrich), silver nanoparticles (99.5%, <100 nm, Sigma-Aldrich), or carbon black (Vulcan XC72, Nanografi) with isopropanol (99.8%, Chem-lab), ultrapure water, and a Nafion dispersion (D520, 5% w/w in water, and 1-propanol, Thermo Fisher Scientific) to prepare the catalyst inks. Transmission electron microscopy images and X-ray diffraction measurements of the Ag and Bi_2O_3 catalytic nanoparticles can be found in Figures S32 and S33. Ethanol (>99.9%, Chem-lab), acetone (>99.8%, Chem-lab), and a 60% (w/w) PTFE dispersion in water (Sigma-Aldrich) were used during the sintering procedure. Helium (99.999%, Air Liquide) was used as carrier gas for the GC, and argon (99.999%, Air Liquide) was used for spray coating. CO_2 (99.998%, Air Liquide), an O_2/CO_2 mixture (19.96% O_2 in CO_2,

Nippon gases), a simulated flue gas (15% CO_2 + 4% O_2 in N_2, Air Liquide), and air (20% ± 1% O_2 in N_2, Air Liquide) were used during experiments.

GDEs

We made the catalyst ink solutions by mixing 72 mg of nanoparticles (Ag or Bi_2O_3) with 200 mg (5% w/w) Nafion dispersion, isopropanol, and ultrapure water unless stated otherwise. The volumetric isopropanol/water ratios were 4:1 and 2:1 for Bi_2O_3 and Ag, respectively, for a total volume of 6 mL. Inks were sonicated for at least 30 min with a 6 mm titanium probe (NexT-gen Lab120 at 34 kHz with an 84 μm amplitude). Using a Fengda FE-186K airbrush with argon as the carrier gas, we spray coated the inks onto the GDLs, which were fixed on a hot plate (80°C). We used two main GDLs as the substrates for the catalyst inks: the carbon-based Sigracet GDL 39BB (purchased from Ion Power) and the polymer-based Aspire laminated hydrophobic PTFE filter with 0.2 μm pores (purchased from Sterlitech), each with a geometrical active surface area of 10 cm^2. Their specific properties are listed in Tables S16 and S17, respectively. We weighed the GDEs before and after spray coating to ensure a catalyst loading of 2 mg cm^{-2}. In the PTFE-Bi_2O_3 configuration with a conductive sublayer or busbar design, we spray coated the second CL by using a PMMA mask with a smaller rectangle than the original layer or a busbar-shaped pattern. For experiments mentioning Ti felt, we used a 50%–56% porosity Ti fiber felt (purchased from Fuel Cell Store) after pretreating it to increase its hydrophobic properties such that it was able to maintain a gas-liquid boundary during operation. The pretreatment was adapted from the methods of Omrani and Shabani[55] and consisted of (1) cleaning the Ti felt with acetone, ethanol, and distilled water; (2) drying it at 120°C for 30 min; (3) dipping it in a 25% (w/w) PTFE emulsion; (4) drying it at 120°C for 60 min; and (5) sintering it at 340°C under argon atmosphere for 60 min.

Flow-reactor operation

Similar to previous reports,[21,56,57] this study used a modified ElectroCell Micro Flow Cell (Figure S34) with a Ag/AgCl reference electrode (Innovative Instruments). Viton gaskets and PMMA flow plates were fabricated in house with a CNC mill (Euromod MP45). The Nafion 117 cation-exchange membrane separated the catholyte and anolyte compartments. To enhance its ionic conductivity and remove organic contaminants, we pretreated the membrane by sequentially boiling it in the following solutions: 3% H_2O_2 (1 h), distilled water (1 h), 1 M H_2SO_4 (1 h), and distilled water again (1 h). After pretreatment, the membrane was stored at 4°C until further use.

In the experimental setup, a peristaltic pump (Shenchen LabS3, Drifton) supplied the cathode compartment with 5 mL min^{-1} 0.5 M $KHCO_3$. On the anode side, the pump supplied a 1 M KOH solution with the same flow rate to facilitate the oxygen evolution reaction. Both electrolytes were pumped through the cell in single-pass mode, ensuring stable and reliable conditions during the experiments. The gas feed stream was controlled with Analyt-MTC mass flow controllers. We obtained different feed-gas compositions by mixing 20% O_2 in CO_2 with pure CO_2 in different volumetric ratios. We conducted electrochemical measurements with a potentiostat (PGSTAT302N, Metrohm) and a 10 A booster (Metrohm) and non-iR corrected the reported working-electrode potentials. The potentials were converted to RHE through the equation $E_{RHE} = E_{Ag/AgCl} + E^0_{Ag/AgCl} + 0.058 \times \text{pH}$, where $E^0_{Ag/AgCl} = 0.197$ V and pH = 8.36. Typically, we operated the reactor for 25 min at a given condition, took a liquid sample from the catholyte outflow, and then injected a gas sample into the in-line GC before switching to the next operating condition. We conducted EIS by using the potentiostat's frequency response analyzer (FRA) module to obtain the C_{dl} and estimate the EASA as EASA = $\frac{C_{dl}}{C_s}$ by using a specific charge density of $C_s = 40\ \mu F/cm^2$.[58]

Product analysis and imaging

We acidified 1 mL of liquid catholyte sample with 1 mL of 1.2 M $HClO_4$. After acidification, we vortexed the samples and filtered them with a 0.2 μm filter to prepare them for analysis. The analysis was performed on an HPLC system (Alliance 2695) equipped with a Shodex RSpak KC811 column and a PDA detector (210 nm, Waters). We injected the acidified and filtered samples into the column and compared them with a 1,000 ppm standard to quantify the amount of $HCOO^-$ produced during reactor operation. For accurate calculation of FE, we determined the real liquid flow rate by monitoring the weight of the catholyte reservoir. After the 50 h stability measurements, we diluted a catholyte sample in 1% nitric acid and measured it with ICP-MS (Agilent 7500 Series).

The gaseous reactor outlet was directly connected to a GC (Shimadzu) equipped with a Restek Shincarbon ST column (1 mm internal diameter, 2 m length, and mesh 100/120) for product analysis and helium as carrier gas. The product analysis began at a temperature of 40°C and continued for 3 min. After that, the temperature increased linearly at a rate of 40°C per minute until it reached 250°C. The thermal conductivity detector of the GC remained at a constant temperature of 280°C throughout the analysis. For precise product quantification, the outgoing gas flow rate was measured with a volumetric flow meter (Restek ProFlow 6000).

Microscopic GDE images were obtained with an M165 C light microscope (Leica Microsystems). SEM images were performed on the GDE samples with a Thermo Fischer Scientific Quanta FEI 250 microscope operated at an accelerating voltage of 20 kV.

RESOURCE AVAILABILITY

Lead contact

Requests for further information and resources should be directed to and will be fulfilled by the lead contact, Tom Breugelmans (tom.breugelmans@uantwerpen.be).

Materials availability

This study did not generate new unique materials.

Data and code availability

- The (processed) data are freely available from the Zenodo repository of the University of Antwerp and Applied Electrochemistry and Catalysis (ELCAT) Research Group: https://zenodo.org/communities/uantwerp-elcat/.
- This paper does not report original code.
- Any additional information required to reanalyze the data reported in this paper is available from the lead contact upon request.

ACKNOWLEDGMENTS

This work was funded by the European Union for actions 101088063 - TRANSCEND and 101092257 - THREADING-CO2. The views and opinions expressed are, however, those of the authors only and do not necessarily reflect those of the European Union or the European Research Council Executive Agency (ERCEA). Neither the European Union nor the ERCEA can be held responsible for them. This project was co-funded by the Flanders Industry Innovation Moonshot program via grant CAPTIN II HBC.2021.0255. The authors thank Max Van Brusselen for performing the ICP-MS measurements, as well as Kavita Shivanagoud Patil and Brend De Coen for acquiring the SEM images.

AUTHOR CONTRIBUTIONS

Conceptualization, S.V.D., L.H., and D.C.; investigation, S.V.D. and L.H.; visualization, S.V.D.; writing – original draft, S.V.D.; writing – review & editing, L.H., D.C., and T.B.; supervision, D.C., N.D., J.H., and T.B.; project administration, N.D., J.H., and T.B.; funding acquisition, N.D., J.H., and T.B.

DECLARATION OF INTERESTS

The authors declare no competing interests.

SUPPLEMENTAL INFORMATION

Supplemental information can be found online at https://doi.org/10.1016/j.checat.2025.101353.

Received: October 24, 2024
Revised: December 6, 2024
Accepted: March 14, 2025
Published: April 10, 2025

REFERENCES

1. Intergovernmental Panel on Climate Change (2021). Climate change 2021: the physical science basis. In IPCC Sixth Assessment Report, V. Masson-Delmotte, P. Zhai, A. Pirani, S.L. Connors, C. Péan, S. Berger, N. Caud, Y. Chen, L. Goldfarb, and M. Gomis, eds.
2. Overa, S., Feric, T.G., Park, A.-H.A., and Jiao, F. (2021). Tandem and hybrid processes for carbon dioxide utilization. Joule *5*, 8–13. https://doi.org/10.1016/j.joule.2020.12.004.
3. De Luna, P., Hahn, C., Higgins, D., Jaffer, S.A., Jaramillo, T.F., and Sargent, E.H. (2019). What would it take for renewably powered electrosynthesis to displace petrochemical processes? Science *364*, eaav3506. https://doi.org/10.1126/science.aav3506.
4. Chen, C., Khosrowabadi Kotyk, J.F., and Sheehan, S.W. (2018). Progress toward commercial application of electrochemical carbon dioxide reduction. Chem *4*, 2571–2586. https://doi.org/10.1016/j.chempr.2018.08.019.
5. Hintjens, L., Van Daele, S., Schalck, J., Vranckaert, M., Neukermans, S., Choukroun, D., and Breugelmans, T. (2024). Unravelling the key role of ion-exchange membranes in water management and ion crossover for zero-gap CO_2 electrolyzers. J. Mater. Chem. A Mater. *12*, 25086–25099. https://doi.org/10.1039/D4TA02614D.
6. Tan, Y.C., Lee, K.B., Song, H., and Oh, J. (2020). Modulating local CO_2 concentration as a general strategy for enhancing C-C coupling in CO_2 electroreduction. Joule *4*, 1104–1120. https://doi.org/10.1016/j.joule.2020.03.013.
7. Nesbitt, N.T., Burdyny, T., Simonson, H., Salvatore, D., Bohra, D., Kas, R., and Smith, W.A. (2020). Liquid-solid boundaries dominate activity of CO_2 reduction on gas-diffusion electrodes. ACS Catal. *10*, 14093–14106. https://doi.org/10.1021/acscatal.0c03319.
8. Burdyny, T., and Smith, W.A. (2019). CO_2 reduction on gas-diffusion electrodes and why catalytic performance must be assessed at commercially-relevant conditions. Energy Environ. Sci. *12*, 1442–1453. https://doi.org/10.1039/C8EE03134G.
9. Wu, Y., Garg, S., Li, M., Idros, M.N., Li, Z., Lin, R., Chen, J., Wang, G., and Rufford, T.E. (2022). Effects of microporous layer on electrolyte flooding in gas diffusion electrodes and selectivity of CO_2 electrolysis to CO. J. Power Sources *522*, 230998. https://doi.org/10.1016/j.jpowsour.2022.230998.
10. Wakerley, D., Lamaison, S., Wicks, J., Clemens, A., Feaster, J., Corral, D., Jaffer, S.A., Sarkar, A., Fontecave, M., Duoss, E.B., et al. (2022). Gas diffusion electrodes, reactor designs and key metrics of low-temperature CO_2 electrolysers. Nat. Energy *7*, 130–143. https://doi.org/10.1038/s41560-021-00973-9.
11. Jeanty, P., Scherer, C., Magori, E., Wiesner-Fleischer, K., Hinrichsen, O., and Fleischer, M. (2018). Upscaling and continuous operation of electrochemical CO_2 to CO conversion in aqueous solutions on silver gas diffusion electrodes. J. CO_2 Util. *24*, 454–462. https://doi.org/10.1016/j.jcou.2018.01.011.
12. Schweiss, R., Meiser, C., Damjanovic, T., Galbiati, I., and Haak, N. (2016). SIGRACET gas diffusion layers for PEM fuel cells, electrolyzers and batteries. SGL Carbon.
13. Wu, B., Voleti, L.D., Fenwick, A.Q., Wu, C., Zhang, J., Ling, N., Wang, M., Jia, Y., Tjiu, W.W., Zhang, M., et al. (2025). A reversed gas diffusion electrode enables collection of high purity gas products from CO_2 electroreduction. EES Catal. *3*, 318–326. https://doi.org/10.1039/D4EY00253A.
14. Verma, S., Hamasaki, Y., Kim, C., Huang, W., Lu, S., Jhong, H.-R.M., Gewirth, A.A., Fujigaya, T., Nakashima, N., and Kenis, P.J.A. (2017). Insights into the low overpotential electroreduction of CO_2 to CO on a supported gold catalyst in an alkaline flow electrolyzer. ACS Energy Lett. *3*, 193–198. https://doi.org/10.1021/acsenergylett.7b01096.
15. Fan, L., Xia, C., Zhu, P., Lu, Y., and Wang, H. (2020). Electrochemical CO_2 reduction to high concentration pure formic acid solutions in an all-solid-state reactor. Nat. Commun. *11*, 3633. https://doi.org/10.1038/s41467-020-17403-1.
16. Chen, Y., Vise, A., Klein, W.E., Cetinbas, F.C., Myers, D.J., Smith, W.A., Deutsch, T.G., and Neyerlin, K.C. (2020). A robust, scalable platform for the electrochemical conversion of CO_2 to formate: identifying pathways to higher energy efficiencies. ACS Energy Lett. *5*, 1825–1833. https://doi.org/10.1021/acsenergylett.0c00860.
17. Baumgartner, L.M., Koopman, C.I., Forner-Cuenca, A., and Vermaas, D.A. (2022). Narrow pressure stability window of gas diffusion electrodes limits the scale-up of CO_2 electrolyzers. ACS Sustain. Chem. Eng. *10*, 4683–4693. https://doi.org/10.1021/acssuschemeng.2c00195.
18. Lee, M.Y., Park, K.T., Lee, W., Lim, H., Kwon, Y., and Kang, S. (2020). Current achievements and the future direction of electrochemical CO_2 reduction: a short review. Crit. Rev. Environ. Sci. Technol. *50*, 769–815. https://doi.org/10.1080/10643389.2019.1631991.
19. Kolster, C., Mechleri, E., Krevor, S., and Mac Dowell, N. (2017). The role of CO_2 purification and transport networks in carbon capture and storage cost reduction. Int. J. Greenh. Gas Control *58*, 127–141. https://doi.org/10.1016/j.ijggc.2017.01.014.
20. Al-Attas, T., Nabil, S.K., Zeraati, A.S., Shiran, H.S., Alkayyali, T., Zargartalebi, M., Tran, T., Marei, N.N., Al Bari, M.A., Lin, H., et al. (2023). Permselective MOF-based gas diffusion electrode for direct conversion of CO_2 from quasi flue gas. ACS Energy Lett. *8*, 107–115. https://doi.org/10.1021/acsenergylett.2c02305.
21. Van Daele, S., Hintjens, L., Van den Hoek, J., Neukermans, S., Daems, N., Hereijgers, J., and Breugelmans, T. (2022). Influence of the target product on the electrochemical reduction of diluted CO_2 in a continuous flow cell. J. CO_2 Util. *65*, 102210. https://doi.org/10.1016/j.jcou.2022.102210.
22. Kim, B., Seong, H., Song, J.T., Kwak, K., Song, H., Tan, Y.C., Park, G., Lee, D., and Oh, J. (2020). Over a 15.9% solar-to-co conversion from dilute CO_2 streams catalyzed by gold nanoclusters exhibiting a high CO_2 binding affinity. ACS Energy Lett. *5*, 749–757. https://doi.org/10.1021/acsenergylett.9b02511.

CellPress
OPEN ACCESS

23. Luc, W., Ko, B.H., Kattel, S., Li, S., Su, D., Chen, J.G., and Jiao, F. (2019). SO_2-induced selectivity change in CO_2 electroreduction. J. Am. Chem. Soc. *141*, 9902–9909. https://doi.org/10.1021/jacs.9b03215.

24. Ko, B.H., Hasa, B., Shin, H., Jeng, E., Overa, S., Chen, W., and Jiao, F. (2020). The impact of nitrogen oxides on electrochemical carbon dioxide reduction. Nat. Commun. *11*, 5856.

25. Choi, B.U., Tan, Y.C., Song, H., Lee, K.B., and Oh, J. (2021). System design considerations for enhancing electroproduction of formate from simulated flue gas. ACS Sustain. Chem. Eng. *9*, 2348–2357. https://doi.org/10.1021/acssuschemeng.0c08632.

26. Van Daele, S., Hintjens, L., Hoekx, S., Bohlen, B., Neukermans, S., Daems, N., Hereijgers, J., and Breugelmans, T. (2024). How flue gas impurities affect the electrochemical reduction of CO_2 to Co and formate. Appl. Catal. B Environ. *341*, 123345. https://doi.org/10.1016/j.apcatb.2023.123345.

27. Legrand, U., Apfel, U.P., Boffito, D.C., and Tavares, J.R. (2020). The effect of flue gas contaminants on the CO_2 electroreduction to formic acid. J. CO_2 Util. *42*, 101315. https://doi.org/10.1016/j.jcou.2020.101315.

28. Ma, R., Lin, G., Zhou, Y., Liu, Q., Zhang, T., Shan, G., Yang, M., and Wang, J. (2019). A review of oxygen reduction mechanisms for metal-free carbon-based electrocatalysts. npj Comput. Mater. *5*, 78. https://doi.org/10.1038/s41524-019-0210-3.

29. An, F., Bao, X.Q., Deng, X.Y., Ma, Z.Z., and Wang, X.G. (2022). Carbon-based metal-free oxygen reduction reaction electrocatalysts: past, present and future. New Carbon Mater. *37*, 338–354. https://doi.org/10.1016/S1872-5805(22)60590-0.

30. Küngas, R. (2020). Electrochemical CO_2 reduction for co production: comparison of low-and high-temperature electrolysis technologies. J. Electrochem. Soc. *167*, 044508. https://doi.org/10.1149/1945-7111/ab7099.

31. Al-Tamreh, S.A., Ibrahim, M.H., El-Naas, M.H., Vaes, J., Pant, D., Benamor, A., and Amhamed, A. (2021). Electroreduction of carbon dioxide into formate: a comprehensive review. Chemelectrochem *8*, 3207–3220. https://doi.org/10.1002/celc.202100438.

32. Ge, X., Sumboja, A., Wuu, D., An, T., Li, B., Goh, F.W.T., Hor, T.S.A., Zong, Y., and Liu, Z. (2015). Oxygen reduction in alkaline media: from mechanisms to recent advances of catalysts. ACS Catal. *5*, 4643–4667. https://doi.org/10.1021/acscatal.5b00524.

33. Sui, R., Zhang, X., Wang, X., Wang, X., Pei, J., Zhang, Y., Liu, X., Chen, W., Zhu, W., and Zhuang, Z. (2022). Silver based single atom catalyst with heteroatom coordination environment as high performance oxygen reduction reaction catalyst. Nano Res. *15*, 7968–7975. https://doi.org/10.1007/s12274-022-4499-8.

34. He, M., Li, C., Zhang, H., Chang, X., Chen, J.G., Goddard, W.A., 3rd, Cheng, M.-j., Xu, B., and Lu, Q. (2020). Oxygen induced promotion of electrochemical reduction of CO_2 via co-electrolysis. Nat. Commun. *11*, 3844. https://doi.org/10.1038/s41467-020-17690-8.

35. Dinh, C.T., Burdyny, T., Kibria, M.G., Seifitokaldani, A., Gabardo, C.M., García de Arquer, F.P., Kiani, A., Edwards, J.P., De Luna, P., Bushuyev, O.S., and Zou, C. (2018). CO_2 electroreduction to ethylene via hydroxide-mediated copper catalysis at an abrupt interface. Science *360*, 783–787. https://doi.org/10.1126/science.aas9100.

36. Jiang, Y., Wang, X., Duan, D., He, C., Ma, J., Zhang, W., Liu, H., Long, R., Li, Z., Kong, T., et al. (2022). Structural reconstruction of Cu_2O superparticles toward electrocatalytic CO_2 reduction with high C^{2+} products selectivity. Adv. Sci. *9*, 2105292. https://doi.org/10.1002/advs.202105292.

37. Deng, B., Huang, M., Zhao, X., Mou, S., and Dong, F. (2021). Interfacial electrolyte effects on electrocatalytic CO_2 reduction. ACS Catal. *12*, 331–362. https://doi.org/10.1021/acscatal.1c03501.

38. Li, X.R., Meng, X.Z., Zhang, Q.H., Cai, H.R., Yan, Z.Z., Wu, L.K., and Cao, F.H. (2022). In situ studies of hydrogen evolution kinetics on pure titanium surface: the effects of pre-reduction and dissolved oxygen. J. Phys. Chem. C *126*, 1828–1844. https://doi.org/10.1021/acs.jpcc.1c09818.

39. Sterlitech. PTFE Filters, Aspire Laminated, Hydrophobic, Polyester Backer, 0.2 Micron, 200 x 250mm, 5/Pk. SKU 0QP9522005. https://www.sterlitech.com/0qp9522005.html.

40. Dhanumalayan, E., and Joshi, G.M. (2018). Performance properties and applications of polytetrafluoroethylene (PTFE)—a review. Adv. Compos. Hybrid Mater. *1*, 247–268. https://doi.org/10.1007/s42114-018-0023-8.

41. Li, F., Thevenon, A., Rosas-Hernández, A., Wang, Z., Li, Y., Gabardo, C.M., Ozden, A., Dinh, C.T., Li, J., Wang, Y., et al. (2020). Molecular tuning of CO_2-to-ethylene conversion. Nature *577*, 509–513. https://doi.org/10.1038/s41586-019-1782-2.

42. Wang, Y., Wang, Z., Dinh, C.T., Li, J., Ozden, A., Golam Kibria, M., Seifitokaldani, A., Tan, C.S., Gabardo, C.M., Luo, M., et al. (2019). Catalyst synthesis under CO_2 electroreduction favours faceting and promotes renewable fuels electrosynthesis. Nat. Catal. *3*, 98–106. https://doi.org/10.1038/s41929-019-0397-1.

43. García de Arquer, F.P., Dinh, C.-T., Ozden, A., Wicks, J., McCallum, C., Kirmani, A.R., Nam, D.-H., Gabardo, C., Seifitokaldani, A., Wang, X., et al. (2020). CO_2 electrolysis to multicarbon products at activities greater than 1 A cm^{-2}. Science *367*, 661–666. https://doi.org/10.1126/science.aay4217.

44. O'Brien, C.P., Miao, R.K., Liu, S., Xu, Y., Lee, G., Robb, A., Huang, J.E., Xie, K., Bertens, K., Gabardo, C.M., et al. (2021). Single pass CO_2 conversion exceeding 85% in the electrosynthesis of multicarbon products via local CO_2 regeneration. ACS Energy Lett. *6*, 2952–2959. https://doi.org/10.1021/acsenergylett.1c01122.

45. Wang, X., Xu, A., Li, F., Hung, S.F., Nam, D.H., Gabardo, C.M., Wang, Z., Xu, Y., Oz- den, A., Rasouli, A.S., et al. (2020). Efficient methane electrosynthesis enabled by tuning local CO_2 availability. J. Am. Chem. Soc. *142*, 3525–3531. https://doi.org/10.1021/jacs.9b12445.

46. Xu, Y., Edwards, J.P., Zhong, J., O'Brien, C.P., Gabardo, C.M., McCallum, C., Li, J., Dinh, C.T., Sargent, E.H., and Sinton, D. (2020). Oxygen-tolerant electroproduction of C2 products from simulated flue gas. Energy Environ. Sci. *13*, 554–561. https://doi.org/10.1039/c9ee03077h.

47. Gabardo, C.M., O'Brien, C.P., Edwards, J.P., McCallum, C., Xu, Y., Dinh, C.T., Li, J., Sargent, E.H., and Sinton, D. (2019). Continuous carbon dioxide electroreduction to concentrated multi-carbon products using a membrane electrode assembly. Joule *3*, 2777–2791. https://doi.org/10.1016/j.joule.2019.07.021.

48. Iglesias van Montfort, H.P., Li, M., Irtem, E., Abdinejad, M., Wu, Y., Pal, S.K., Sassenburg, M., Ripepi, D., Subramanian, S., Biemolt, J., et al. (2023). Non-invasive current collectors for improved current-density distribution during CO_2 electrolysis on super-hydrophobic electrodes. Nat. Commun. *14*, 6579. https://doi.org/10.1038/s41467-023-42348-6.

49. Wang, M., Wang, B., Zhang, J., Xi, S., Ling, N., Mi, Z., Yang, Q., Zhang, M., Leow, W.R., Zhang, J., and Lum, Y. (2024). Acidic media enables oxygen-tolerant electrosynthesis of multicarbon products from simulated flue gas. Nat. Commun. *15*, 1218. https://doi.org/10.1038/s41467-024-45527-1. https://www.nature.com/articles/s41467-024-45527-1.

50. Mansfield, R. (1949). The electrical properties of bismuth oxide. Proc. Phys. Soc. B *62*, 476–483. https://doi.org/10.1088/0370-1301/62/8/302.

51. Mahmoud, M., Olabi, A.G., Abdelkareem, M.A., Rabaia, M.K.H., and Sayed, E.T. (2023). Chapter 2.4 – Technical review on solar photovoltaics. In Renewable Energy – Volume 1: Solar, Wind, and Hydropower, A.G. Olabi, ed. (Academic Press), pp. 219–235. https://doi.org/10.1016/B978-0-323-99568-9.00012-1.

52. Wang, X., He, W., Shi, J., Junqueira, J.R.C., Zhang, J., Dieckhöfer, S., Seisel, S., Das, D., and Schuhmann, W. (2023). Ag-induced phase transition of Bi_2O_3 nanofibers for enhanced energy conversion efficiency towards formate in CO_2 electroreduction. Chem. Asian J. *18*, e202201165. https://doi.org/10.1002/asia.202201165.

53. Mot, B.D., Hereijgers, J., Duarte, M., and Breugelmans, T. (2019). Influence of flow and pressure distribution inside a gas diffusion electrode on the performance of a flow-by CO_2 electrolyzer. Chem. Eng. J. *378*, 12224. https://doi.org/10.1016/j.cej.2019.122224.

54. Takeda, Y., Mizuno, S., Iwata, R., Morikawa, T., and Kato, N. (2023). Gas-fed liquid-covered electrodes used for electrochemical reduction of dilute CO_2 in a flue gas. J. CO_2 Util. *71*, 102472. https://doi.org/10.1016/j.jcou.2023.102472.

55. Omrani, R., and Shabani, B. (2019). Can PTFE coating of gas diffusion layer improve the performance of URFCs in fuel cell-mode? Energy Proc. *160*, 574–581. https://doi.org/10.1016/j.egypro.2019.02.208.

56. Duarte, M., De Mot, B., Hereijgers, J., and Breugelmans, T. (2019). Electrochemical reduction of CO_2: effect of convective CO_2 supply in gas diffusion electrodes. ChemElectroChem *6*, 5596–5602. https://doi.org/10.1002/celc.201901454.

57. Duarte, M., Daems, N., Hereijgers, J., Arenas-Esteban, D., Bals, S., and Breugelmans, T. (2021). Enhanced CO_2 electroreduction with metal-nitrogen-doped carbons in a continuous flow reactor. J. CO_2 Util. *50*, 101583. https://doi.org/10.1016/j.jcou.2021.101583.

58. Cossar, E., Houache, M.S.E., Zhang, Z., and Baranova, E.A. (2020). Comparison of electro- chemical active surface area methods for various nickel nanostructures. J. Electroanal. Chem. *870*, 114246. https://doi.org/10.1016/j.jelechem.2020.114246.

Chem Catalysis, Volume *5*

Supplemental information

Promoting CO_2 reduction in the presence of oxygen with polymer-based gas diffusion electrodes

Sam Van Daele, Lieven Hintjens, Daniel Choukroun, Nick Daems, Jonas Hereijgers, and Tom Breugelmans

Supplemental Methods

The potential distribution in the GDE was modelled in COMSOL Multiphysics (Electric Currents module) with the following simplifications:

- PP and PTFE are considered perfect insulators and therefore not included in the model.
- Catalyst layers are modelled as a bulk material.
- Joule heating and general heat transfer are considered negligible.

Since modelling of the exact morphology and conductivity of spray coated ionomer-containing catalyst ink layers is extremely complex, these materials are assigned a lower conductivity than their bulk metallic value in order to approach the characteristics of the catalyst layer.

The base case consists of a 5 µm thick Bi_2O_3 CL ($\sigma = 10^3$ S m^{-1}) in contact with a 1 mm Ti frame ($\sigma = 7{\cdot}10^5$ S m^{-1}). The electrolyte is represented by a 1 cm thick rectangle ($\sigma = 10^{-1}$ S m^{-1} for a 0.5 M salt solution) that is in contact with the CL. A potential of 1 V is applied on the Ti frame as illustrated in Fig. S.13.

Other configurations are modelled in exactly the same way as the base case, but only the CL thickness and materials are changed. This includes a PTFE GDE with a 5 µm thick Ag CL ($\sigma = 10^3$ S m^{-1}) (Fig. S.14 A), a 2 µm thick Ag sublayer with a 5 µm Bi_2O_3 CL (Fig. S.14 B) and a 5 µm Bi_2O_3 CL with 7x2 cm thick Ag busbars included.

Supplemental Figures

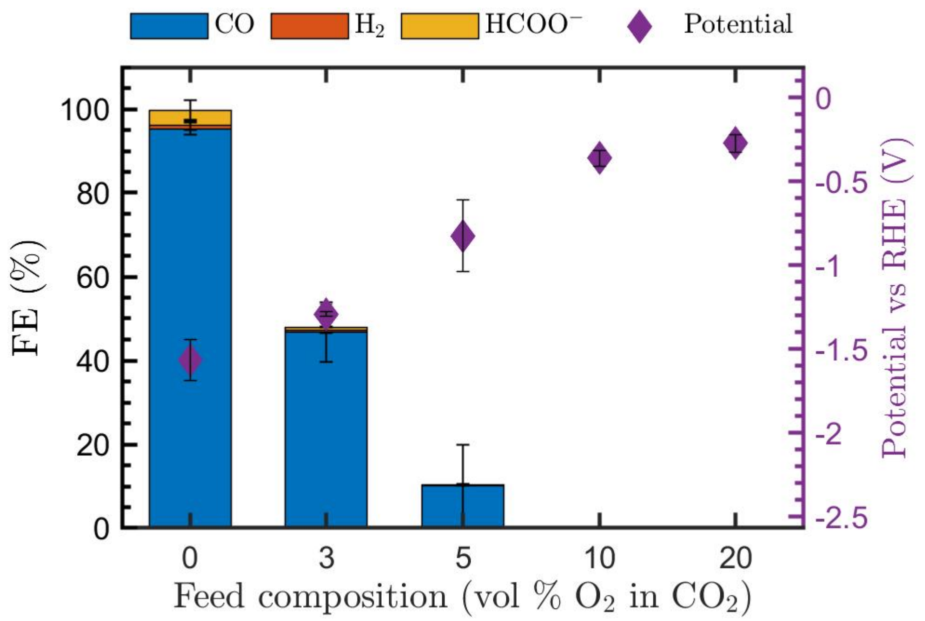

Figure S.1: All results for the carbon-based 39BB GDE with Ag nanoparticles at 100 mA cm^{-2}, related to Fig. 2.

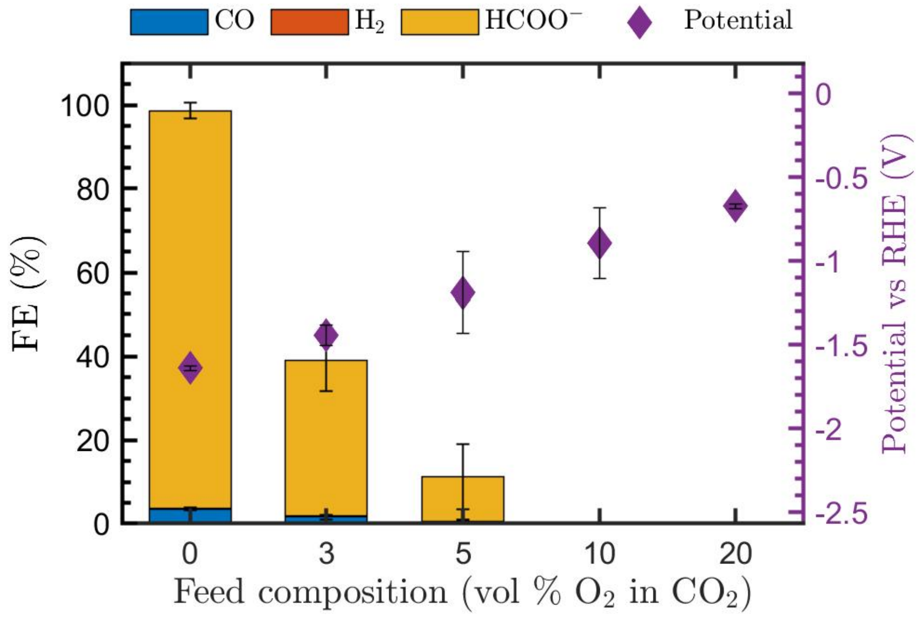

Figure S.2: All results for the carbon-based 39BB GDE with Bi_2O_3 nanoparticles at 100 mA cm^{-2}, related to Fig. 2.

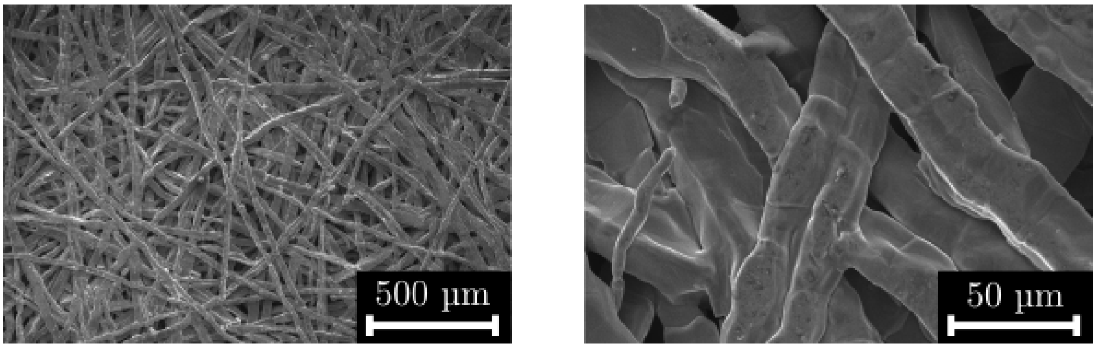

Figure S.3: SEM images of bare Ti felt at different magnifications.

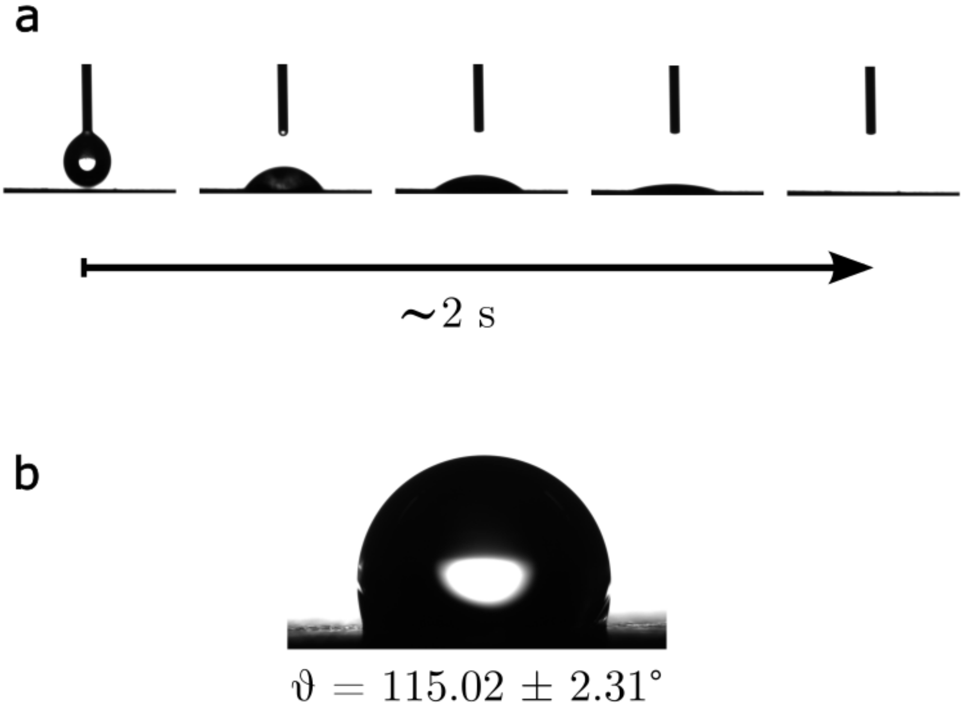

Figure S.4: Contact angle measurements on bare Ti felt. **a** Measurement of untreated Ti felt. The insufficient hydrophobicity will not provide a gas-liquid barrier in the reactor. **b** Contact angle of Ti felt after a sintering treatment with PTFE.

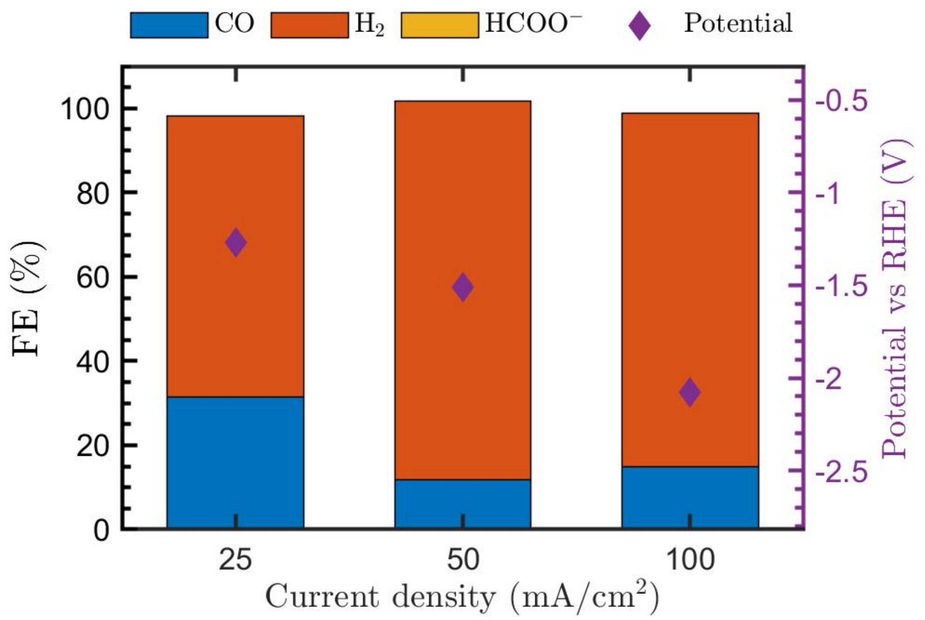

Figure S.5: Faradaic efficiency for a Ag-coated Ti felt. The feed stream consisted of 100 mL/min pure CO_2.

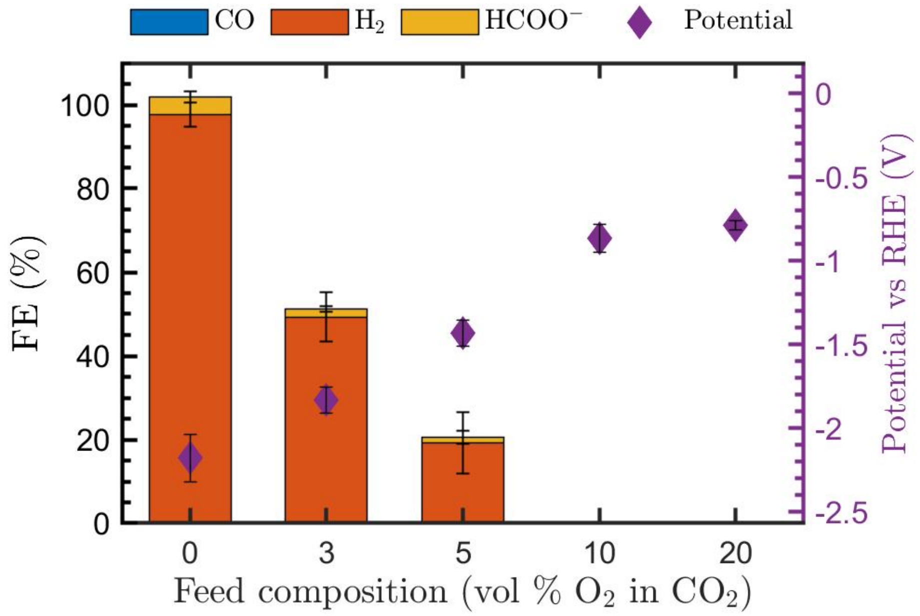

Figure S.6: All results for the bare 39BB carbon-based GDL at 100 mA cm^{-2}, related to Fig. 2.

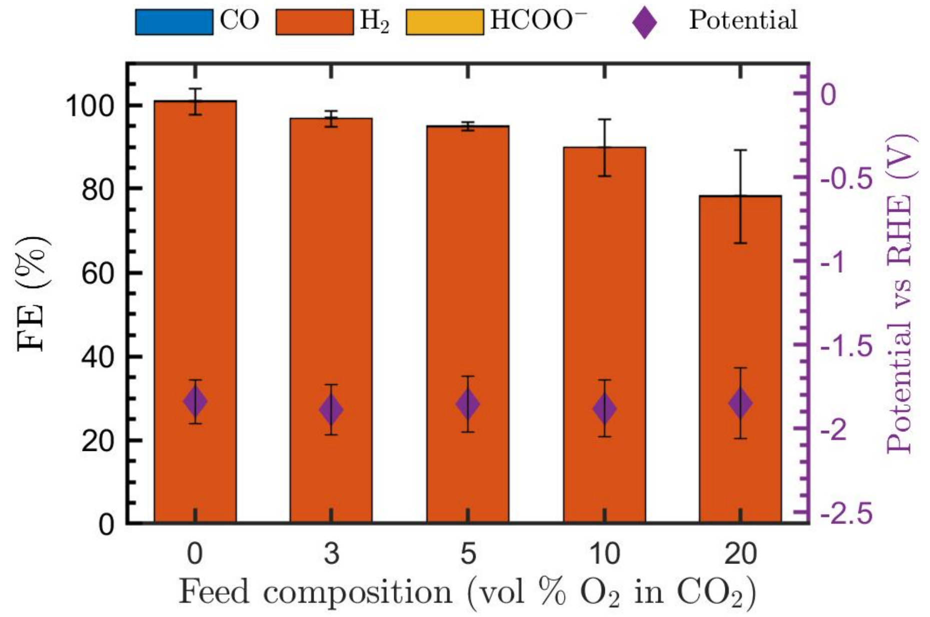

Figure S.7: All results for the bare Ti felt at 100 mA cm^{-2}, related to Fig. 2.

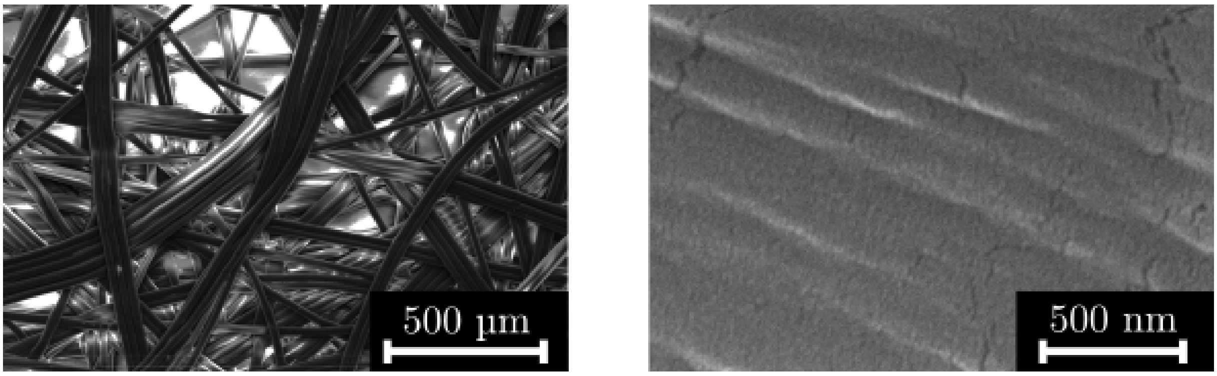

Figure S.8: SEM images of the PP backer at different magnitudes. A thin layer of 25 nm Ag is sputtered on the sample to avoid charging during imaging.

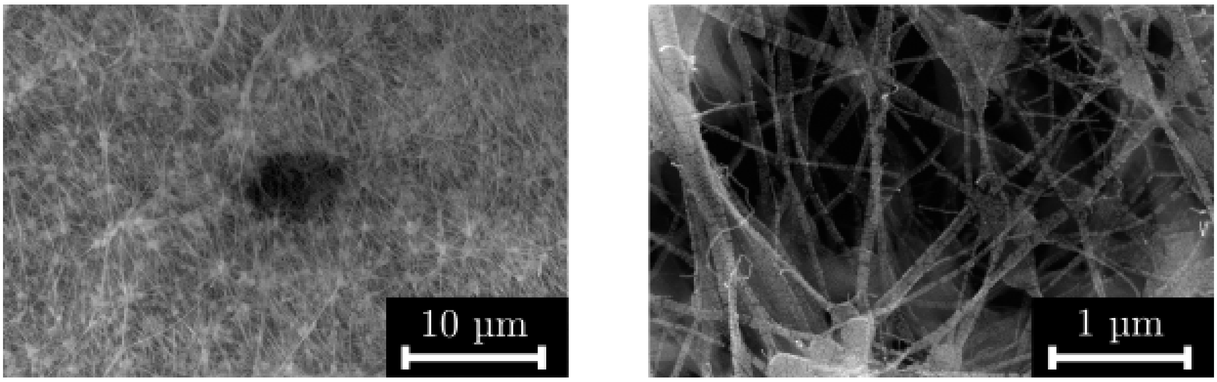

Figure S.9: SEM images of the PTFE side at different magnitudes. A thin layer of 25 nm Ag is sputtered on the sample to avoid charging during imaging.

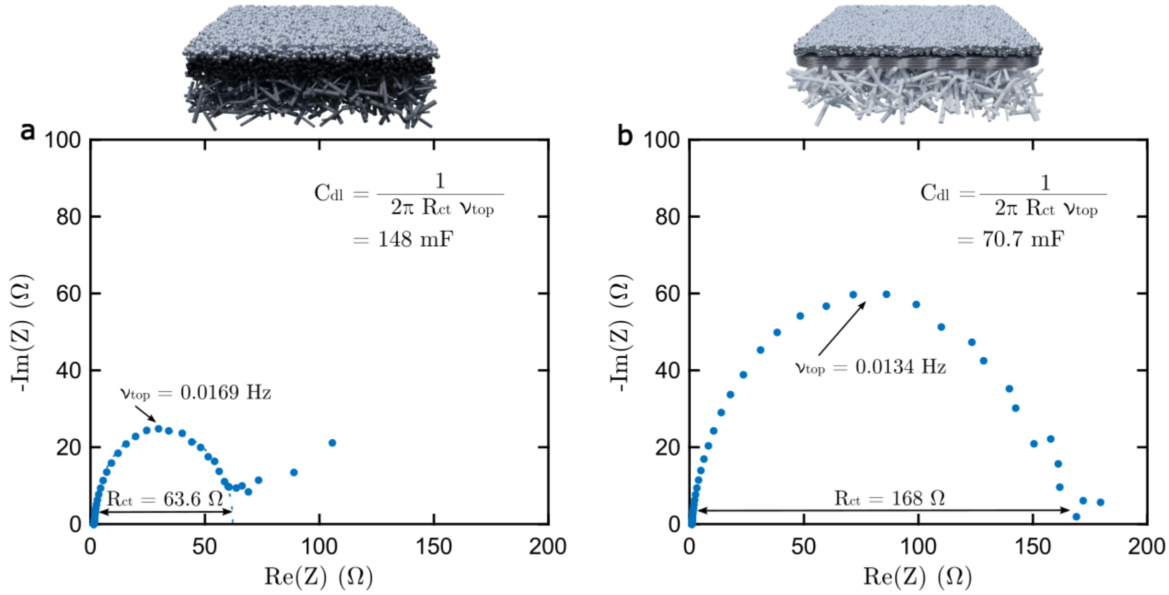

Figure S.10: Electrochemical impedance spectroscopy on the 39BB-Ag and PTFE-Ag GDE to estimate EASA $= \frac{C_{dl}}{C_s}$ with $C_s = 40\ \mu F/cm^2$. **a** 39BB-Ag with an EASA of $3.70 \cdot 10^3\ cm^2$. **b** PTFE-Ag with an EASA of $1.77 \cdot 10^3\ cm^2$.

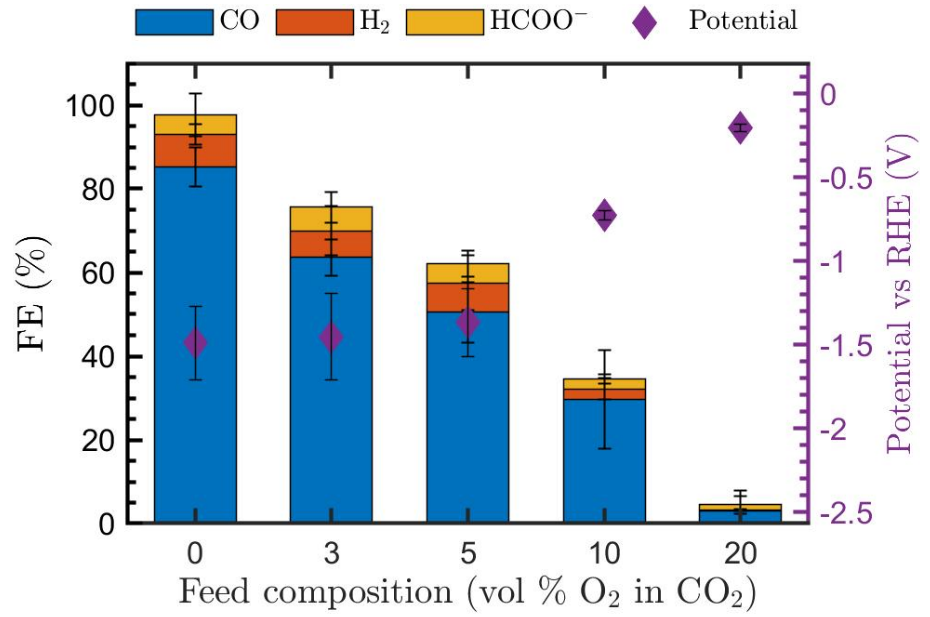

Figure S.11: All results for the Ag-coated PTFE substrate at 100 mA cm^{-2}, related to Fig. 3.

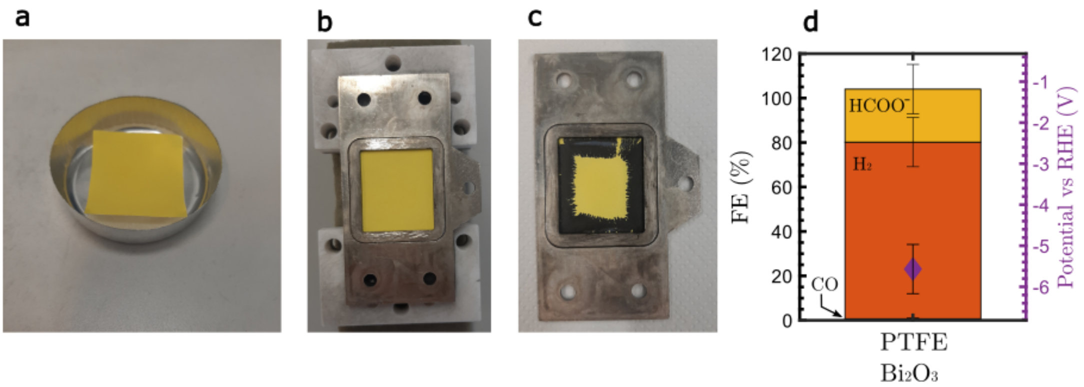

Figure S.12: Pictures and reactor operation with a Bi_2O_3-coated PTFE GDE. **a** Picture of the prepared GDE. **b** Picture of the Cathode frame with the prepared GDE. **c** Unreacted (yellow) and reacted (dark blue/black) areas after reactor operation. **d** Results derived from two independent reactor runs.

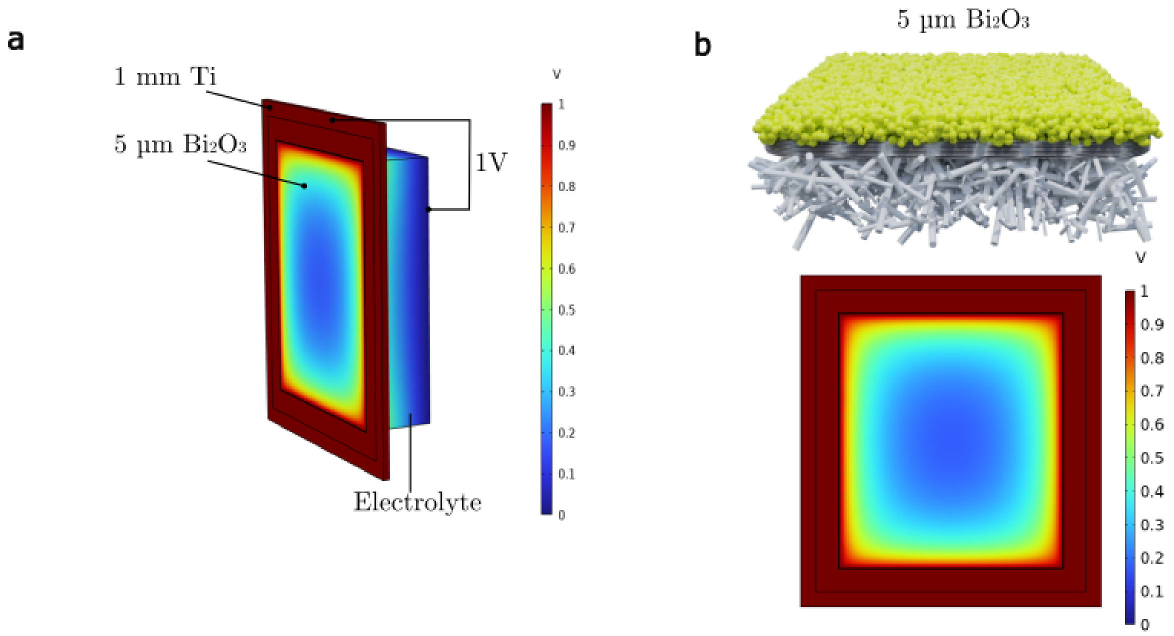

Figure S.13: Potential distribution in the CL of the PTFE-Bi_2O_3 base case. **a** Schematical representation of the model. **b** Potential distribution in the CL plane on the intersection between electrolyte and CL.

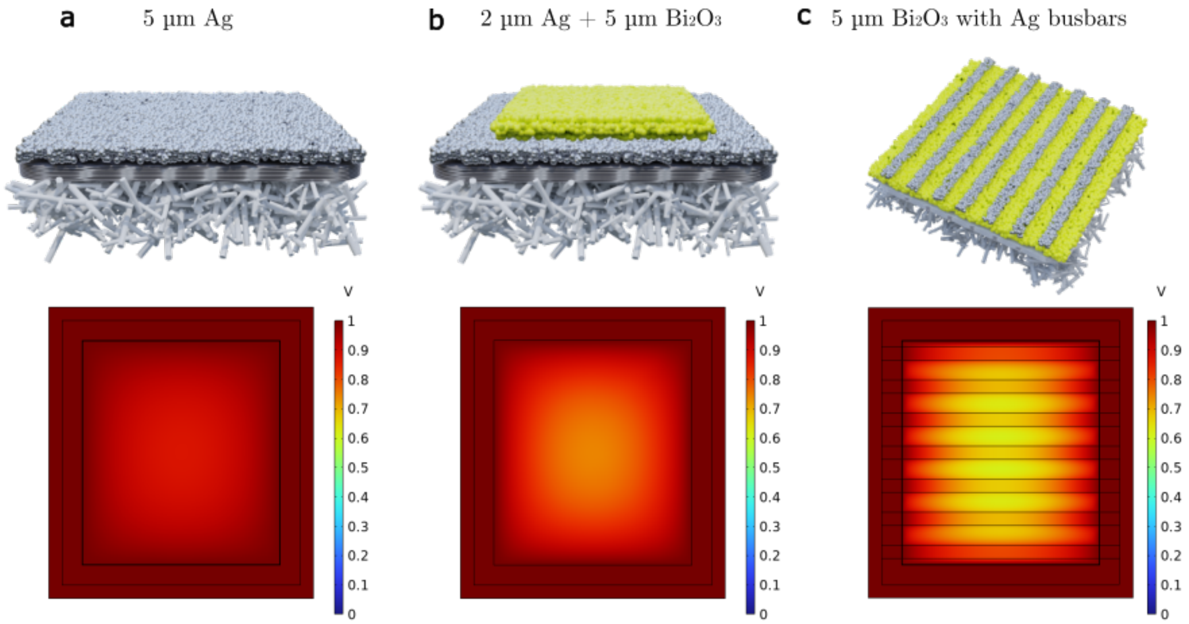

Figure S.14: Potential distribution in the CL for different PTFE GDE configurations. **a** PTFE-Ag. **b** PTFE Bi_2O_3 with a conductive Ag sublayer. **c** PTFE Bi_2O_3 with Ag busbars.

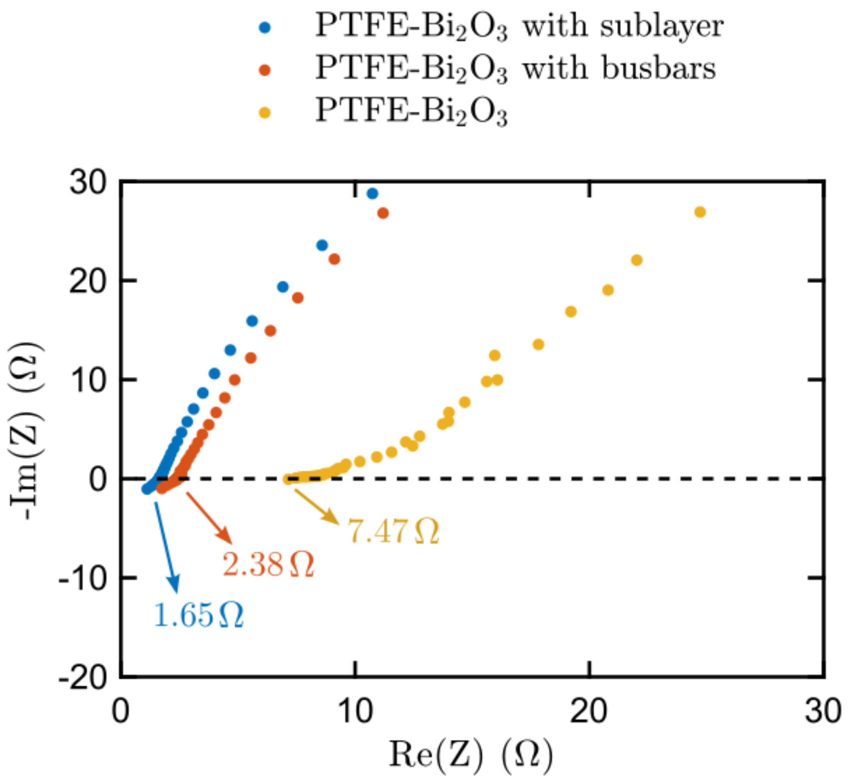

Figure S.15: Comparison of resistance between the two proposed polymer-based GDE designs and the solely Bi_2O_3-coated PTFE GDE through electrochemical impedance spectroscopy.

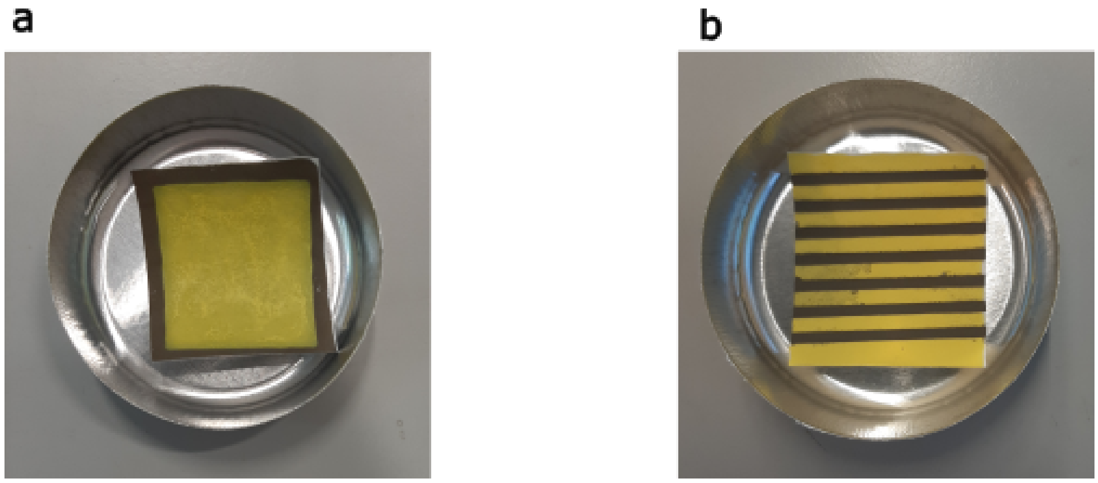

Figure S.16: Pictures of the PTFE-Bi_2O_3 configurations. **a** PTFE-Bi_2O_3 with a conductive Ag sublayer. **b** PTFE-Bi_2O_3 with 7x2 mm Ag busbars.

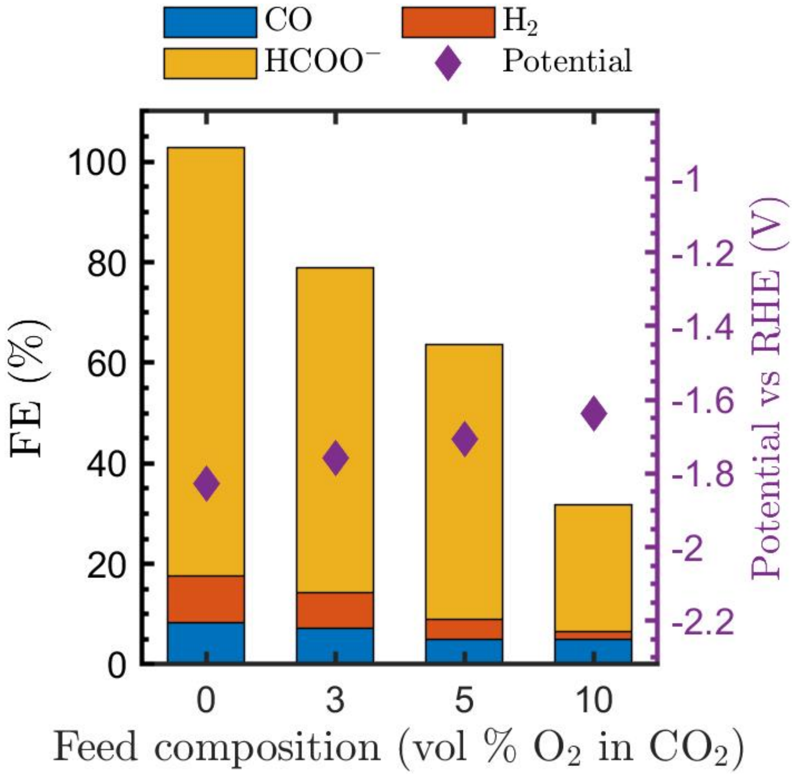

Figure S.17: Experimental results for a PTFE GDE with 1 mg/cm^2 carbon black sublayer with a 2 mg/cm^2 Bi_2O_3 top layer for different oxygen concentrations at 100 mA/cm^2 and 100 mL/min.

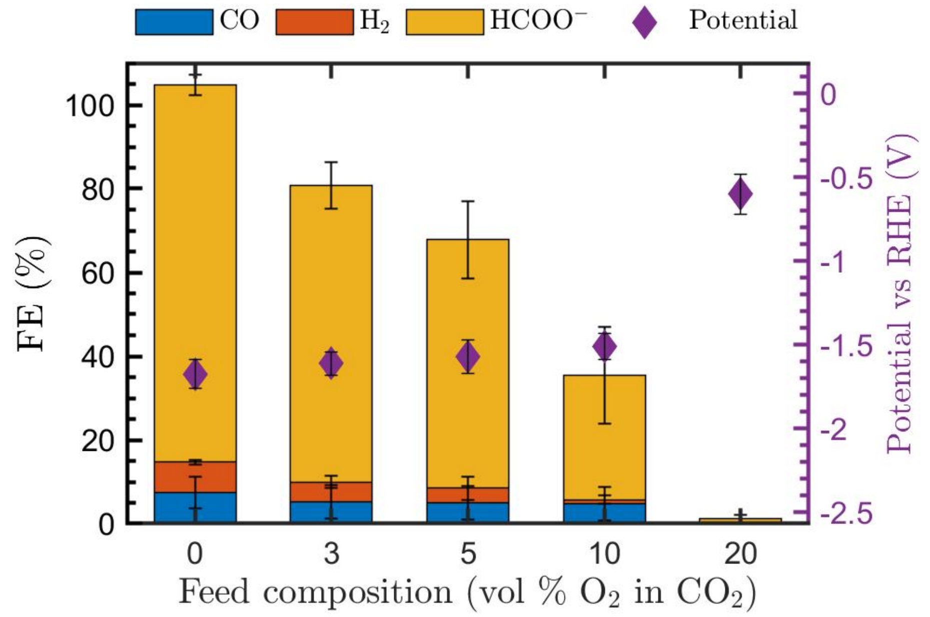

Figure S.18: All results for the PTFE-Bi_2O_3 GDE with a conductive sublayer at 100 mA cm^{-2}, related to Fig. 5.

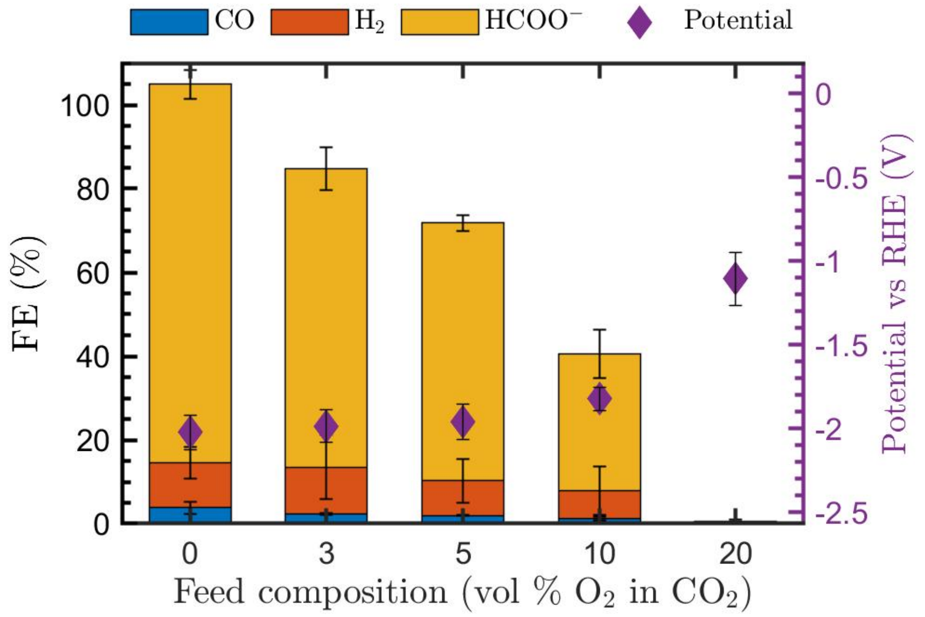

Figure S.19: All results for the PTFE-Bi_2O_3 GDE with busbars at 100 mA cm^{-2}, related to Fig. 5.

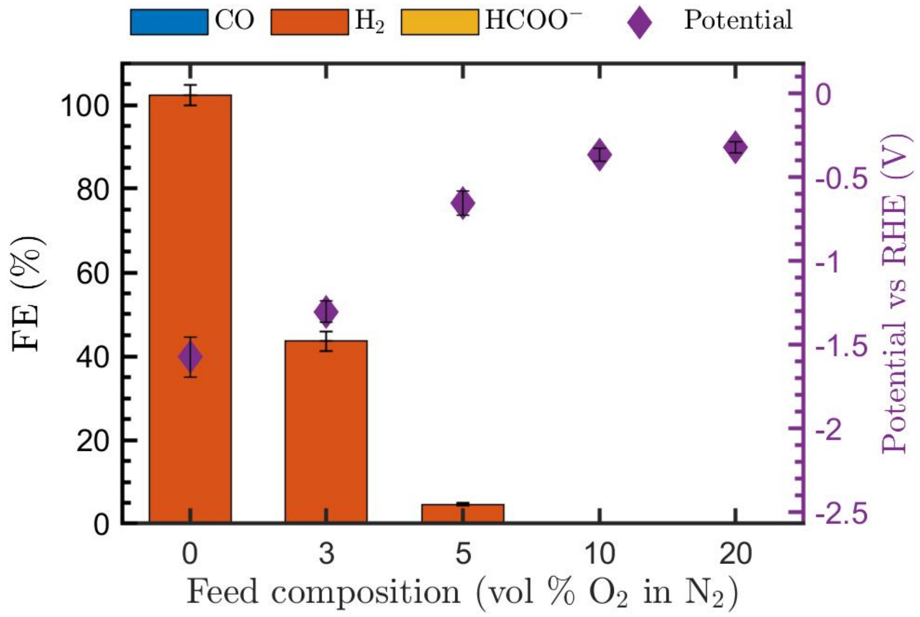

Figure S.20: Results for the 39BB-Ag GDE at 100 mA cm^{-2} with N_2 as balance gas.

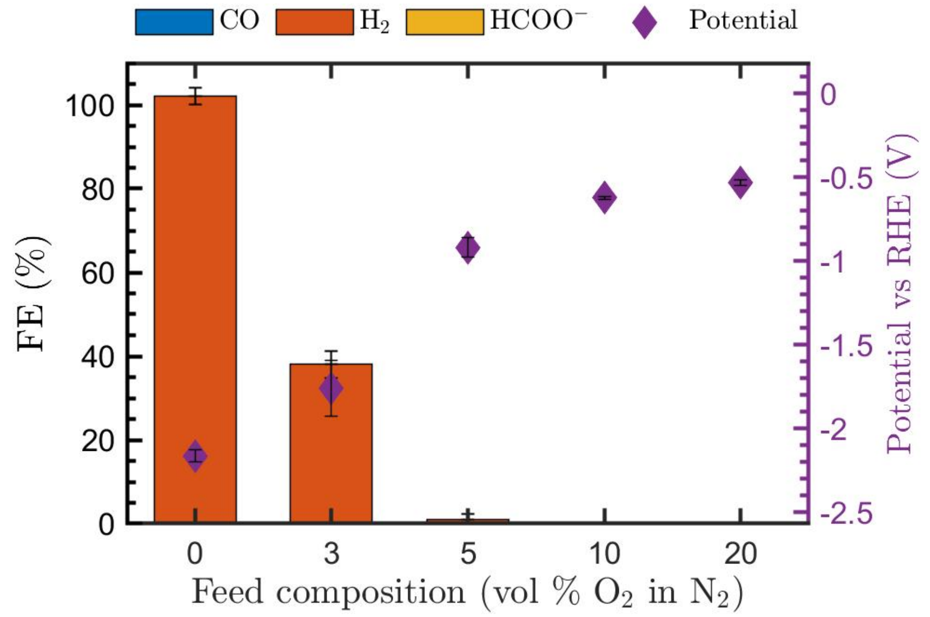

Figure S.21: Results for the 39BB-Bi_2O_3 GDE at 100 mA cm^{-2} with N_2 as balance gas.

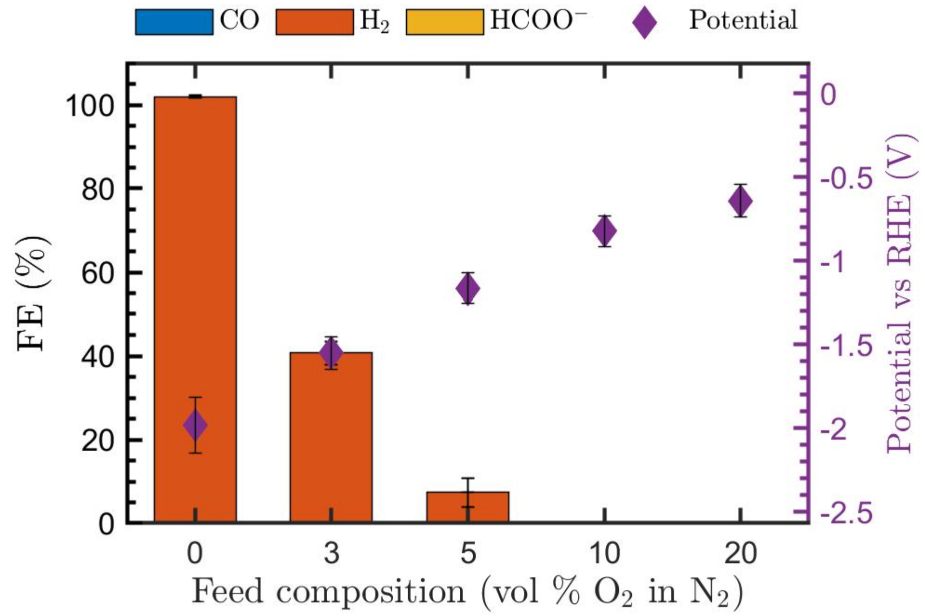

Figure S.22: Results for the bare 39BB GDL at 100 mA cm^{-2} with N_2 as balance gas.

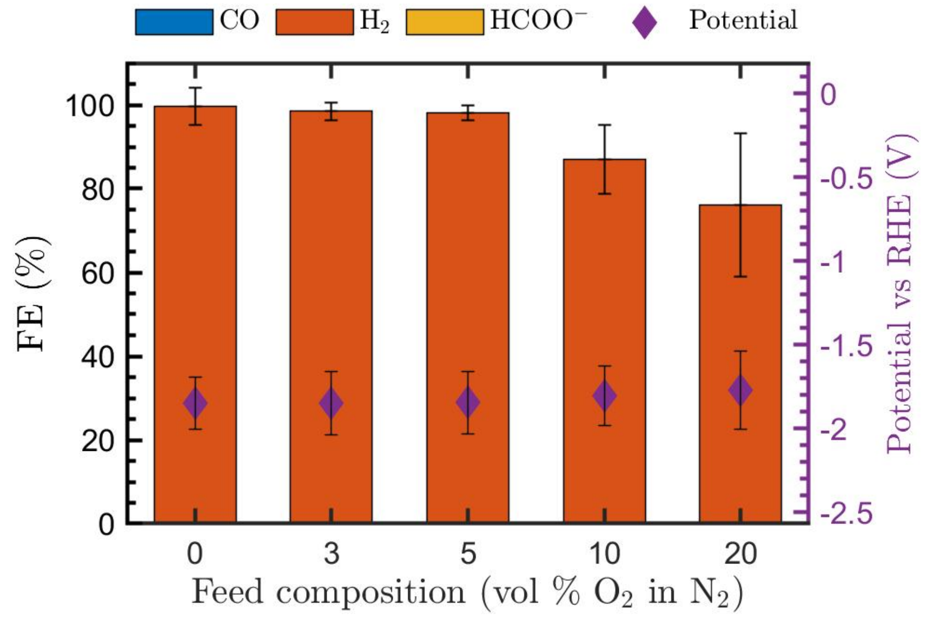

Figure S.23: Results for the bare Ti felt at 100 mA cm^{-2} with N_2 as balance gas.

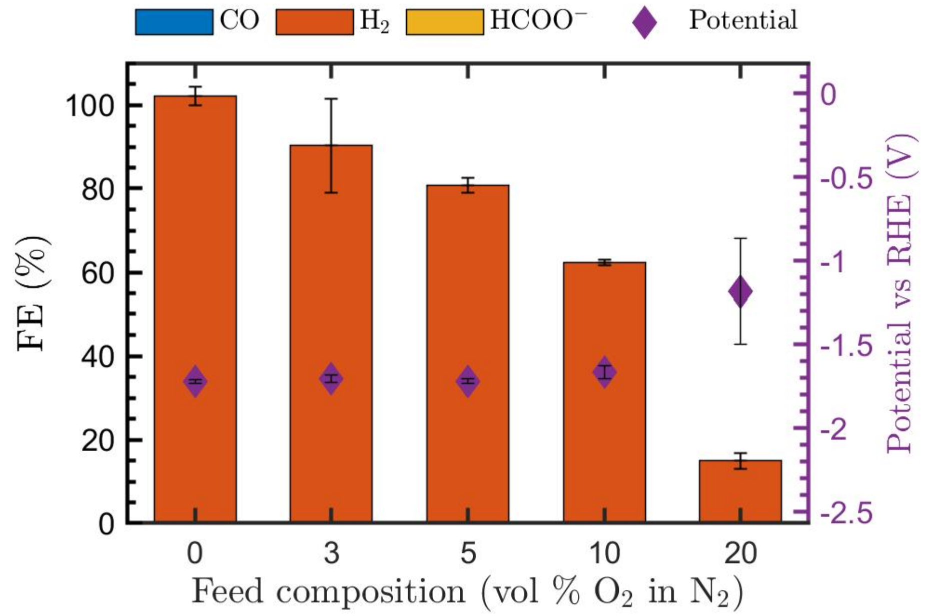

Figure S.24: Results for the PTFE-Ag GDE at 100 mA cm^{-2} with N_2 as balance gas.

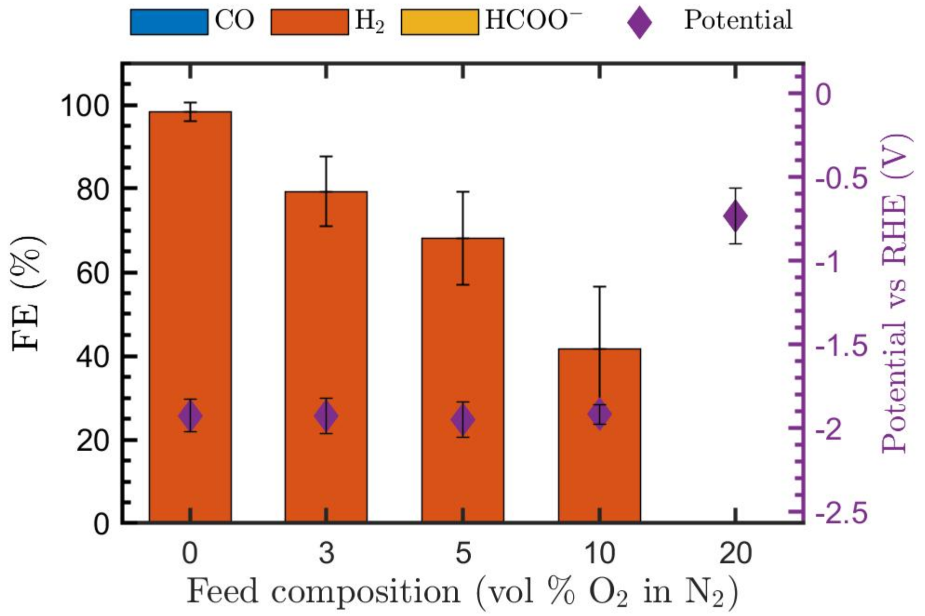

Figure S.25: Results for the PTFE-Bi_2O_3 GDE with a conductive sublayer at 100 mA cm^{-2} with N_2 as balance gas.

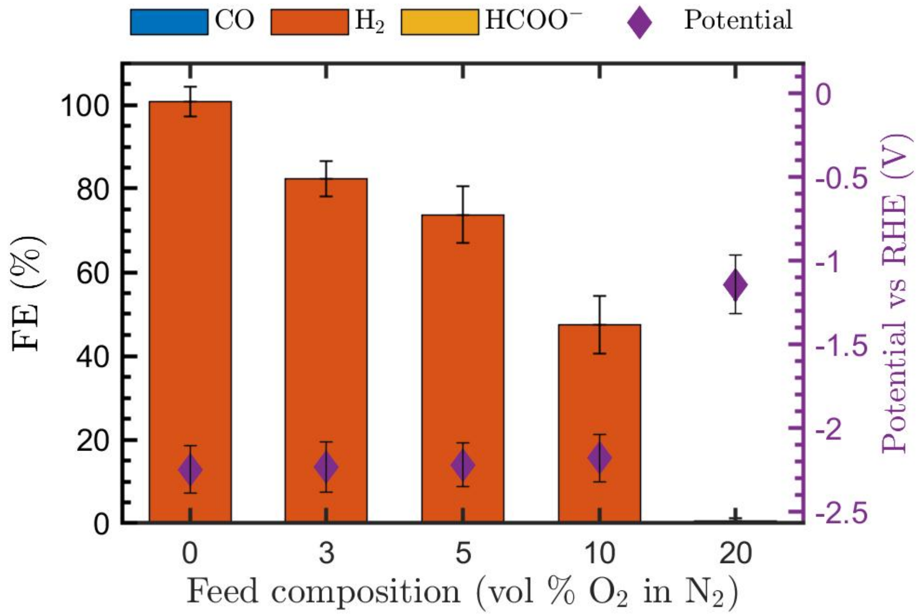

Figure S.26: Results for the PTFE-Bi_2O_3 GDE with busbars at 100 mA cm^{-2} with N_2 as balance gas.

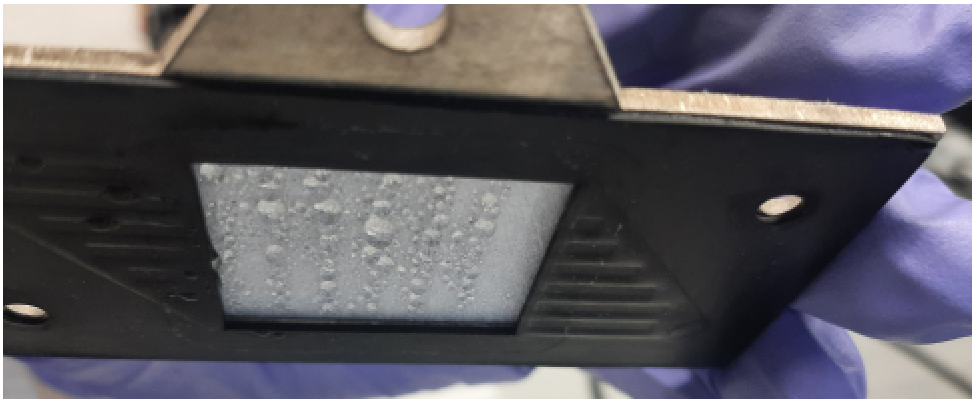

Figure S.27: Picture of the PP side (facing gas chamber) after electrolysis at 100 mA cm^{-2}. The droplets are aligned with the busbars, indicating that perspiration occured mostly in line with the busbars due to electrowetting in these highly conductive zones.

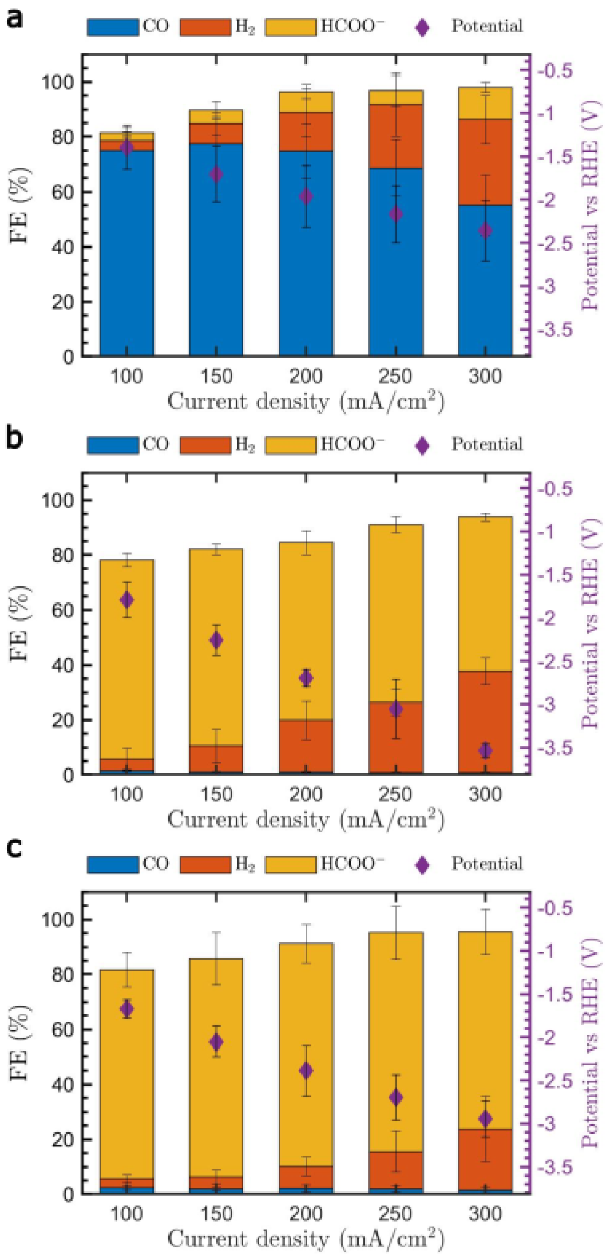

Figure S.28: Faradaic efficiency and potential for a 100 mL/min 3% O_2 in CO_2 feed stream at current densities up to 300 mA/cm^2 for **a** PTFE-Ag, **b** PTFE-Bi_2O_3 sublayer, **c** PTFE-Bi_2O_3 busbars.

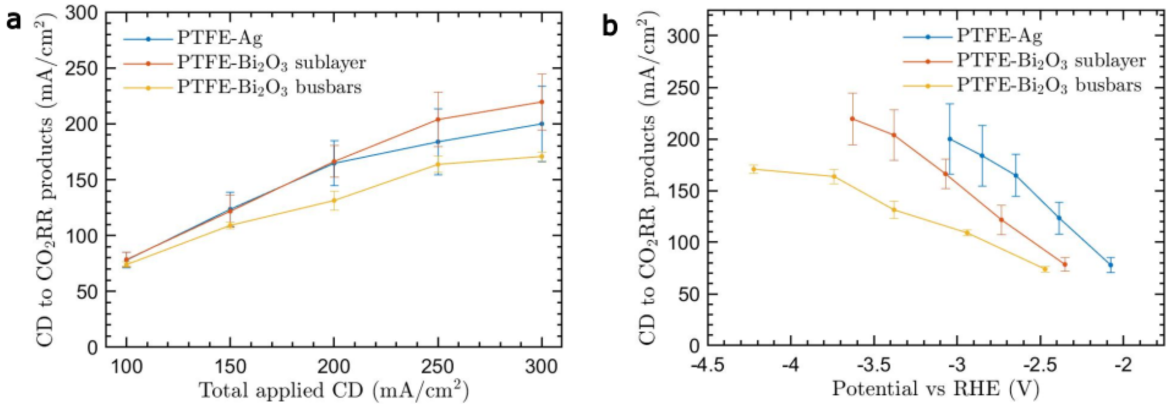

Figure S.29: Partial current density to CO_2RR products for three GDE systems plotted against **a** total applied current density and **b** potential vs RHE. The feed stream consisted of 3% O_2 in CO_2.

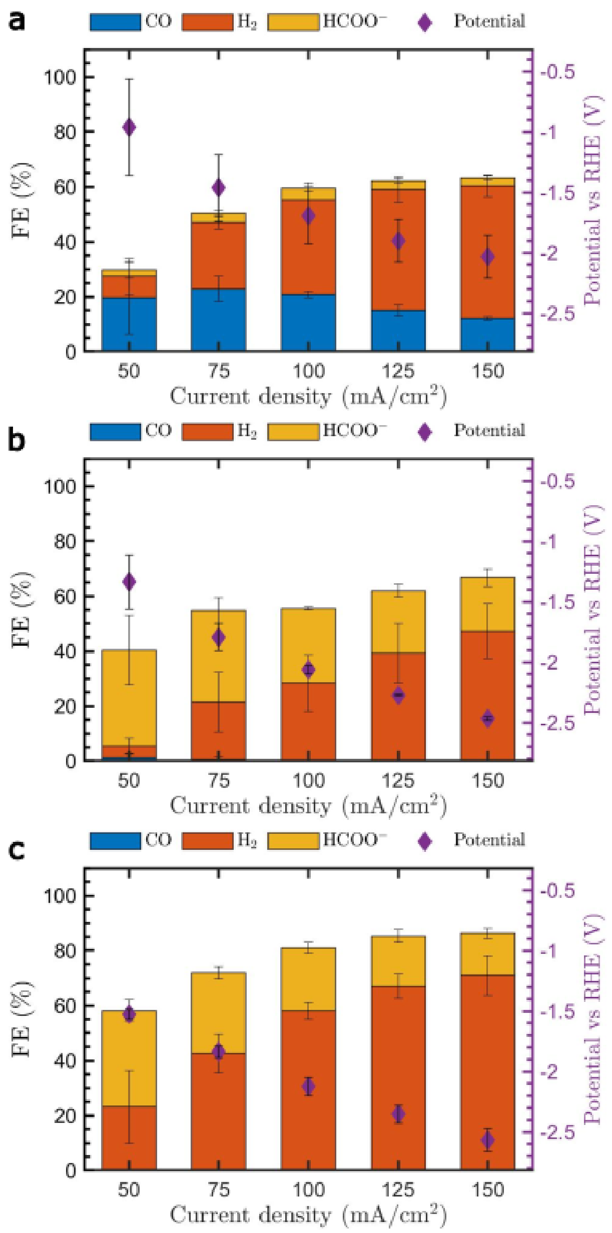

Figure S.30: Faradaic efficiency and potential for a 100 mL/min simulated flue gas (15% CO_2 + 4% O_2 in N_2) as feed stream at current densities up to 150 mA/cm^2 for **a** PTFE-Ag, **b** PTFE-Bi_2O_3 sublayer, **c** PTFE-Bi_2O_3 busbars.

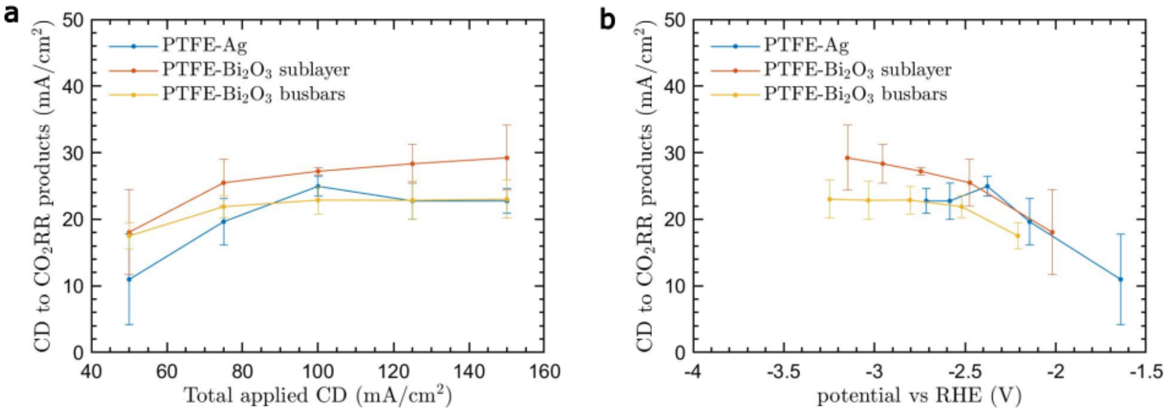

Figure S.31: Partial current density to CO_2RR products for three GDE systems plotted against **a** total applied current density and **b** potential vs RHE. The feed stream consisted of 15% CO_2 + 4% O_2 in N_2.

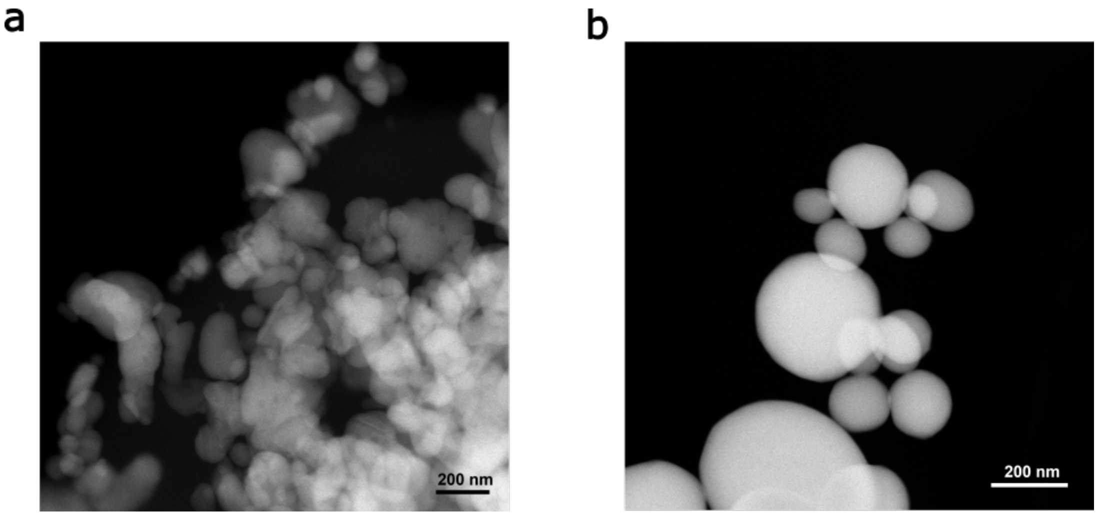

Figure S.32: Transmission electron microscopy images of the catalytic nanoparticles that were used in this work. **a** Ag nanoparticles. **b** Bi_2O_3 nanoparticles.

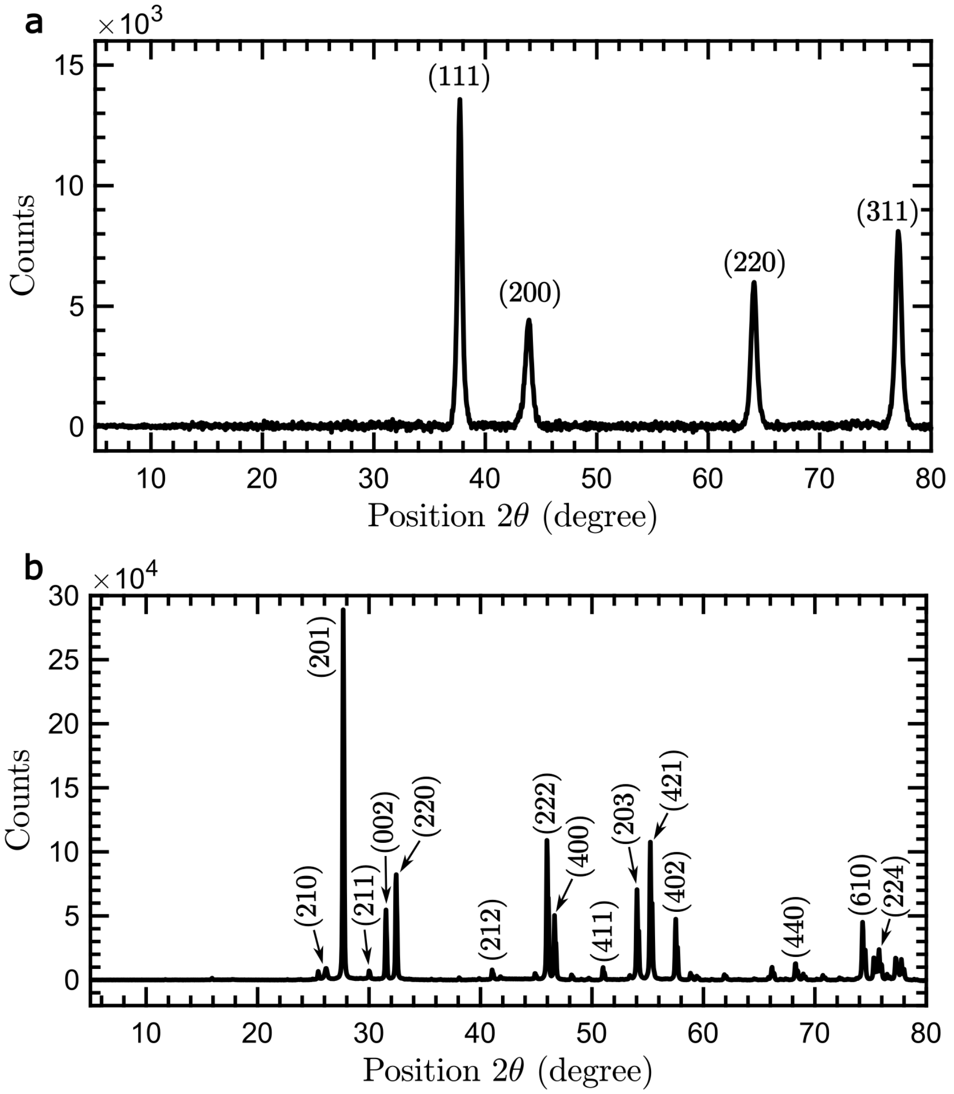

Figure S.33: X-ray diffraction results of the catalytic nanoparticles that were used in this work. **a** Ag nanoparticles, corresponding to JCPDS card number 04-0783. **b** Bi_2O_3 nanoparticles, corresponding to JCPDS card number 27-0050.

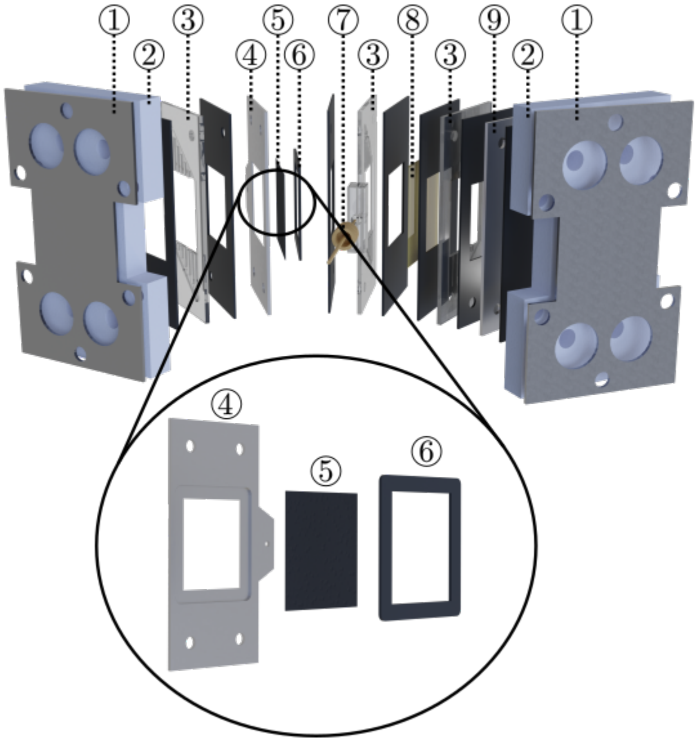

Figure S.34: The electrochemical flow reactor that was used during this study. 1) metal backplate, 2) PTFE backplate 3) PMMA gas- and liquid flow plates, 4) Titanium cathode frame, 5) gas diffusion electrode, 6) Viton gasket for GDE, 7) Ag/AgCl reference electrode, 8) Nafion 117 cation exchange membrane, 9) Platinized titanium electrode. All parts are separated by rubber gaskets. During operation with polymer-based GDEs, the Viton gasket (part 6) is changed to a titanium seal.

Supplemental Tables

Table S.1: Experimental data for the carbon-based 39BB GDE with Ag nanoparticles, related to Fig. 2.

% O_2 in CO_2	FE_{H_2} (%)	σ	FE_{CO} (%)	σ	FE_{HCOO^-} (%)	σ	V vs Ag/AgCl	σ	V vs RHE	FE_{tot} (%)	σ
0	0.87	1.23	95.33	1.35	3.49	2.41	-2.25	0.12	-1.57	99.69	3.03
3	0.56	0.80	46.76	7.19	0.68	0.11	-1.97	0.02	-1.29	48.00	7.24
5	0.16	0.23	10.10	9.71	0.12	0.08	-1.51	0.21	-0.83	10.39	9.71
10	0.00	0.00	0.01	0.01	0.00	0.01	-1.04	0.05	-0.36	0.01	0.01
20	0.00	0.00	0.01	0.02	0.00	0.00	-0.96	0.05	-0.27	0.01	0.02

Table S.2: Experimental data for the carbon-based 39BB GDE with Bi_2O_3 nanoparticles, related to Fig. 2.

% O_2 in CO_2	FE_{H_2} (%)	σ	FE_{CO} (%)	σ	FE_{HCOO^-} (%)	σ	V vs Ag/AgCl	σ	V vs RHE	FE_{tot} (%)	σ
0	0.14	0.20	3.55	0.23	94.98	1.82	-2.32	0.01	-1.64	98.67	1.85
3	0.25	0.01	1.56	0.58	37.26	7.50	-2.13	0.06	-1.44	39.07	7.52
5	0.00	0.00	0.53	0.56	10.76	7.79	-1.87	0.24	-1.19	11.29	7.81
10	0.00	0.00	0.00	0.00	0.00	0.00	-1.57	0.21	-0.89	0.00	0.00
20	0.00	0.00	0.00	0.00	0.00	0.00	-1.35	0.01	-0.67	0.00	0.00

Table S.3: Experimental data for the bare 39BB carbon-based GDL, related to Fig. 2.

% O_2 in CO_2	FE_{H_2} (%)	σ	FE_{CO} (%)	σ	FE_{HCOO^-} (%)	σ	V vs Ag/AgCl	σ	V vs RHE	FE_{tot} (%)	σ
0	97.55	2.95	0.12	0.12	4.23	1.39	-2.86	0.14	-2.18	101.89	3.26
3	49.18	5.90	0.07	0.07	2.02	0.73	-2.51	0.08	-1.83	51.27	5.95
5	19.29	7.32	0.03	0.04	1.29	1.57	-2.12	0.08	-1.43	20.61	7.49
10	0.00	0.00	0.00	0.00	0.00	0.00	-1.55	0.08	-0.86	0.00	0.00
20	0.00	0.00	0.00	0.00	0.00	0.00	-1.47	0.03	-0.79	0.00	0.00

Table S.4: Experimental data for the bare Ti felt after hydrophobic treatment, related to Fig. 2.

% O_2 in CO_2	FE_{H_2} (%)	σ	FE_{CO} (%)	σ	FE_{HCOO^-} (%)	σ	V vs Ag/AgCl	σ	V vs RHE	FE_{tot} (%)	σ
0	100.82	3.14	0.00	0.00	0.10	0.10	-2.52	0.13	-1.84	100.92	3.14
3	96.73	1.84	0.00	0.00	0.09	0.08	-2.57	0.15	-1.89	96.82	1.85
5	94.88	1.09	0.00	0.00	0.14	0.02	-2.54	0.16	-1.85	95.02	1.09
10	89.81	6.84	0.00	0.00	0.09	0.07	-2.56	0.17	-1.88	89.90	6.85
20	78.12	11.07	0.00	0.00	0.13	0.01	-2.53	0.21	-1.85	78.24	11.07

Table S.5: Experimental data for the Ag-coated PTFE substrate, related to Fig. 3.

% O_2 in CO_2	FE_{H_2} (%)	σ	FE_{CO} (%)	σ	FE_{HCOO^-} (%)	σ	V vs Ag/AgCl	σ	V vs RHE	FE_{tot} (%)	σ
0	7.81	2.50	85.17	4.63	4.70	5.20	-2.17	0.22	-1.49	97.67	7.40
3	6.38	5.94	63.57	4.38	5.62	3.57	-2.13	0.26	-1.45	75.57	8.20
5	7.07	6.61	50.47	7.26	4.55	3.07	-2.05	0.20	-1.37	62.09	10.28
10	2.62	2.62	29.59	11.76	2.31	1.11	-1.41	0.03	-0.73	34.52	12.10
20	0.18	0.25	3.01	4.91	1.26	2.18	-0.89	0.02	-0.21	4.45	5.38

Table S.6: Experimental data for the PTFE-Bi_2O_3 GDE with a conductive sublayer, related to Fig. 5.

% O_2 in CO_2	FE_{H_2} (%)	σ	FE_{CO} (%)	σ	FE_{HCOO^-} (%)	σ	V vs Ag/AgCl	σ	V vs RHE	FE_{tot} (%)	σ
0	7.14	0.62	7.55	3.78	90.12	2.43	-2.36	0.09	0.77	104.81	4.54
3	4.81	1.46	5.13	4.00	70.90	5.59	-2.29	0.07	0.75	80.84	7.03
5	3.47	2.71	5.00	4.05	59.33	9.13	-2.25	0.10	0.78	67.79	10.35
10	0.95	1.08	4.81	4.02	29.75	11.60	-2.19	0.08	0.76	35.51	12.32
20	0.00	0.00	0.00	0.00	1.12	1.00	-1.28	0.12	0.80	1.12	1.00

Table S.7: Experimental data for the PTFE-Bi_2O_3 GDE with conductive busbars, related to Fig. 5.

% O_2 in CO_2	FE_{H_2} (%)	σ	FE_{CO} (%)	σ	FE_{HCOO^-} (%)	σ	V vs Ag/AgCl	σ	V vs RHE	FE_{tot} (%)	σ
0	10.72	3.73	3.81	1.41	90.38	3.45	-2.70	0.10	-2.02	104.91	5.27
3	11.15	7.65	2.34	0.24	71.35	5.09	-2.67	0.09	-1.98	84.84	9.20
5	8.33	5.22	2.00	0.22	61.51	1.88	-2.64	0.11	-1.96	71.84	5.55
10	6.69	5.82	1.27	0.41	32.65	5.72	-2.51	0.07	-1.82	40.61	8.17
20	0.00	0.00	0.13	0.18	0.32	0.46	-1.79	0.16	-1.11	0.45	0.49

Table S.8: Experimental data for 39BB-Ag GDE with N_2 as balance gas.

% O_2 in N_2	FE_{H_2} (%)	σ	FE_{CO} (%)	σ	FE_{HCOO^-} (%)	σ	V vs Ag/AgCl	σ	V vs RHE	FE_{tot} (%)	σ
0	102.37	2.48	0.00	0.00	0.00	0.00	-2.26	0.12	-1.57	102.37	2.48
3	43.59	2.38	0.00	0.00	0.00	0.00	-1.99	0.06	-1.30	43.59	2.38
5	4.67	0.41	0.00	0.00	0.00	0.00	-1.34	0.07	-0.65	4.67	0.41
10	0.00	0.00	0.00	0.00	0.00	0.00	-1.05	0.04	-0.37	0.00	0.00
20	0.00	0.00	0.00	0.00	0.00	0.00	-1.00	0.03	-0.32	0.00	0.00

Table S.9: Experimental data for 39BB-Bi_2O_3 GDE with N_2 as balance gas.

% O_2 in N_2	FE_{H_2} (%)	σ	FE_{CO} (%)	σ	FE_{HCOO^-} (%)	σ	V vs Ag/AgCl	σ	V vs RHE	FE_{tot} (%)	σ
0	102.14	1.97	0.00	0.00	0.00	0.00	-2.85	0.04	-2.16	102.14	1.97
3	38.00	3.19	0.00	0.00	0.00	0.00	-2.44	0.17	-1.76	38.00	3.19
5	1.01	1.42	0.00	0.00	0.00	0.00	-1.60	0.06	-0.92	1.01	1.42
10	0.00	0.00	0.00	0.00	0.00	0.00	-1.31	0.01	-0.62	0.00	0.00
20	0.00	0.00	0.00	0.00	0.00	0.00	-1.21	0.02	-0.53	0.00	0.00

Table S.10: Experimental data for a bare 39BB GDL with N_2 as balance gas.

% O_2 in N_2	FE_{H_2} (%)	σ	FE_{CO} (%)	σ	FE_{HCOO^-} (%)	σ	V vs Ag/AgCl	σ	V vs RHE	FE_{tot} (%)	σ
0	101.94	0.34	0.00	0.00	0.00	0.00	-2.66	0.17	-1.98	101.94	0.34
3	40.70	2.73	0.00	0.00	0.00	0.00	-2.23	0.10	-1.55	40.70	2.73
5	7.34	3.41	0.00	0.00	0.00	0.00	-1.85	0.09	-1.16	7.34	3.41
10	0.00	0.00	0.00	0.00	0.00	0.00	-1.51	0.09	-0.82	0.00	0.00
20	0.00	0.00	0.00	0.00	0.00	0.00	-1.32	0.10	-0.64	0.00	0.00

Table S.11: Experimental data for the bare Ti felt with N_2 as balance gas.

% O_2 in N_2	FE_{H_2} (%)	σ	FE_{CO} (%)	σ	FE_{HCOO^-} (%)	σ	V vs Ag/AgCl	σ	V vs RHE	FE_{tot} (%)	σ
0	99.63	4.40	0.00	0.00	0.00	0.00	-2.53	0.16	-1.85	99.63	4.40
3	98.49	2.12	0.00	0.00	0.00	0.00	-2.53	0.19	-1.85	98.49	2.12
5	98.13	1.69	0.00	0.00	0.00	0.00	-2.53	0.19	-1.85	98.13	1.69
10	87.07	8.23	0.00	0.00	0.00	0.00	-2.49	0.18	-1.80	87.07	8.23
20	76.22	17.11	0.00	0.00	0.00	0.00	-2.46	0.23	-1.77	76.22	17.11

Table S.12: Experimental data for PTFE-Ag GDE with N_2 as balance gas.

% O_2 in N_2	FE_{H_2} (%)	σ	FE_{CO} (%)	σ	FE_{HCOO^-} (%)	σ	V vs Ag/AgCl	σ	V vs RHE	FE_{tot} (%)	σ
0	102.08	2.21	0.00	0.00	0.00	0.00	-2.40	0.01	-1.72	102.08	2.21
3	90.32	11.21	0.00	0.00	0.00	0.00	-2.39	0.02	-1.70	90.32	11.21
5	80.71	1.75	0.00	0.00	0.00	0.00	-2.40	0.01	-1.72	80.71	1.75
10	62.25	0.65	0.00	0.00	0.00	0.00	-2.35	0.04	-1.67	62.25	0.65
20	14.89	1.84	0.00	0.00	0.00	0.00	-1.86	0.32	-1.18	14.89	1.84

Table S.13: Experimental data for PTFE-Bi_2O_3 GDE with a conductive sublayer and N_2 as balance gas.

% O_2 in N_2	FE_{H_2} (%)	σ	FE_{CO} (%)	σ	FE_{HCOO^-} (%)	σ	V vs Ag/AgCl	σ	V vs RHE	FE_{tot} (%)	σ
0	98.38	2.22	0.00	0.00	0.00	0.00	-2.61	0.10	-1.93	98.38	2.22
3	79.29	8.38	0.00	0.00	0.00	0.00	-2.61	0.11	-1.93	79.29	8.38
5	68.15	11.07	0.00	0.00	0.00	0.00	-2.63	0.11	-1.95	68.15	11.07
10	41.64	14.94	0.00	0.00	0.00	0.00	-2.60	0.06	-1.92	41.64	14.94
20	0.00	0.00	0.00	0.00	0.00	0.00	-1.41	0.17	-0.73	0.00	0.00

Table S.14: Experimental data for PTFE-Bi_2O_3 GDE with busbars and N_2 as balance gas.

% O_2 in CO_2	FE_{H_2} (%)	σ	FE_{CO} (%)	σ	FE_{HCOO^-} (%)	σ	V vs Ag/AgCl	σ	V vs RHE	FE_{tot} (%)	σ
0	100.80	3.47	0.00	0.00	0.00	0.00	-2.93	0.14	-2.25	100.80	3.47
3	82.29	4.26	0.00	0.00	0.00	0.00	-2.92	0.15	-2.23	82.29	4.26
5	73.75	6.83	0.00	0.00	0.00	0.00	-2.90	0.13	-2.22	73.75	6.83
10	47.47	6.85	0.00	0.00	0.00	0.00	-2.86	0.14	-2.18	47.47	6.85
20	0.50	0.70	0.00	0.00	0.00	0.00	-1.82	0.17	-1.14	0.50	0.70

Table S.15: ICP-MS measurements on the catholyte outflow after 50 h stability measurements.

	Ag (ppm)	Bi (ppm)
Blank 0.5 M $KHCO_3$ catholyte	Not detected.	$6.27 \cdot 10^{-5}$
PTFE-Ag	Not detected.	$2.32 \cdot 10^{-3}$
PTFE-Bi_2O_3 with Ag sublayer	Not detected.	$1.02 \cdot 10^{-2}$
PTFE-Bi_2O_3 with Ag busbars	Not detected.	$8.16 \cdot 10^{-4}$

Table S.16: In-plane (IP) and through-plane (TP) properties of the Sigracet 39BB gas diffusion layer according to the manufacturer SGL carbon.

Property	Value	Unit
Thickness	315	µm
TP gas permeability	1.5	Gurley sec
TP gas permeability at 1 Mpa	0.12	10^{-12} m^2
IP gas permeability	8.4	10^{-12} m^2
TP area specific resistance (1 MPa)	< 13	mΩ cm^2
IP electric resistance	0.56	Ω mm
TP thermal conductivity	0.2	$Wm^{-1}K^{-1}$

Table S.17: Properties of the Aspire laminated PTFE filter membrane with 0.2 µm pores according to the manufacturer Sterlitech.

Property	Value	Unit
Thickness	152-254	µm
Clean air flow at 70 mbar	0.20-0.51	L/min·cm
Clean water flow at 0.7 bar	0	mL/min·cm
Water entry pressure	> 45	psi
